# Precision Nanomedicine for Traumatic Neural Injury: Functional Paradigms and Translational Frameworks

**DOI:** 10.1002/advs.78308

**Published:** 2026-10-22

**Authors:** Mouyuan Sun, Shuangyang Li, Zhixu He, Jingyi Xie, Yaxian Luo, Xuankai Fan, Jingyu Zhang, Luying Qin, Lianjie Peng, Tao Qiu, Tian Zhang, Huiming Wang, Mengfei Yu

**Affiliations:** ^1^ School of Stomatology Zhejiang Provincial Clinical Research Center for Oral Diseases Stomatology Hospital Zhejiang University School of Medicine Zhejiang Key Laboratory of Oral Biomedical Hangzhou China

**Keywords:** clinical translation, nanoparticle, neural regeneration, peripheral nerve injury, spinal cord injury, traumatic brain injury, traumatic neural injury

## Abstract

Traumatic neural injuries, including traumatic brain injury (TBI), spinal cord injury (SCI), and peripheral nerve injury (PNI), impose a substantial global burden. These injuries converge on oxidative stress, neuroinflammation, regulated cell death, demyelination, and extracellular matrix remodeling, while diverging in timing, cellular effectors, and regenerative capacity. Current management still fails to arrest secondary injury or restore functional neural circuitry. Nanomedicine integrates targeted delivery, microenvironmental regulation, and regenerative support, yet existing strategies and reviews remain compartmentalized by injury type or material class. Such fragmentation constrains the integration of shared mechanisms with injury‐specific requirements. To address this gap, we develop a cross‐injury framework for nanoparticle‐based intervention. Separate reanalysis of three single‐cell datasets suggests recurrent myeloid involvement and injury‐specific within‐dataset patterns. We compare lipid‐based, polymeric, inorganic, carbon‐based, biomimetic, and hybrid platforms in relation to physicochemical properties and biological fate. Six paradigms are organized by pathological need: scaffold construction and microenvironment remodeling, topographical guidance, biomimetic targeting, endogenous stimulus‐responsive release, external physical‐field activation, and intracellular reprogramming. We trace evolution from passive encapsulation to multi‐omics‐guided and artificial intelligence (AI)‐assisted precision design and examine barriers to manufacturing, biodistribution, safety, protein‐corona control, and regulatory evaluation. This framework connects injury mechanisms, functional needs, and material selection to guide translation.

## Graphical abstract

This review presents a pathology‐driven framework unifying nanomedicine across TBI, SCI, and PNI. Epidemiological evidence and comparative single‐cell transcriptomic reanalysis define shared pathological modules, injury‐specific effectors, and therapeutic bottlenecks. Six functional paradigms connect nanomaterial structure and biological fate to injury‐specific requirements, while a translational roadmap moves toward responsive, multi‐omics‐guided, and AI‐assisted precision neural nanomedicine.

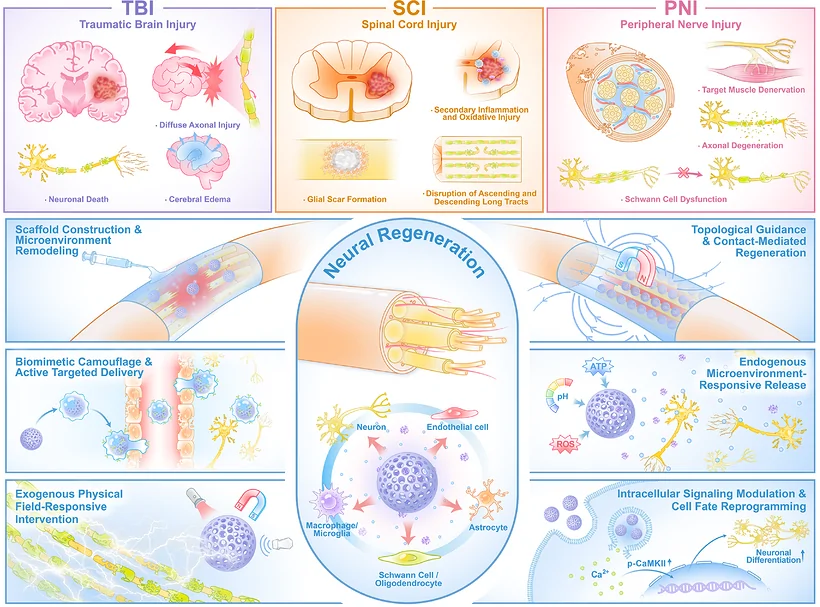

## 1 Introduction

Traumatic brain injury (TBI), spinal cord injury (SCI), and peripheral nerve injury (PNI) constitute the three predominant categories of neurotrauma and collectively impose a formidable global burden of mortality, enduring disability, and socioeconomic decline. Annually, TBI and SCI account for approximately 27.1 million and 0.91 million incident cases, respectively, whereas PNI occurs at an incidence of 13 to 23 cases per 100 000 person‐years within developed healthcare infrastructures [1, 2, 3]. Despite distinct anatomical environments, barrier properties, and regenerative capacities, these injuries converge on a shared secondary pathological architecture involving vascular and barrier disruption, oxidative and metabolic stress, neuroinflammation, regulated cell death, axonal and myelin degeneration, and extracellular matrix remodeling [4]. The temporal progression and dominant cellular effectors of these processes nonetheless differ substantially across TBI, SCI, and PNI, generating injury‐specific therapeutic bottlenecks. Current clinical interventions can address acute physiological instability or anatomical disruption yet remain limited in controlling these dynamic and interconnected secondary processes while restoring functional neural circuitry [5, 6, 7]. Three interrelated challenges consequently remain central to traumatic neural restoration: effective therapeutic delivery within anatomically constrained tissues, regulation of spatiotemporally evolving pathological networks, and durable neural regeneration with circuit reconstruction. Concerted resolution of these challenges demands therapeutic strategies that integrate delivery, pathological regulation, and regenerative support.

Nanomedicine provides an exceptionally programmable platform for addressing these barriers in concert. Moving beyond conventional small‐molecule agents and freely administered biologics, nanomaterials function not as passive drug carriers but as precisely tunable systems whose dimensional attributes, surface chemistry, mechanical properties, electrical or magnetic characteristics, and degradation kinetics can be engineered to translate material properties into defined therapeutic functions [8]. Six interconnected capabilities hold particular relevance for traumatic neural restoration. Nanomaterials shield therapeutic cargos from premature degradation, promote transport across biological barriers, permit lesion‐selective or cell‐selective delivery, and furnish physical or topographical support for cellular migration and axonal growth [9, 10, 11, 12]. These platforms further transduce magnetic, optical, electrical, or ultrasonic energy into localized stimuli, thereby modulating intracellular signaling networks and cell fate [13, 14]. In parallel, nanotherapeutic strategies have traversed a generational trajectory. Early platforms operated largely as passive vehicles that protected and transported therapeutic cargo; subsequent generations incorporated ligand‐mediated active targeting, biomimetic camouflage, and stimulus‐responsive release [15]; emerging approaches now seek to integrate nanomaterial engineering with single‐cell omics, multi‐omics, and data‐driven precision therapy [16]. A representative illustration involves Ce‐doped upconversion antioxidant nanoenzymes, which, when profiled via single‐nucleus RNA sequencing (snRNA‐seq) and single‐nucleus assay for transposase‐accessible chromatin sequencing (snATAC‐seq), not only modulated reactive oxygen species (ROS) levels but also uncovered treatment‐associated alterations in myelinating oligodendrocytes and regulatory programs during spinal cord restoration [17]. Neural nanomedicine is therefore advancing from a rudimentary drug‐carrier paradigm toward multifunctional systems capable of orchestrating delivery, signaling regulation, and structural restoration.

Notwithstanding this accelerated expansion, the prevailing knowledge architecture remains fragmented. A substantial proportion of extant reviews are organized around a single injury category or a particular material class [18, 19], and while such contributions afford valuable syntheses within their respective domains, they remain ill‐suited to distilling transferable design principles across anatomically distinct neural insults [20]. Several conceptual discontinuities consequently persist. Mechanistic discovery and nanomaterial target design remain inadequately coupled: single‐cell and multi‐omics platforms now resolve dynamic cell states and molecular programs across post‐injury phases, yet systematic frameworks for translating these readouts into cellular targets, therapeutic windows, or payload combinations remain scarce [21, 22]. Therapeutic function and material selection likewise remain decoupled in conventional practice, because materials frequently undergo initial selection, subsequent property optimization, and only then application matching. A pathology‐driven strategy would instead commence with the dominant therapeutic bottleneck, define the requisite function, and select the most suitable material platform [23]. TBI, SCI, and PNI are also largely studied as separate fields, masking shared pathological modules such as oxidative stress, neuroinflammation, cell death, demyelination, and scarring, and obscuring opportunities for cross‐injury transfer of established nanotechnologies [24]. A complete translational loop from material design to clinical implementation remains underdeveloped, constrained by persistent challenges in physicochemical characterization, preclinical reproducibility, industrial scale‐up, good manufacturing practice (GMP)‐compatible manufacturing, biocompatibility, and regulatory development [25]. The field therefore requires a paradigm shift from cataloguing nanomaterials toward a unified, pathology‐driven framework that connects mechanistic discovery with functional design, material selection, and clinical translation.

To remedy these limitations, a three‐tier integrative framework spanning TBI, SCI, and PNI is proposed. Anchored in epidemiological and single‐cell transcriptomic evidence, shared secondary pathological modules are compared across the three injury types to delineate temporal dynamics, principal cellular effectors, and injury‐specific bottlenecks, thereby defining what should be targeted. Comparative evaluation of nanomaterial platforms then interrogates how size, morphology, surface charge, protein corona formation, degradation kinetics, and mechanical, electrical, or magnetic properties govern biological fate and injury‐specific suitability, defining which platform to select. A Six Functional Paradigms taxonomy bridges pathological demand and material selection, encompassing scaffold construction and microenvironment remodeling, topographical guidance and contact‐mediated regeneration, biomimetic camouflage and active targeted delivery, endogenous microenvironment‐responsive release, exogenous physical‐field‐responsive intervention, and intracellular signaling modulation with cell‐fate reprogramming. This taxonomy defines the therapeutic function each material must perform. The evolutionary trajectory of neural nanomedicine is traced from passive encapsulation and active targeting to intelligent responsiveness and, ultimately, multi‐omics‐ and artificial intelligence (AI)‐driven personalized systems, while translational barriers, including scalable manufacturing, critical quality attributes, organoid or organ‐on‐chip bridging, large‐animal validation, patient stratification, and regulatory frameworks, are critically assessed. By integrating pathological mechanisms, functional strategies, and material platforms into a unified causal design chain, this review reframes traumatic neural injury nanomedicine from a material‐driven paradigm toward a pathology‐demand‐driven, function‐guided model, and provides an integrated roadmap for cross‐injury technology transfer and stratified, personalized neural restoration.

## 2 Epidemiological Scale and Cellular Pathological Architecture of Traumatic Neural Injuries

TBI, SCI, and PNI respectively afflict the brain, spinal cord, and peripheral nerves, three compartments distinguished by pronounced divergence in cytoarchitecture, barrier integrity, and intrinsic regenerative competence. Notwithstanding these distinctions, all three insults originate from acute mechanical trauma and subsequently unleash a cascade of vascular disruption, oxidative and metabolic perturbations, neuroinflammatory responses, ionic disequilibrium, diverse regulated cell death modalities, and remodeling of axons, myelin, and the extracellular matrix (ECM). Functional restoration does not merely hinge on the existence of a given pathological mechanism; rather, it depends on the identity of the dominant cellular executors, the patterns of their reciprocal interactions, and the constraints that render specific programs rate‐limiting within a defined anatomical niche [26]. This conceptual framework reconciles the conserved pathobiology of traumatic neural injury with the starkly divergent regenerative outcomes characteristic of the central nervous system (CNS) and peripheral nervous system (PNS) [27].

### 2.1 Global Disease Burden and Epidemiological Scale

Within the Global Burden of Disease (GBD) 2021 dataset analyzed here, age‐standardized disability‐adjusted life‐year (DALY) rates placed TBI and SCI 25th and 29th among neurological conditions, at 64.8 and 54.7 per 100 000 population, respectively (Figure 1A). Several highly prevalent neurological disorders, including stroke, neonatal encephalopathy, migraine, and Alzheimer's disease and other dementias, occupied higher positions. Population‐level rankings, however, do not directly reflect individual disease severity, because both TBI and SCI can produce abrupt and persistent neurological impairment requiring prolonged rehabilitation.

**FIGURE 1:**
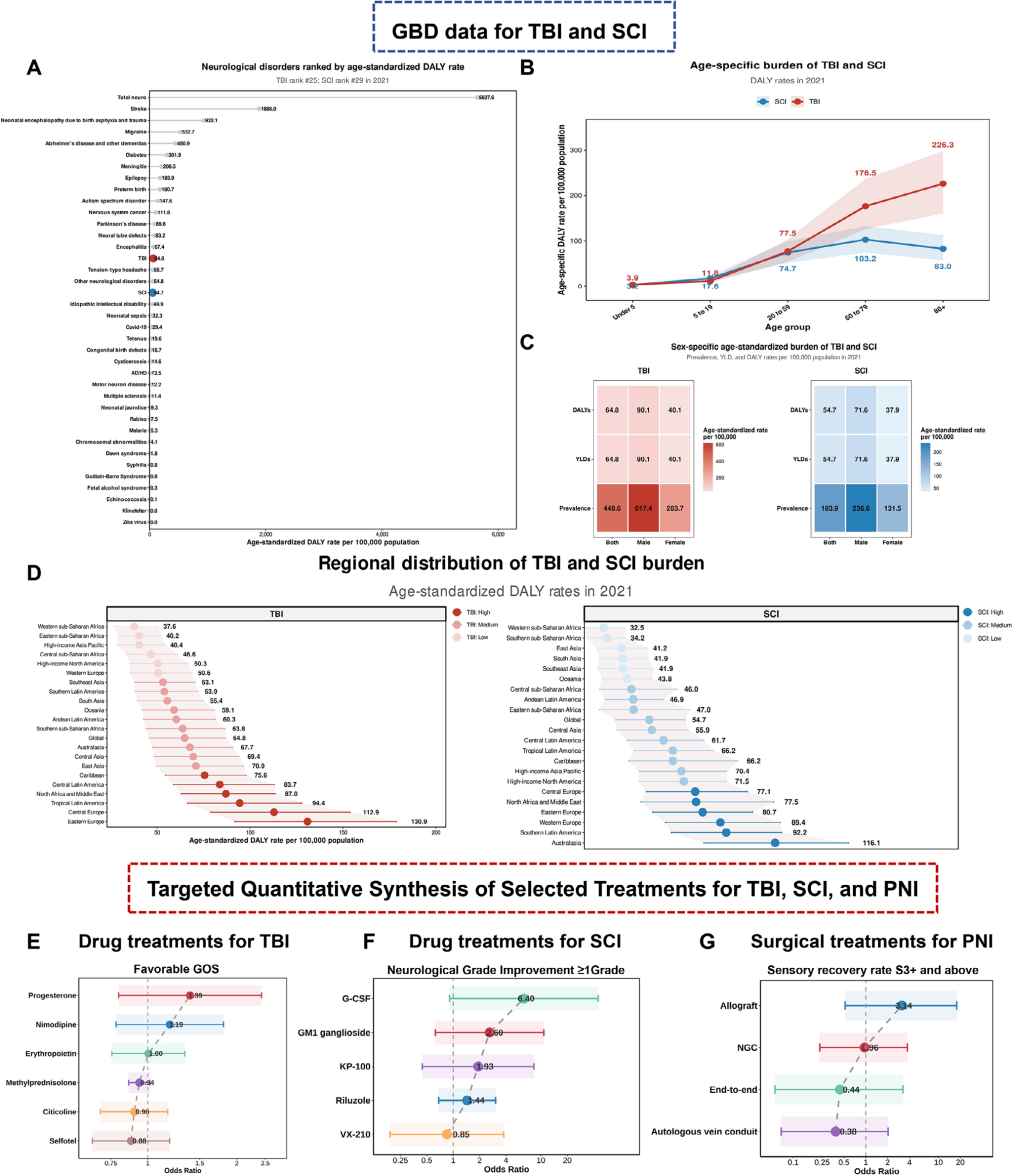
Epidemiological context of TBI and SCI based on GBD 2021 and targeted quantitative syntheses of selected clinical interventions for TBI, SCI, and PNI. (A) Neurological disorders ranked by age‐standardized DALY rates in 2021, with TBI and SCI highlighted. (B) Age‐specific DALY rates for TBI and SCI across all age groups in 2021. (C) Heatmap of the burden of TBI and SCI in 2021, stratified by sex and metrics (YLDs, prevalence, and DALYs). (D) Regional distribution of DALY rates for TBI and SCI in 2021. (E) Intervention‐versus‐control odds ratios for favorable GOS outcomes following selected pharmacological interventions for TBI. (F) Intervention‐versus‐control odds ratios for improvement of at least one neurological grade following selected pharmacological interventions for SCI. (G) Intervention‐versus‐control odds ratios for sensory recovery to S3+ or above following selected reconstructive strategies for PNI. Estimates for different interventions were derived from separate comparisons with their respective control groups and should not be interpreted as head‐to‐head comparisons or as establishing a treatment hierarchy.

Age‐stratified distributions delineated divergent DALY trajectories for TBI and SCI. TBI DALY rates ascended progressively from 3.9 per 100 000 among children younger than 5 years to 11.8 at 5 to 19 years, 77.5 at 20 to 59 years, 176.5 at 60 to 79 years, and 226.3 among individuals aged 80 years or older. SCI DALY rates, in contrast, displayed a distinct profile: 3.2 among children younger than 5 years, 17.6 at 5 to 19 years, and 74.7 at 20 to 59 years, before reaching 103.2 at 60 to 79 years and declining to 83.0 among individuals aged 80 years or older (Figure 1B). These patterns indicate that TBI burden accumulates particularly strongly in advanced age, whereas SCI burden increases through adulthood, peaks during early old age, and then declines modestly in the oldest population.

Sex‐specific age‐standardized estimates revealed a consistent male‐predominant burden. For TBI, prevalence reached 448.6 per 100 000 for both sexes, 617.4 in males, and 283.7 in females, while corresponding years lived with disability (YLD) and DALY rates were 64.8, 90.1, and 40.1. For SCI, prevalence reached 183.9 per 100 000 for both sexes, 236.6 in males, and 131.5 in females, with corresponding YLD and DALY rates of 54.7, 71.6, and 37.9 (Figure 1C). Regional distributions likewise proved heterogeneous. Eastern Europe recorded the highest age‐standardized TBI DALY rate at 130.9 per 100 000, followed by Central Europe at 112.9 and Tropical Latin America at 94.4. For SCI, Australasia showed the highest rate at 116.1 per 100 000, followed by Southern Latin America at 92.2 and Western Europe at 89.4. Western sub‐Saharan Africa exhibited the lowest regional rates for both TBI and SCI, at 37.6 and 32.5 per 100 000, respectively (Figure 1D). PNI does not appear separately within this GBD neurological ranking, and directly comparable global DALY estimates consequently remain unavailable. Epidemiological evidence for PNI derives predominantly from regional trauma registries. In a large United States claims‐based study encompassing approximately 16 million insured individuals, symptomatic PNI was diagnosed within 90 days in 1.64% of patients sustaining extremity trauma [28]. The data source, extraction dimensions, age‐group aggregation, rate transformation, and descriptive analytical procedures underlying Figure 1A–D are detailed in Section S1.

### 2.2 Current Clinical Management and Major Therapeutic Limitations

Current clinical management of traumatic neural injuries centers on physiological stabilization, prevention of additional tissue damage, surgical intervention for compressive lesions or disrupted nerve continuity when indicated, and subsequent rehabilitation. Such measures remain clinically indispensable, yet their capacity to directly modify the evolving secondary injury cascade or consistently restore lost neurological function proves limited. To place this therapeutic gap in quantitative clinical context, we performed a targeted synthesis of selected pharmacological interventions for TBI and SCI alongside reconstructive strategies for PNI. Tables 1, 2, 3 summarize the design, patient population, intervention and comparator, event counts, outcome definition, and follow‐up period of the included studies, while Figure 1E–G presents the corresponding effect estimates. Section S2 details the complete search strategy, eligibility criteria, study‐selection procedure, outcome harmonization, and effect‐estimate calculations, and Figure S1 depicts the study‐selection process.

**TABLE 1:** Characteristics of studies included in the targeted quantitative synthesis for TBI.

Study ID	Study design	Population / injury criteria	Experimental intervention	Events / N	Control intervention	Events / N	Outcome used in analysis	Follow‐up
Bailey 1991 [264]	Prospective randomized double‐blind placebo‐controlled trial	Severe head injury; not obeying commands; enrolled within 24 h	Nimodipine, IV 2 mg/h up to 7 days	93/175	Placebo	86/176	Favorable GOS (moderate disability or good recovery)	6 months
Xiao 2008 [265]	Prospective randomized placebo‐controlled trial	Acute severe TBI; GCS ≤8; treatment within 8 h	Progesterone, IM 1.0 mg/kg every 12 h for 5 days	48/82	Placebo	33/77	Favorable GOS	6 months
Aminmansour 2012 [266]	Randomized three‐arm clinical trial	Severe TBI; GCS ≤8	Progesterone, IM 1 mg/kg every 12 h for 5 days	9/20	Placebo	5/20	Favorable GOS / recovery	3 months
Skolnick 2014 [267]	Multinational prospective double‐blind randomized phase III placebo‐controlled trial (SYNAPSE)	Severe TBI; GCS ≤8; at least one reactive pupil; treatment within 8 h	IV progesterone for 120 h	298/591	Placebo	297/588	Favorable GOS (good recovery or moderate disability)	6 months
Robertson 2014 [268]	Randomized factorial clinical trial	Closed TBI unable to follow commands; enrolled within 6 h	Erythropoietin (EPO)	34/92	Placebo	34/89	Favorable GOS	6 months
Nichol 2015 [269]	Multicenter double‐blind randomized placebo‐controlled trial (EPO‐TBI)	Moderate or severe TBI; randomized within 24 h	Erythropoietin (EPO)	168/302	Placebo	162/294	Favorable GOSE (GOSE 5–8)	6 months
Zafonte 2012 [270]	Phase III double‐blind randomized placebo‐controlled trial (COBRIT)	Complicated mild, moderate, or severe TBI	Citicoline 2000 mg/day for 90 days	194/448	Placebo	209/455	Favorable GOSE	180 days
Morris 1999 [43]	Two parallel multicenter double‐blind randomized placebo‐controlled phase III trials	Severe TBI; post‐resuscitation GCS 4–8	Selfotel (CGS 19755), IV 5 mg/kg daily for 4 days	185/338	Placebo	204/352	Favorable GOS (good recovery or moderate disability)	6 months
Edwards 2005 [271]	Large multicenter randomized placebo‐controlled trial (MRC CRASH), 6‐month follow‐up report	Clinically significant head injury; GCS ≤14 within 8 h	IV methylprednisolone	2972/4854	Placebo	3026/4819	Favorable 6‐month functional outcome	6 months

*Abbreviations*: EPO, erythropoietin; GCS, Glasgow Coma Scale; GOS, Glasgow Outcome Scale; GOSE, Glasgow Outcome Scale–Extended;TBI, traumatic brain injury.

**TABLE 2:** Characteristics of studies included in the targeted quantitative synthesis for SCI.

Study ID	Study design	Population / injury criteria	Experimental intervention	Events / N	Control intervention	Events / N	Outcome used in analysis	Follow‐up
Geisler 1991 [272]	Prospective randomized double‐blind placebo‐controlled trial	Acute traumatic SCI; GM1 started within 72 h	GM1 ganglioside, 100 mg IV/day	8/16	Placebo	5/18	Neurological Grade Improvement ≥1 grade	1 year
Fehlings 2023 [273]	Multicenter randomized double‐blind placebo‐controlled trial (RISCIS)	Acute cervical SCI; AIS A–C; treatment within 12 h	Riluzole	48/65	Placebo	45/68	Neurological Grade Improvement ≥1 grade	180 days
Koda 2021 [274]	Randomized double‐blind placebo‐controlled phase III trial (G‐SPIRIT)	Acute traumatic cervical SCI	G‐CSF	33/36	Placebo	33/38	Neurological Grade Improvement ≥1 grade	6 months
Derakhshanrad 2019 [275]	Double‐blind randomized placebo‐controlled clinical trial	Subacute incomplete traumatic SCI	Subcutaneous G‐CSF	11/28	Placebo	0/26	Neurological Grade Improvement ≥1 grade	6 months
Derakhshanrad 2018 [276]	Double‐blind randomized placebo‐controlled phase III trial	Chronic incomplete traumatic SCI after rehabilitation	Subcutaneous G‐CSF	5/56	Placebo	0/58	Neurological Grade Improvement ≥1 grade	6 months
Nagoshi 2020 [31]	Multicenter randomized double‐blind placebo‐controlled phase I/II trial	Acute SCI; modified Frankel A/B1/B2 at 72 h	Intrathecal recombinant human HGF (KP‐100)	7/15	Placebo	5/16	Neurological Grade Improvement ≥1 grade	168 days
Fehlings 2021 [32]	Randomized double‐blind placebo‐controlled phase IIb/III trial (SPRING)	Acute traumatic cervical SCI; AIS A/B; decompression/stabilization within 72 h	Topical VX‐210 (Rho inhibitor), 9 mg	4/15	Placebo in fibrin sealant	6/20	Neurological grade improvement ≥1 grade	6 months

*Abbreviations*: AIS, American Spinal Injury Association Impairment Scale; G‐CSF, granulocyte colony‐stimulating factor; HGF, hepatocyte growth factor.;SCI, spinal cord injury.

**TABLE 3:** Characteristics of studies included in the targeted quantitative synthesis for PNI.

Study ID	Study design	Population / injury criteria	Experimental intervention	Events / N	Control intervention	Events / N	Outcome used in analysis	Follow‐up
Saeki 2018 [277]	Multicenter controlled open‐label clinical trial	Sensory nerve defect ≤30 mm at wrist or distal	Collagen nerve conduit	36/48	Autologous nerve graft	28/38	Sensory function recovery	12 months
Isaacs 2023 [278]	Multicenter prospective randomized blinded comparison (RECON)	Digital nerve injuries; long‐gap subgroup 15–25 mm	Collagen nerve conduit	13/20	Processed/decellularized nerve allograft	16/23	S3+ / favorable sensory recovery	≥6 to ≤15 months
Weber 2000 [279]	Randomized prospective multicenter trial	Digital nerve transections; graft‐comparator subgroup with gap ≥8 mm	Polyglycolic acid nerve conduit	12/17	Nerve graft (autograft)	6/8	Favorable sensory recovery	Up to 12 months
Rbia 2019 [280]	Retrospective single‐institution comparative case series	Digital nerve gaps; source file labels gap ∼16 mm	Collagen nerve conduit	14/19	Processed nerve allograft	17/18	Good/excellent sensory recovery	≥12 months
Leversedge 2020 [281]	Multicenter matched cohort study	Digital nerve gaps up to 25 mm	Nerve conduit	30/49	Processed nerve allograft	99/113	Meaningful sensory recovery (MRCC ≥S3)	Outcome‐assessment follow‐up
Agosti 2025 [282]	Systematic review (pooled secondary data; not an independent primary trial)	Upper‐extremity peripheral nerve injuries; source file labels 0–10 mm	Connector‐assisted repair / conduit	234/338	Direct repair	224/441	Higher‐threshold sensory recovery (MRCC ≥S3+)	Mean ∼24–26 months
Roganovic 2007 [283]	Prospective comparative study	Missile‐induced radial nerve defects; gap <6 cm	Autologous nerve graft	21/23	Autologous denatured muscle graft	7/9	Useful motor recovery (BMRC ≥M3)	≥4.7 years
Laveaux 2010 [284]	Retrospective monocentric comparative study with blinded review	Palmar digital nerve defects; defects ≤30 mm	Autologous nerve graft	12/15	Vein graft / conduit	11/17	Study‐defined favorable sensory result	≥11 months

*Abbreviations*: BMRC, British Medical Research Council; MRCC, Medical Research Council Classification;PNI, peripheral nerve injury.

Table 1 summarizes clinical studies for TBI evaluating progesterone, nimodipine, erythropoietin, methylprednisolone, citicoline, and selfotel. Progesterone yielded the largest point estimate for a favorable Glasgow Outcome Scale (GOS) outcome (odds ratio (OR) = 1.39), followed by nimodipine (OR = 1.19), whereas erythropoietin approximated the null (OR = 1.00). Methylprednisolone, citicoline, and selfotel produced corresponding estimates of 0.94, 0.90, and 0.88, respectively (Figure 1E). All displayed 95% confidence intervals encompassed unity, furnishing no statistically significant evidence of improved favorable GOS outcomes in this synthesis. The larger point estimate for progesterone therefore represents a numerical pattern within the available evidence rather than superiority over nimodipine or other interventions. This uncertainty converges with evidence from larger clinical trials: ProTECT III demonstrated no significant functional benefit of intravenous progesterone following TBI, and systematic evidence for citicoline likewise failed to show a significant improvement in favorable GOS [29, 30]. Numerical disparities among drug‐specific effect estimates should accordingly not be construed as an established efficacy hierarchy.

Table 2 compiles the SCI evidence for granulocyte colony‐stimulating factor (G‐CSF), GM1 ganglioside, KP‐100, riluzole, and VX‐210. G‐CSF produced the largest point estimate for improvement by at least one neurological grade (OR = 6.40), followed by GM1 ganglioside (OR = 2.60), KP‐100 (OR = 1.93), and riluzole (OR = 1.44), whereas VX‐210 fell below unity (OR = 0.85) (Figure 1F). The confidence interval for G‐CSF was notably wide, and the 95% confidence intervals for all five interventions encompassed unity. These findings denote considerable uncertainty and establish no statistically supported efficacy hierarchy among the evaluated agents. This conclusion aligns with broader clinical evidence: the randomized phase I/II KP‐100 study suggested signals of motor improvement, though most efficacy comparisons fell short of the conventional p < 0.05 threshold [31]. Similarly, the phase 2b/3 VX‐210 trial was terminated for futility and failed to achieve a statistically significant benefit on its primary efficacy endpoint [32].

Table 3 compiles comparative clinical evidence for nerve conduits, processed nerve allografts, autologous nerve grafts, direct repair, connector‐assisted repair, and vein‐based reconstruction in PNI. The estimates in Figure 1G derive from separate pairwise contrasts rather than comparisons against a shared reference treatment. These contrasts paired processed nerve allograft with nerve conduit repair, nerve guidance conduit with autologous nerve grafting, direct end‐to‐end repair with connector‐assisted repair, and autologous vein conduit with autologous nerve grafting. The processed allograft and conduit contrast yielded an OR of 3.14, whereas estimates for nerve guidance conduit, direct end‐to‐end repair, and autologous vein conduit reached 0.96, 0.44, and 0.38, respectively. Every confidence interval included unity. Because the studies differed in comparator, injury characteristics, gap length, outcome definition, and follow‐up period, these OR magnitudes do not share a common efficacy scale. Figure 1G therefore summarizes the direction and uncertainty of individual treatment contrasts without supporting direct ranking or statistical superiority among reconstructive strategies.

Collectively, although existing pharmacological and surgical interventions may address discrete clinical challenges such as intracranial hypertension, persistent spinal cord compression, or disruption of peripheral nerve continuity, their efficacy is frequently constrained by the evolving secondary injury cascade. A single pharmacological target or restoration of anatomical continuity alone is unlikely to capture the highly dynamic, cell‐type‐specific biology of post‐traumatic injury. This provides a biological basis for the numerical differences in treatment effects depicted in Figure 1E–G and helps explain the consistent absence of significant functional benefit across current interventions. A mechanistic understanding of these therapeutic limitations thus necessitates moving beyond tissue‐level clinical outcomes to delineate the cellular populations that execute major pathological programs, the pathways shared across injury types, and the processes that ultimately become rate‐limiting for recovery.

### 2.3 Single‐Cell Transcriptomic Characterization: Convergent and Divergent Pathobiological Responses

Separate reanalysis of publicly accessible single‐cell transcriptomic datasets for TBI [33], SCI [34], and PNI [35] examined injury‐associated alterations in cellular composition and transcriptional programs. All three datasets originated from mice, yet independent generation introduced differences in tissue source, injury paradigm, sampling schedule, dissociation and enrichment procedures, sequencing chemistry and coverage, cell recovery, and biological‐replicate structure. These disparities precluded integration into a common quantitative analysis. With dataset‐specific quality control completed, Harmony‐derived embeddings supported within‐dataset visualization and major‐cell‐class clustering (Section S3; Table S1 and Figures S2–S4). Harmony was applied separately within each dataset and cannot eliminate the biological and technical differences among the source studies.

Descriptive cell composition summaries revealed substantial remodeling after injury within each dataset (Figure 2A–C). In the TBI dataset, injured cortical samples exhibited greater relative abundance of microglia and macrophages, alongside diminished oligodendrocyte representation relative to sham. SCI dataset showed expanded monocyte, macrophage, neutrophil, and proliferative myeloid populations after injury, with reduced endothelial and ependymal representation relative to uninjured tissue. PNI dataset displayed pronounced macrophage enrichment after injury, accompanied by alterations across fibroblast, endothelial, neutrophil, lymphoid, mast cell, dendritic cell, and Schwann cell compartments. Collectively, these within‐dataset patterns substantiate recurrent engagement of immune and myeloid populations after neural trauma. Their apparent magnitude, however, does not permit direct comparison across injury types, because experimental design, tissue processing, enrichment strategy, and cell recovery may also shape these values.

**FIGURE 2:**
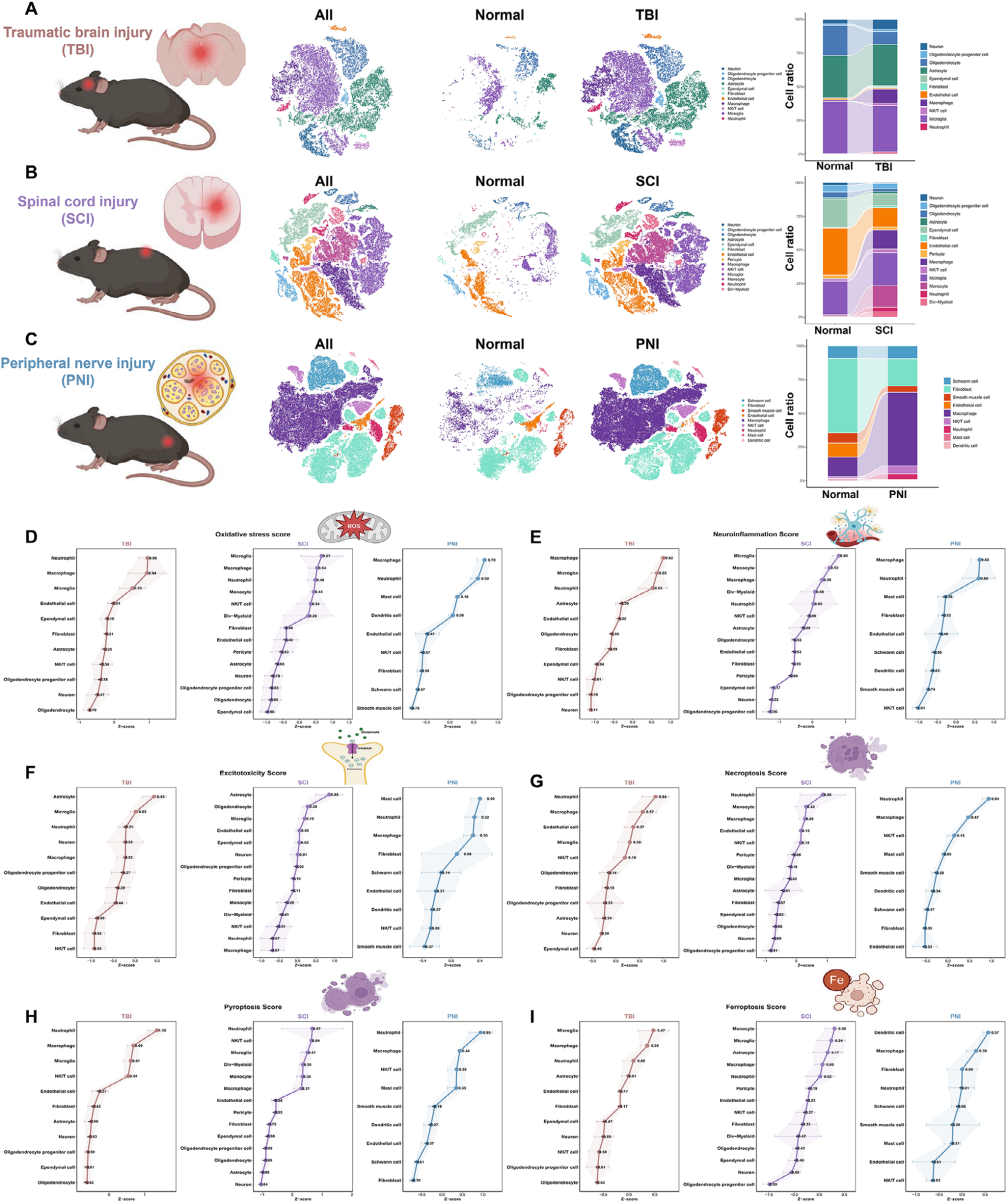
Single‐cell transcriptomic characterization: convergent and divergent pathobiological responses. (A‐C) Schematic diagrams of animal models and t‐SNE visualizations of single‐cell clustering (showing all cells, normal group, and injury group) alongside bar plots depicting proportional changes in cell populations between normal and injured conditions, presented for (A) TBI, (B) SCI, and (C) PNI. (D‐I) Cell‐type‐specific Z‐score distributions of six injury‐related signatures across TBI, SCI, and PNI: (D) oxidative stress score, (E) neuroinflammation score, (F) excitotoxicity score, (G) necroptosis score, (H) pyroptosis score, and (I) ferroptosis score. Selected schematic elements were created with BioRender.com.

Gene‐set curation captured the transcriptional signatures of each pathological module. Oxidative stress, neuroinflammation, excitotoxicity, necroptosis, and pyroptosis gene sets were derived through prespecified GeneCards keyword searches, with the top 50 ranked genes selected for each module; the ferroptosis gene set was obtained independently from the Driver gene list in FerrDB. Section S4 details gene‐selection criteria and module‐scoring procedures, and Table S2 provides the complete gene lists. Module scores were calculated separately for each dataset and standardized within that dataset for visualization. The resulting distributions described the relative expression of each gene program among annotated cell populations within the same injury dataset (Figure 2D–I). These scores do not constitute direct measurements of pathway activity and do not permit formal comparison of score magnitude among TBI, SCI, and PNI. No inferential testing was performed at either the individual‐cell or biological‐replicate level to evaluate cross‐injury differences in module scores.

These pathological modules operate as a dynamically coupled secondary injury network rather than as independent entities. Mechanical and vascular disruption triggers ionic disequilibrium, adenosine triphosphate (ATP) depletion, and mitochondrial dysfunction, thereby driving excessive ROS generation and excitotoxicity. Oxidative and metabolic disturbances further engage resident and infiltrating immune cells, and inflammatory mediators reciprocally intensify mitochondrial dysfunction and oxidative stress [36, 37]. Such interconnected disturbances activate multiple regulated cell death programs, encompassing ferroptosis, necroptosis, and pyroptosis, and ultimately culminate in neuronal and glial loss, axonal degeneration, demyelination, and subsequent extracellular matrix and scar remodeling. Although discussed separately below for analytical clarity, these processes interact reciprocally, and their injury‐specific temporal dominance collectively shapes the therapeutic bottlenecks of TBI, SCI, and PNI.

#### 2.3.1 Oxidative Stress Activation

Oxidative stress represents one of the earliest shared pathological events and functions as a principal amplifier of secondary injury. Mechanically induced membrane disruption, regional ischemia or hypoxia, and ATP depletion impair ion‐pump function, driving intracellular Na^+^ and Ca^2+^ accumulation. Mitochondrial Ca^2+^ overload subsequently disrupts electron transport and membrane potential, suppresses oxidative phosphorylation, and amplifies ROS generation. ATP deficiency further compromises Na^+^/K^+^‐ATPase and Ca^2+^ pump activity, establishing a self‐perpetuating loop of energy failure, Ca^2+^ overload, mitochondrial dysfunction, and ROS production. These interdependent events have been conclusively documented following SCI [36].

Mitochondria also link oxidative stress to innate immunity. Damaged mitochondria release mitochondrial DNA (mtDNA), ATP, and other damage‐associated molecular patterns (DAMPs), while mitochondrial bioenergetic failure, ROS accumulation, neuroinflammation, and blood‐brain barrier (BBB) compromise engage in extensive crosstalk after TBI [37]. Activated microglia and recruited neutrophils and monocytes/macrophages further amplify ROS burden via oxidative bursts and metabolic reprogramming.

Aligned with this mechanistic model, oxidative‐stress‐associated transcriptional programs are preferentially enriched in neutrophils, macrophages, and microglia in TBI; in microglia and multiple myeloid populations in SCI; and predominantly in macrophages and neutrophils in PNI. Despite divergent cellular compositions across these injuries, oxidative‐stress programs consistently converge on myeloid compartments, indicating that myeloid‐associated redox stress constitutes a conserved feature across anatomically distinct neural insults (Figure 2D). Single‐cell studies in SCI reveal extensive injury‐associated myeloid heterogeneity [34], while the injured sciatic nerve atlas (iSNAT) in PNI identifies a shift from early glycolytic inflammatory monocyte/macrophage states toward oxidative‐phosphorylation‐associated resolving states [35].

Persistent ROS promote lipid, protein, and DNA oxidation and synergize with dysregulated iron metabolism to drive lipid peroxidation. In PNI models, mitochondrial respiratory dysfunction, lipid ROS accumulation, perturbed iron homeostasis, and diminished ferroptosis suppressor protein 1 (FSP1)‐mediated defenses have been causally associated with Schwann‐cell ferroptosis and compromised nerve regeneration [38].

#### 2.3.2 Neuroinflammation and Immune Cell Infiltration

Neuroinflammation displays pronounced context‐dependent duality. Early immune activation serves indispensable roles in clearing necrotic cells and myelin debris, thereby facilitating tissue restoration, whereas persistent or dysregulated inflammation emerges as a principal driver of secondary injury. In TBI and SCI, DAMPs and chemotactic signals released by injured neurons, glial cells, and vascular elements rapidly induce reactive microglial phenotypes, while concurrent BBB/blood–spinal cord barrier (BSCB) disruption permits infiltration of neutrophils and circulating monocytes. Activated myeloid compartments subsequently elaborate tumor necrosis factor‐*α* (TNF‐*α*), interleukin‐1*β* (IL‐1*β*), and interleukin‐6 (IL‐6), chemokines, proteases, and ROS, thereby perpetuating vascular and neural damage.

Transcriptional signatures of neuroinflammation exhibit a pronounced myeloid preponderance (Figure 2E), with macrophages, microglia, and neutrophils predominating in TBI, microglia and monocyte/macrophage populations in SCI, and macrophages and neutrophils in PNI. TBI single‐cell studies show inflammatory transcriptional activation in both astrocytes and microglia [39], while SCI single‐cell profiling identifies heterogeneous myeloid states participating in angiogenic, gliotic, and fibrotic interactions [34].

In PNI, early glycolytic and inflammatory monocyte/macrophage states progressively transition toward oxidative‐phosphorylation‐associated inflammation‐resolving macrophages [35]. This complexity argues against a rigid M1/M2 framework; macrophage activation is best viewed as a continuum shaped by cellular origin, stimulus, and tissue context [40].

Therapeutic approaches should therefore avoid indiscriminate suppression of myeloid cells. A more rational objective is to limit persistent tissue‐destructive inflammatory states while preserving or enhancing the functions necessary for debris clearance, inflammation resolution, vascular restoration, and axonal regeneration.

#### 2.3.3 Excitotoxicity and Ionic Imbalance

Excitotoxicity operates as a central mechanistic nexus that couples primary mechanical injury to secondary neuronal death. Membrane disruption, bioenergetic collapse, and impaired neuronal and glial glutamate transport converge to elevate extracellular glutamate, which in turn drives excessive N‐methyl‐D‐aspartate (NMDA) receptor and *α*‐amino‐3‐hydroxy‐5‐methyl‐4‐isoxazolepropionic acid (AMPA) receptor activation and precipitates substantial Na^+^ and Ca^2+^ influx [41]. The ensuing intracellular Ca^2+^ overload engages calpains, phospholipases, and nitric oxide (NO) synthase, leading to cytoskeletal and membrane phospholipid degradation. Simultaneous mitochondrial Ca^2+^ accumulation collapses the membrane potential, curtails ATP synthesis, and amplifies ROS production.

Nitric oxide additionally reacts with superoxide to form peroxynitrite, a potent oxidant that inflicts protein nitration, DNA damage, and further respiratory‐chain inhibition, thereby aligning excitotoxicity tightly with oxidative stress, mitochondrial collapse, and downstream cell‐death cascades.

Excitotoxicity‐associated transcriptional programs are most strongly enriched in astrocytes across both TBI and SCI, with the greatest signal evident in SCI (Figure 2F). This enrichment does not imply that astrocytes are the principal victims of canonical excitotoxic death; instead, it likely reflects their central role in preserving extracellular glutamate and ionic homeostasis. Astrocytes mediate much of the extracellular glutamate clearance and participate in K^+^ buffering, metabolic substrate delivery, and the glutamate‐glutamine cycle; impairment of these functions prolongs glutamate accumulation and increases Ca^2+^ loading in neighboring neurons. Direct mechanistic support comes from experimental restoration of astrocytic excitatory amino acid transporter 2 (EAAT2) expression after SCI, which lowers tissue glutamate levels and attenuates excitotoxic pathology [42]. Single‐cell profiling of TBI likewise reveals elevated neurotransmitter transport, secretion, and exocytosis programs in excitatory neurons, along with marked reactive transcriptional shifts in astrocytes [39].

In PNI, the highest excitotoxicity‐associated scores occur primarily in mast cells and myeloid populations (Figure 2F). Because canonical glutamate excitotoxicity specifically denotes glutamate‐receptor‐mediated Ca^2+^ toxicity within neuronal–glial networks, this finding demands careful interpretation. A more plausible account is that the excitotoxicity gene set also includes shared components of Ca^2+^ homeostasis, ion‐channel activity, oxidative stress, and injury‐response signaling that may be enriched in immune populations following PNI. This enrichment should not be interpreted as proof that these cells undergo classical NMDA‐dependent excitotoxicity.

Therapeutically, excitotoxicity is characterized by a narrow early intervention window and close coupling to metabolic failure and inflammation, which may partly explain the poor clinical translation of single‐target approaches such as systemic NMDA receptor antagonism [43]. Approaches that combine localized delivery with simultaneous restoration of glial glutamate handling, Ca^2+^ homeostasis, and mitochondrial function may therefore hold greater therapeutic promise.

#### 2.3.4 Interconnected Programs of Regulated Cell Death

Cell death serves as the direct conduit through which secondary injury culminates in irreversible neurological impairment. Beyond classical apoptosis and unregulated necrosis, necroptosis, pyroptosis, and ferroptosis have emerged as integral components of traumatic neural injury. Necroptosis proceeds via receptor‐interacting protein kinase 1 (RIPK1)‐receptor‐interacting protein kinase 3 (RIPK3)‐mixed lineage kinase domain‐like pseudokinase (MLKL) signaling; pyroptosis is canonically executed through inflammasome‐caspase‐gasdermin cascades; and ferroptosis arises from iron‐dependent phospholipid peroxidation that overwhelms endogenous antioxidant defenses, including the cystine/glutamate antiporter system Xc^−^‐reduced glutathione (GSH)‐glutathione peroxidase 4 (GPX4) axis and the FSP1‐coenzyme Q (CoQ) axis.

Transcriptional signatures associated with necroptosis display a pronounced neutrophil preponderance across all three injuries (Figure 2G), although such enrichment alone does not establish direct execution of necroptotic death. Independent experimental evidence nevertheless corroborates mechanistic involvement: neuronal deletion of RIPK1 or RIPK3 following TBI attenuates progressive neuronal loss and phosphorylated MLKL (pMLKL) accumulation while improving memory function [44].

Pyroptosis‐associated programs likewise preferentially engage innate immune populations (Figure 2H). Importantly, pathway activation need not proceed to complete cellular lysis. Schwann cells subjected to PNI can manifest sublytic pyroptosis, wherein caspase‐1‐associated inflammasome activation profoundly disrupts remyelination despite limited overt cell death [45]. Collectively, necroptosis‐ and pyroptosis‐associated programs exhibit a robust innate‐immune bias, particularly toward neutrophils and monocyte/macrophage compartments, indicating that lytic cell‐death signaling is intimately coupled to myeloid inflammatory amplification after injury (Figure 2G,H).

Ferroptosis‐associated signatures reveal more pronounced injury‐specific variation (Figure 2I), yet transcriptional ranking does not invariably identify all biologically vulnerable populations. Oligodendrocyte ferroptosis following SCI has been causally linked to diminished FSP1 activity and compromised lipid‐peroxidation defenses [46], whereas mitochondrial dysfunction and ferroptotic injury have been experimentally substantiated in Schwann cells after PNI [38].

Mitochondrial ROS, cytosolic DNA, dysregulated iron metabolism, and inflammatory mediators provide convergent upstream signals, while cyclic GMP‐AMP synthase‐stimulator of interferon genes (cGAS‐STING) signaling may intersect with autophagy, necroptosis, ferroptosis, and pyroptosis [47]. These pathways are therefore more accurately conceived as an interconnected, cell‐ and time‐dependent death network rather than discrete, parallel processes.

#### 2.3.5 Demyelination, Axonal Degeneration, and Divergent Regenerative Capacity

Demyelination and axonal degeneration constitute shared terminal pathways that culminate in conduction failure, yet the central nervous system (CNS) and peripheral nervous system (PNS) respond in fundamentally distinct ways. Oligodendrocytes and their progenitors exhibit pronounced vulnerability to mechanical trauma, hypoxia, oxidative stress, excitotoxicity, and inflammatory mediators following TBI and SCI. Experimental evidence of oligodendrocyte ferroptosis after SCI further implicates oxidative injury, regulated cell death, and myelin loss as interconnected pathological events [46].

CNS myelin also harbors regeneration‐suppressive signals. Nogo‐A and myelin‐associated glycoprotein (MAG) regulate RhoA/Rac1‐dependent cytoskeletal dynamics and induce growth‐cone collapse [48], Together with oligodendrocyte myelin glycoprotein (OMgp), these inhibitors can activate RhoA‐Rho‐associated coiled‐coil‐containing protein kinase (ROCK) signaling to restrict axonal regrowth [49].

In marked contrast, PNI initiates Wallerian degeneration. Distal axons and myelin are actively dismantled, and myelinating and nonmyelinating Schwann cells undergo profound transcriptional reprogramming into specialized repair phenotypes that support debris clearance, trophic signaling, immune recruitment, and the assembly of longitudinal Büngner bands for regenerating axons [50]. The extensive intercellular communication captured by iSNAT further emphasizes that peripheral nerve regeneration is an ecosystem‐level process rather than an axon‐autonomous event [35].

#### 2.3.6 Glial Scarring, Fibrotic Scarring, and Extracellular Matrix Remodeling

The post‐traumatic CNS scar constitutes a heterogeneous multicellular ensemble that encompasses an astrocytic border at the lesion perimeter, stromal and fibrotic populations within the lesion core, vascular‐associated elements, myeloid immune cells, and extensively remodeled extracellular matrix. Its functional consequences are phase‐dependent: early astrocytic borders restrict inflammatory spread and preserve tissue architecture, whereas persistent ECM deposition and regeneration‐inhibitory signals later constrain axonal extension. The glial scar therefore cannot be regarded as uniformly detrimental. Genetic ablation, attenuation, or removal of scar‐forming astrocytes failed to induce spontaneous axonal regrowth after severe SCI, and preventing scar formation reduced axonal regrowth even when exogenous growth‐promoting cues were supplied [51].

The fibrotic core exhibits comparable heterogeneity. Lineage tracing combined with single‐cell RNA sequencing in murine and nonhuman primate models identifies perivascular and meningeal fibroblasts, rather than canonical pericytes or vascular smooth muscle cells, as the principal scar‐forming populations, with distinct fibroblast subsets showing divergent spatial distributions and ECM functions [52]. These findings advocate spatiotemporally selective ECM modulation rather than indiscriminate scar removal. A recently engineered ROS‐responsive gene‐delivery platform targeting reactive astrocytes achieves localized degradation of chondroitin sulfate proteoglycans, interrupts maladaptive microglia‐astrocyte crosstalk, and improves functional restoration in experimental SCI [53].

### 2.4 Cross‐Injury Pathological Bottlenecks and Therapeutic Priorities

TBI, SCI, and PNI converge upon an integrated secondary‐injury network characterized by myeloid‐driven immune activation, oxidative and metabolic dysregulation, ionic imbalance, and diverse cellular damage cascades. Despite this convergence, the shared pathological modules are executed by distinct cellular effectors and governed by divergent anatomical constraints.

TBI is distinguished by the centrifugal spread of secondary pathology into peri‐lesional and remote white‐matter territories, accompanied by spatiotemporally heterogeneous BBB permeability [21]. Its principal therapeutic challenge therefore lies in achieving distributed delivery and multicellular neuroprotection under dynamically fluctuating barrier conditions.

SCI superimposes intense secondary injury upon frank physical discontinuity of the spinal cord. The confluence of myeloid inflammation, oxidative stress, astrocyte‐associated excitotoxicity, oligodendroglial vulnerability, demyelination, and subsequent fibrotic remodeling demands a biphasic intervention, encompassing early preservation of surviving tissue followed by structural bridging, remyelination, and electrophysiological restoration [22].

PNI, by contrast, couples substantial intrinsic regenerative capacity with stringent spatial and temporal constraints. Wallerian degeneration, macrophage‐mediated clearance, and the emergence of reparative Schwann cells are integral to regeneration, yet functional recovery hinges on timely and correctly directed axonal regrowth before irreversible target denervation [24].

Therapeutic design should therefore prioritize precise alignment with dominant cellular populations, pathological programs, therapeutic windows, and spatial constraints rather than the mere accumulation of material functions. These factors collectively define the rate‐limiting determinants of recovery in each injury context (Table 4).

**TABLE 4:** Cross‐injury pathobiological features, therapeutic bottlenecks, and nanomedicine design requirements in traumatic neural injuries.

Injury type	Anatomical and barrier context	Dominant cellular and pathological features	Regenerative profile	Rate‐limiting therapeutic bottleneck	Priority nanomedicine design requirements	Representative refs.
**TBI**	Focal and/or diffuse parenchymal injury; secondary pathology can propagate into peri‐lesional and remote white‐matter regions; BBB permeability is spatially and temporally heterogeneous	Predominant remodeling of microglia/macrophages with neutrophil involvement; oxidative and mitochondrial dysfunction, neuroinflammation, excitotoxicity, regulated cell death, oligodendroglial injury and demyelination	Limited intrinsic CNS regeneration; diffuse and heterogeneous injury complicates structural reconstruction	Achieving sufficiently distributed therapeutic exposure and multicellular neuroprotection despite dynamically evolving BBB permeability and pathological heterogeneity	BBB‐aware delivery; lesion‐ and cell‐selective targeting; ROS/inflammation‐responsive release; temporally programmed or sequential therapy; simultaneous modulation of neural and immune compartments	[33, 37, 39, 44, 152, 214, 215]
**SCI**	Disrupted BSCB superimposed on frank physical discontinuity; cystic cavitation and highly organized white‐matter tracts impose both biochemical and structural barriers	Strong myeloid response involving microglia, monocytes/macrophages and neutrophils; oxidative stress, excitotoxicity, oligodendrocyte injury/ferroptosis, demyelination, astroglial and fibrotic remodeling	Very limited spontaneous long‐distance CNS regeneration; inhibitory myelin/ECM cues and tissue discontinuity restrict axonal reconnection	Coordinating early tissue preservation with subsequent structural bridging, remyelination and circuit/electrophysiological reconstruction	Local retention and sustained release; antioxidant/immunomodulatory activity; injectable cavity‐filling matrices; anisotropic guidance; pro‐remyelinating and circuit‐restorative functions	[22, 34, 36, 42, 46, 48, 49, 51, 52, 153, 285]
**PNI**	Relatively permissive peripheral barrier environment, but nerve gaps impose strong geometric and temporal constraints on regeneration	Macrophage‐dominated immune remodeling; Wallerian degeneration; repair‐Schwann‐cell activation; oxidative/ferroptotic stress; angiogenic and ECM remodeling	Substantially greater intrinsic regenerative capacity than the CNS; repair Schwann cells and Büngner bands provide regenerative support	Regenerating axons must traverse sufficient distances along appropriate trajectories and reinnervate distal targets before irreversible target denervation	Mechanically competent nerve conduits; longitudinal/topographical guidance; sustained trophic support; Schwann‐cell modulation; angiogenic support; degradation synchronized with axonal regeneration	[24, 35, 38, 45, 50, 154]

## 3 Nanomaterial Classification, Physicochemical Properties, and Injury‐Specific Adaptations

A structured framework for nanoparticle selection in neural restoration is presented, organized across three interconnected layers. The initial layer establishes a comprehensive taxonomy of nanoparticle platforms, each interrogated for physicochemical underpinnings, therapeutic advantages, translational constraints, and injury‐specific suitability. Subsequent attention turns from particle identity to particle properties, examining how size, geometry, surface charge, protein corona formation, and biodegradation kinetics govern nano‐bio interactions and dictate therapeutic outcomes [54]. These insights are distilled into injury‐specific trade‐off matrices, yielding a demand‐driven selection logic that aligns candidate nanoparticles with the distinct pathological requirements of TBI, SCI, and PNI across six evaluative dimensions: biological barrier penetration, degradation product safety, functional compatibility, drug‐loading capacity, scalability, and immunogenicity. This tripartite progression from particle taxonomy through physicochemical principles to injury‐specific prioritization lays the foundation for the functional paradigms detailed in the subsequent chapter.

### 3.1 Classification and Cross‐Platform Comparison of Nanoparticle Systems

Nanomaterials engineered for neural restoration constitute a heterogeneous array of platforms distinguished by their structural, physicochemical, and biological signatures. These systems are broadly classified into lipid‐based, polymeric, inorganic, carbon‐based, biomimetic, extracellular vesicle‐derived, and hybrid nanoparticle‐biomaterial constructs. Divergence in cargo capacity, biointerfacial behavior, biodegradability, immunogenicity, and intrinsic functionality governs their appropriateness for specific neural injury contexts. The ensuing sections outline the principal characteristics, therapeutic advantages, and translational limitations of these major platforms.

Collectively, these nanomaterial classes differ in intrinsic physicochemical properties and in cargo compatibility, biological interactions, manufacturability, and injury‐specific utility. No single platform achieves universal optimality across TBI, SCI, and PNI. Material selection instead demands a balance between therapeutic function and biological and translational constraints. Table 5 accordingly summarizes the principal advantages, limitations, and injury‐specific applicability of these platforms.

**TABLE 5:** Comparative characteristics, advantages, limitations, and injury‐specific applicability of major nanomaterial platforms for traumatic neural repair.

Material class	Representative materials / key features	Major advantages	Major limitations	Injury‐specific applicability	Representative refs.
**Lipid‐based nanoparticles**	Liposomes, SLNs, NLCs, lipid nanoemulsions, and cationic lipid complexes; membrane‐like self‐assembled structures; compatible with hydrophilic and hydrophobic cargos	High biocompatibility; versatile cargo encapsulation; surface PEGylation and ligand modification; membrane fusion and efficient cellular uptake; comparatively mature manufacturing and regulatory experience	Limited loading for some cargos; fusion, leakage, and lipid degradation during storage/circulation; repeated PEGylated formulations may induce anti‐PEG antibodies and accelerated blood clearance	**TBI**: particularly suitable for systemic delivery and BBB‐oriented targeting. **SCI**: useful for BSCB penetration and lesion‐directed drug delivery. **PNI**: mainly adjunctive/local delivery; insufficient alone to provide structural guidance across nerve gaps	[55, 56, 57, 58, 59, 60, 61, 62, 63, 64, 65]
**Polymeric nanoparticles**	PLGA, PLA, PCL, chitosan, gelatin, and related copolymers; highly tunable size, surface chemistry, degradation, and release kinetics	Excellent structural tunability; programmable sustained release; broad opportunities for ligand modification; biodegradable formulations; established medical use of several constituent polymers	Acidic degradation products may compromise sensitive cargos; residual organic solvents and polymer polydispersity complicate quality control; relatively inefficient encapsulation of some hydrophilic biomacromolecules	**TBI**: prolonged drug/gene delivery when brain accumulation is appropriately engineered. **SCI**: sustained anti‐inflammatory, antioxidant, or regenerative delivery. **PNI**: particularly compatible with prolonged local delivery and integration into nerve‐repair constructs	[67, 68, 69, 70, 71, 72, 73, 74, 75]
**Inorganic nanoparticles**	AuNPs, Fe_3_O_4_/γ‐Fe_2_O_3_ nanoparticles, MSNs, calcium‐based nanomaterials, UCNPs; intrinsic optical, magnetic, catalytic, or ion‐releasing properties	Unique physical functionality; magnetic guidance; photothermal/optical conversion; high surface area and loading capacity of porous materials; imaging/theranostic capability; externally triggered intervention	Many formulations are slowly degradable or non‐biodegradable; potential long‐term tissue/organ retention; size‐ and charge‐dependent off‐target accumulation; clearance and chronic neurotoxicity require careful evaluation	**TBI**: selected applications in imaging, ROS regulation, or externally triggered intervention. **SCI**: particularly attractive for magnetic, optical, catalytic, and physical‐field‐responsive therapy. **PNI**: useful for localized magnetic guidance, oxygen/ion delivery, imaging, and functionalized repair constructs	[76, 77, 78, 79, 80, 81, 82, 83]
**Carbon‐based and conductive nanomaterials**	CNTs, graphene/graphene oxide, carbon‐based conductive components and related electroactive nanostructures; high electrical conductivity and mechanical reinforcement	Electrical signal transmission; tunable conductivity; mechanical reinforcement; supports electroactive scaffolds, nerve conduits, and neural interfaces	Cytotoxicity depends strongly on size, aspect ratio, aggregation, and surface chemistry; CNTs may provoke persistent inflammatory/fibrotic responses; uncertain long‐term metabolism and clearance; scalability and regulatory standardization remain challenging	**TBI**: limited as systemic carriers; selected local neural‐interface applications. **SCI**: useful in conductive scaffolds and electroactive repair systems. **PNI**: particularly suitable for conductive nerve guidance conduits and topographical/electrical stimulation	[84, 85, 86, 87, 88, 89, 90, 91, 92]
**Biomimetic nanoparticles and extracellular vesicles**	Cell‐membrane‐coated nanoparticles and EVs/exosomes; inherit membrane proteins, receptors, or endogenous biological cargo from source cells	Immune camouflage; inflammatory or cell‐specific homing; favorable biointerfacial recognition; potential barrier traversal; EVs provide intrinsic multi‐molecular bioactivity	EV yield and scalable production remain limited; source‐ and batch‐dependent heterogeneity; difficult standardization of cargo and potency; isolation/purification can affect vesicle integrity; GMP manufacturing remains challenging	**TBI**: highly attractive for BBB traversal, inflammatory homing, and cell‐selective delivery. **SCI**: useful for inflammatory targeting and immunomodulation. **PNI**: promising for Schwann‐cell/axon modulation and regenerative signaling, especially through local delivery	[93, 94, 95, 96, 97, 98, 99, 106, 107, 108]
**Hybrid nanoparticle–biomaterial systems**	Nanocomposite hydrogels, electrospun nanofiber composites, conductive nanocomposite hydrogels, MOF‐containing constructs, and 3D‐printed nanobioinks; combine nanoscale functionality with macroscopic structural support	Integrate structural filling/guidance with sustained delivery, microenvironment regulation, and physical signal transduction; tunable mechanics; localized retention; capable of multifunctional and spatiotemporal intervention	Increasing component number raises manufacturing and characterization complexity; batch reproducibility, sterilization, storage stability, scalability, and regulatory classification become more difficult; excessive multifunctionality may not translate into proportional therapeutic benefit	**TBI**: most appropriate for focal cavities or injectable/local constructs rather than diffuse injury. **SCI**: highly suitable for cavity filling plus sustained and responsive therapy. **PNI**: highly suitable for conduits/scaffolds requiring simultaneous structural guidance and biochemical/electrical cues	[109, 111, 112, 113, 114, 115, 116, 117, 118, 119]

#### 3.1.1 Lipid‐Based Nanoparticles

Lipid‐based nanoparticles represent one of the most clinically mature nanomedicine platforms, comprising liposomes, solid lipid nanoparticles (SLN), nanostructured lipid carriers (NLC), lipid nanoemulsions, and cationic lipid complexes [55]. Assembled from phospholipids and cholesterol, their bilayer architecture closely mimics native cell membranes, conferring exceptional biocompatibility and minimal immunogenicity [56]. The compartmentalized design enables simultaneous loading of hydrophilic agents within the aqueous core and hydrophobic cargo within the lipid bilayer, affording versatile platforms for combination therapy [57]. Polyethylene glycol (PEG) functionalization prolongs systemic circulation, whereas conjugated ligands direct lesion‐specific accumulation [58]. Notably, apolipoprotein E (ApoE)‐mediated low‐density lipoprotein receptor (LDLR) transcytosis allows these nanoparticles to traverse the BBB [59]. Together with membrane fusogenicity and efficient cellular internalization, such features render lipid‐based carriers uniquely suited to systemic delivery in acute settings that require multi‐target engagement, extended circulation, and controlled biodistribution. Clinical translation stands unmatched, as evidenced by the regulatory success of Doxil, Onpattro, and Comirnaty, which have collectively validated industrial manufacturing pathways and mitigated regulatory risk for neurological indications [60, 61].

Within TBI, lesion‐targeted lipid‐coated nanocarriers co‐delivering nimodipine and ROS‐scavenging agents enhance drug accumulation at injury sites while attenuating BBB permeability, cerebral edema, neuroinflammation, and neuronal death [62]. In SCI, systemically administered basic fibroblast growth factor (bFGF)‐loaded liposomes penetrate the damaged spinal cord, elevate lesion drug exposure, restore tight junction‐associated barrier proteins, and promote angiogenesis and functional restoration [63]. In PNI, lipid‐based carriers serve primarily as adjuncts within nerve conduits or scaffolds for localized drug release, lacking the structural guidance required for long‐segment nerve defects.

Despite these advantages, translational challenges persist. Drug loading below 10% (w/w) in conventional liposomes limits high‐dose neurotrophic factor delivery [64]. Physical instability, including fusion, leakage, and lipid degradation, remains problematic during storage and circulation. Repeated administration of PEGylated liposomes induces anti‐PEG antibodies and triggers accelerated blood clearance (ABC), compromising subsequent therapeutic efficacy [65]. These limitations underscore that while lipid‐based nanocarriers offer unmatched clinical accessibility and versatility for neurological applications, their utility remains bounded by intrinsic physicochemical constraints and immunological hurdles that must be rigorously considered during platform selection.

#### 3.1.2 Polymeric Nanoparticles

Polymeric nanoparticles draw upon a versatile repertoire of natural and synthetic polymers, spanning poly(lactic‐co‐glycolic acid) (PLGA), poly(lactic acid) (PLA), chitosan, and gelatin [66]. Exceptional structural tunability ranks among their foremost advantages: diblock copolymer engineering affords precise control over particle dimensions, surface charge, amphiphilic balance, and ligand conjugation sites [67], thereby enabling systematic optimization of release kinetics and biodistribution. Degradation profiles range from days to years, with PLGA hydrolysis rates tuned through the lactic‐to‐glycolic acid ratio [68], aligning with distinct therapeutic windows: rapid anti‐inflammatory release for acute phases, and sustained neurotrophic delivery over weeks to months for chronic needs. PLGA, PLA, and poly(ε‐caprolactone) (PCL) have each secured U.S. Food and Drug Administration (FDA) approval for medical devices and drug delivery systems, establishing a strong regulatory foundation and clinical safety record [69].

Nevertheless, the limitations of polymeric nanoparticles warrant equally rigorous attention. Acidic degradation products from PLGA can destabilize, aggregate, or hydrolyze encapsulated proteins [70], making entrapment efficiency alone an insufficient index of therapeutic potential. Residual organic solvents such as dichloromethane and chloroform raise potential toxicity concerns. Polymerization‐induced polydispersity causes inter‐batch variability in molecular weight and release profiles, posing substantial quality‐control challenges. Hydrophilic biomacromolecules, especially proteins and nucleic acids, encapsulate with comparatively low efficiency [71]; N,N,N′,N′‐tetrakis(2‐pyridylmethyl)ethylenediamine (TPEN)‐loaded PLGA nanoparticles, for instance, display approximately 50% entrapment that falls to 28% with increasing aqueous‐phase fraction, reflecting thermodynamic incompatibility between hydrophobic matrices and hydrophilic cargo [72].

In terms of context‐specific adaptability, polymeric nanoparticles find primary application in sustained release and gene delivery. Galantamine‐loaded PLGA nanoparticles achieve marked motor improvement and lesion volume reduction in rat SCI models through anti‐inflammatory, antioxidant, and glial‐modulatory effects [73]. Continuous insulin‐like growth factor 1 (IGF‐1) delivery via nanoparticles attenuates denervation‐induced muscle atrophy, suppresses Schwann cell senescence, and enhances axonal regeneration and functional restoration [74]. For gene delivery, optimized small interfering RNA (siRNA)‐loaded PLGA nanoparticles maintain brain accumulation despite substantial BBB restoration, achieving roughly threefold higher deposition than conventional PEGylated particles and about 50% gene silencing [75]. Polymeric nanoparticles therefore excel in structural tunability and degradation control, making them well suited to neural restoration scenarios requiring prolonged or programmed drug exposure; platform selection, however, must carefully weigh acidic degradation microenvironments, organic solvent residues, batch consistency, and hydrophilic drug loading efficiency.

#### 3.1.3 Inorganic Nanoparticles

Inorganic nanoparticles constitute a diverse family of platforms, including gold nanoparticles (AuNPs), magnetic iron oxide nanoparticles (Fe_3_O_4_/γ‐Fe_2_O_3_), mesoporous silica nanoparticles (MSNs), calcium‐based nanomaterials such as calcium peroxide, and upconversion nanoparticles (UCNPs) [76]. These platforms exhibit distinctive optical, magnetic, and electrical properties, endowing them with unique utility in neural theranostics. By virtue of their tunable near‐infrared absorption, gold nanoparticles serve as efficient photothermal transducers; near‐infrared‐responsive microneedle patches incorporating gold@polyphenol nanospheres mitigate spinal cord injury pathology by modulating the mitochondrial ROS microenvironment, scavenging reactive species, and attenuating neuronal apoptosis [77]. Magnetic nanoparticles, exemplified by Fe_3_O_4_‐coated mesoporous silica, enable enhanced drug retention at lesion sites under external magnetic guidance. In a rat model of bilateral cavernous nerve injury, magnetic mesoporous silica nanoparticles carrying RADA16‐I/RAD‐RGI peptides and surface‐conjugated with a neural‐targeting peptide promoted nerve regeneration and restored erectile function [78]. Mesoporous silica nanoparticles possess highly ordered mesoporous architectures (2‐10 nm pore diameter), large surface areas, and readily modifiable surfaces, enabling efficient drug loading and controlled release [79]. Scaffolds loaded with calcium peroxide nanoparticles achieve sustained oxygen release over 15 days, mitigating early hypoxia and supporting metabolic demands before vascular reconstruction [80]. Upconversion nanoparticles transduce near‐infrared excitation into visible emission, overcoming the limited tissue penetration of conventional fluorophores, and an 808 nm‐excited core‐multishell design enabled stable single‐particle fluorescence tracking for over 30 min, offering a potent tool for long‐term intracellular tracing in neurons [81].

In terms of context‐specific utility, inorganic nanoparticles are best suited to externally triggered interventions and imaging‐guided therapy, as the aforementioned examples illustrate their potential in photothermal modulation, magnetic targeting, and fluorescence tracing. Nevertheless, clinical translation of inorganic nanomaterials confronts substantial obstacles. Most inorganic materials are either non‐biodegradable or degrade exceedingly slowly in vivo, with half‐lives spanning months to years, raising long‐term toxicity concerns [82]. The biodistribution of metallic nanoparticles varies substantially with particle size and surface charge, leading to significant accumulation in off‐target organs such as the liver and spleen [83], which necessitates rigorous investigation of metabolic pathways and clearance profiles. Consequently, non‐biodegradability and organ accumulation remain core bottlenecks limiting clinical application. Additional physical functionalities are justified only when they offer quantifiable advantages beyond those achievable with biodegradable carriers, rendering these materials more appropriate for localized interventions requiring magnetic responsiveness, electrical conductivity, or oxygen delivery rather than serving as primary systemic carriers.

#### 3.1.4 Carbon‐Based Nanoparticles

Carbon‐based nanoparticles comprise a diverse family of platforms, including single‐walled and multi‐walled carbon nanotubes (CNTs), single‐walled carbon nanotubes (SWCNTs) and multi‐walled carbon nanotubes (MWCNTs), graphene and its derivatives such as graphene oxide (GO) and reduced graphene oxide (rGO), carbon quantum dots (CQDs), and MXene transition metal carbides/nitrides. These materials confer pronounced advantages in conductive neural tissue engineering, attributable to superior electrical conductivity, mechanical robustness, and exceptionally high specific surface area [84].

CNTs, distinguished by high aspect ratios and conductivity, have been widely integrated into hydrogels to enhance mechanical and electrical performance; aligned CNTs within gelatin methacryloyl (GelMA) scaffolds markedly promoted PC12 cell growth, differentiation, and oriented neurite extension [85]. Graphene and its derivatives, characterized by two‐dimensional sheet architecture and expansive surface area, effectively support neuronal adhesion and synapse formation [86]. Magnetic graphene oxide (MGO)‐functionalized composite nanofiber scaffolds sustain neural stem cell adhesion and proliferation while directing differentiation toward excitatory neuronal phenotypes and suppressing excessive astrocytic activation [87]. Carbon quantum dots, valued for photostability, biocompatibility, and tunable emission, hold potential in neural imaging and sensing, although their direct application in nerve restoration remains relatively limited [88]. MXene, an emerging two‐dimensional conductive nanomaterial, has shown considerable promise in neural regeneration; MXene‐MnO_2_ composite hydrogel nerve conduits significantly enhanced axonal regeneration and vascularization through Schwann cell proliferation, ROS scavenging, and macrophage M2 polarization [89]. Silk fibroin‐MXene conductive conduits integrated with IGF‐1‐loaded hydrogels effectively promoted Schwann cell spatial distribution and oriented axonal regeneration in long‐segment PNI [90]. Notably, conductive substrates modified with CNTs and graphene oxide nanoribbons revealed that physiologically relevant conductivity (0.02‐0.1 S/m) more effectively promoted neural stem cell differentiation toward neuronal lineages than excessive conductivity (3.2 S/m), which instead triggered apoptosis via calcium overload [91]. Conductivity optimization should therefore follow physiological adaptation principles rather than pursuing maximal values.

Clinical translation of carbon‐based nanoparticles confronts multiple challenges. Cytotoxicity of CNTs and graphene remains debated, with inconsistent findings attributable to variations in size, length, aggregation state, surface chemistry, and functionalization. The high aspect ratio of CNTs may provoke asbestos‐like chronic inflammation and fibrosis, while their hydrophobicity necessitates surface functionalization for adequate dispersion [92]. Metabolic pathways and long‐term biosafety profiles remain insufficiently characterized, and the biosafety of carbon quantum dots and MXene is still under early‐stage investigation. Scalable production and regulatory compliance further constitute substantial barriers. Carbon‐based nanoparticles are therefore more appropriately utilized in localized devices requiring conductivity and mechanical reinforcement, such as conductive nerve conduits, electroactive scaffolds, and biosensing interfaces, rather than as systemic delivery vehicles. Their conductive properties should be calibrated within physiologically relevant ranges to avert cytotoxic consequences arising from excessive conductivity.

#### 3.1.5 Biomimetic Nanoparticles, Cell‐Based Strategies, and Extracellular Vesicles

Representing a vanguard trajectory toward biological emulation in nanocarrier design, biomimetic nanoparticles derive their functionality largely from cloaking synthetic cores with naturally sourced cell membranes, including those from erythrocytes, platelets, macrophages, neutrophils, neural stem cells, and hybrid configurations. Extracellular vesicles (EVs), by contrast, span exosomes of diverse cellular provenance, engineered vesicles bearing exogenous therapeutic cargo, mesenchymal stem cell (MSC)‐derived nanocarriers, and plant‐derived vesicles [93]. A cardinal advantage of these platforms lies in their native biointerfacial attributes: the retained membrane proteome preserves the antigenic repertoire and homing receptor landscape of parental cells, thereby conferring both active targeting and immune evasion [94]. Macrophage membrane‐coated nanoparticles leverage abundant cytokine receptors to achieve inflammatory chemotactic targeting while adsorbing and neutralizing pro‐inflammatory mediators, thus coupling delivery with broad‐spectrum anti‐inflammatory activity [95]. Neuron membrane‐coated mesoporous ceria nanoparticles substantially enhance BBB penetration and reduce immunogenicity by retaining the intrinsic homing behavior of neuronal cells [96]. Neutrophil membrane‐coated nanoparticles embody a tripartite functional paradigm, concurrently engaging inflammatory foci, mitigating calcium overload, and scavenging reactive oxygen species [97]. Within the EV category, MSC‐derived vesicles transport a rich molecular cargo of microRNAs (miRNAs), proteins, and lipids that collectively orchestrate synaptic plasticity, neuronal excitability, myelin homeostasis, neuroprotection, neuroinflammatory modulation, and clearance of pathological protein aggregates [98]. Plant‐derived vesicles, exemplified by ginger‐derived exosome‐like nanoparticles, present a cost‐effective and low‐immunogenicity alternative [99].

Cell‐based regenerative modalities complement this nanoparticle framework. Mesenchymal stromal/stem cells and neural stem cells can support neural restoration through trophic, immunomodulatory, angiogenic, and regenerative actions [100]. Clinical translation, however, remains constrained by poor survival and retention, variable engraftment and biodistribution, manufacturing inconsistency, and potential thrombogenic, immune, or ectopic effects that depend on cell source and administration route [101]. Nanomaterials can partially alleviate these constraints by retaining transplanted cells at lesion sites, shielding them within injectable or implantable scaffolds, and enabling controlled local release of pro‐survival or differentiation cues [102]. In an experimental TBI model, docosahexaenoic acid amplified the effects of neonatal neural stem‐cell transplantation on neurogenesis and motor performance, offering model‐specific evidence that adjunctive biological cues can reinforce cell‐based repair [103].

EVs constitute a cell‐free extension of this regenerative framework while bridging nanomedicine and biomarker development. Within TBI, EV molecular cargo can mirror injury‐associated cellular responses, positioning EVs as candidate sources of circulating biomarker signatures [104]. EVs additionally participate in pathological intercellular signaling and serve as therapeutic targets or delivery vehicles. Such combined pathogenetic, diagnostic, and therapeutic roles have been documented specifically for TBI [105]. The cell‐free format bypasses viable‐cell engraftment yet leaves unresolved uncertainties in source heterogeneity, biodistribution, potency, dosing, and manufacturing consistency.

Clinical translation of EV‐based systems nevertheless confronts formidable manufacturing and standardization hurdles. EV yields remain exceedingly constrained, with conventional MSC‐derived EV production typically falling below 10 mg per liter of culture supernatant [106]. Hypoxic conditioning elevates yield by 1.5‐ to 1.9‐fold, and three‐dimensional culture or genetic engineering may further augment output; however, substantial deficits persist relative to industrial manufacturing requirements [106]. Inter‐batch variability in cellular states engenders pronounced heterogeneity in vesicle size, surface markers, and cargo composition, a challenge compounded by the absence of standardized quality control metrics. Moreover, conventional isolation techniques such as ultracentrifugation can compromise vesicular structural integrity, while immunoaffinity capture suffers from prohibitive costs and limited throughput, imposing severe constraints on large‐scale GMP‐compliant production [107].

Notwithstanding these engineering hurdles, the early clinical feasibility of EV‐based therapy has been explored in neurotrauma. Intrathecal administration of allogeneic umbilical cord MSC‐derived exosomes has completed Phase I evaluation in spinal cord injury patients, with no treatment‐related adverse events observed over a 12‐month follow‐up and Within‐participant improvements documented in American Spinal Injury Association (ASIA) pinprick and light touch scores as well as Spinal Cord Independence Measure III (SCIM III) total scores [108]. Given the early‐phase design and limited evidence base, these observations chiefly substantiate preliminary safety and feasibility rather than therapeutic efficacy. EV‐based systems may confer advantages in neural restoration contexts that demand localized delivery, immunomodulation, or coordinated biological activity; broader clinical adoption will nonetheless depend on advances in scalable manufacturing, product characterization, potency assessment, and batch‐to‐batch consistency.

#### 3.1.6 Hybrid Nanoparticle‐Biomaterial Systems

Hybrid nanoparticle‐biomaterial systems integrate functional nanoparticles into macroscopic three‐dimensional scaffolds, transcending single‐agent delivery toward synergistic modalities that unify structural support, drug delivery, and physical signal transduction. Representative platforms include nanocomposite hydrogels, electrospun nanofiber scaffolds, metal‐organic frameworks (MOFs), conductive nanocomposite hydrogels (CNHs), and 3D‐printed nanobioinks [109]. Four‐dimensional printing, through shape‐memory polymers, stimuli‐responsive hydrogels, and magnetoactive or electroactive composites, introduces programmed temporal changes in scaffold shape, properties, or function, thereby surpassing static three‐dimensional constructs [110]. Such dynamic architectures may prove especially pertinent to neural lesions whose mechanical and biochemical requirements evolve across successive repair phases. This organic‐inorganic hybridization reconciles the biodegradability of organic matrices with the enhanced stability of inorganic constituents, affording expanded design latitude for neural restoration.

Injectable self‐healing nanocomposite hydrogels incorporating metformin nanoparticles concurrently suppress ferroptosis, scavenge reactive oxygen species, and reshape the immune microenvironment in SCI models [111]. Nanomicelle‐assembled multifunctional hydrogels exhibit temperature‐responsive gelation and self‐healing capacity, preserving structural integrity for over one month post‐injection while promoting neuroprotective astrocyte polarization [112]. Laminin‐conjugated aligned nanofiber yarns, serving as electrospun scaffolds, potentiate directional neurite extension through the convergence of topographic and biochemical cues [113]. Hierarchical hydrogel‐nanofiber composites furnish mechanical support calibrated to neural soft tissues while embedding integrated topographical guidance [114]. MOF‐based platforms contribute additional functional dimensions: zinc citrate systems leverage endogenous ROS to trigger on‐demand therapeutic ion release, alleviating oxidative stress and inducing macrophage M2 polarization, while MXene/MOF composite piezoelectric scaffolds generate localized potentials under ultrasound to remodel the immune microenvironment [115, 116]. Conductive cellulose hydrogels incorporating reduced graphene oxide, formulated as 3D‐printable nanobioinks, exhibit superior rheological behavior and printing fidelity, supporting long‐term human astrocyte viability [117].

Nevertheless, escalating system complexity introduces translational risk. Each additional constituent introduces variables that may compromise reproducibility, scalability, sterilization compatibility, storage stability, and regulatory approval [118]. Optimal design therefore favors the simplest architecture capable of fulfilling the essential functions required to surmount key regenerative bottlenecks, while preserving manufacturability and clinical feasibility [119]. Every incorporated component must possess a clearly indispensable role, with its irreplaceability rigorously established through modular dissection controls. The ideal construct is thus the most parsimonious structure that addresses the primary regenerative constraint while remaining compatible with manufacturing, sterilization, storage, and clinical administration. Collectively, the core advantage of these hybrid systems resides in their capacity to synergistically integrate multiple functions through hierarchical design, furnishing an engineering platform that shifts neural restoration from passive filling toward active modulation.

### 3.2 Structure‐Property Relationships: Nanoparticle Physicochemical Characteristics and Biological Outcomes

The in vivo fate of nanoparticles arises from the interplay between their physicochemical attributes and the biological context of administration. Size, morphology, rigidity, surface chemistry, and ligand presentation collectively govern barrier transit, cellular internalization, intracellular trafficking, toxicity, and clearance. Administration route, injury stage, protein‐corona composition, target tissue, and species further modulate these relationships. Physicochemical attributes therefore warrant interpretation as interdependent design variables rather than isolated predictors of biological performance.

#### 3.2.1 Effects of Size, Morphology, and Surface Charge on Neural Cell Interactions

Particle size exerts a context‐dependent influence on blood‐brain barrier (BBB) transport. Size alone does not confer BBB permeability, particularly when the barrier remains structurally intact. A mechanistic investigation demonstrated that two chemically engineered polymer nanomicelles, 20–60 nm in diameter, traversed experimental BBB models via receptor‐mediated or adsorption‐mediated transcytosis. Optimization of receptor avidity or membrane‐penetrating properties enhanced transcytosis by approximately 8‐10‐fold and deep‐brain delivery by 26‐35‐fold relative to corresponding controls [120]. These findings establish the performance of specifically engineered nanomicelles rather than a general capacity of all sub‐100‐nm particles to cross the BBB.

Particles within the 100–200 nm range can attain enhanced lesional exposure when vascular disruption generates sufficient permeability or an enhanced permeability and retention‐like phenomenon, as documented in specific TBI models [121]. The magnitude and duration of this effect track injury type, severity, spatial distribution, and post‐injury phase, and warrant no assumption of uniformity across injured neural tissues. At either size extreme, larger particles exhibit greater susceptibility to mononuclear phagocyte system sequestration, whereas sufficiently small particles may undergo rapid renal clearance [121]. Relevant size boundaries vary with composition, morphology, aggregation state, surface chemistry, and protein‐corona formation rather than constituting fixed thresholds.

Surface charge further governs membrane interactions, protein adsorption, circulation, cellular uptake, and toxicity. In a 42‐member polymer nanoparticle library, surface charge exhibited a stronger association with cellular uptake and cytotoxicity than hydrodynamic diameter within the tested formulation space, and cationic particles displayed greater internalization alongside increased cytotoxicity and nonspecific protein adsorption [122]. Anionic and neutral constructs can show prolonged circulation times but diminished target cell uptake [123]. Within the inflammatory milieu of neural lesions, cationic nanoparticles may preferentially accumulate through electrostatic attraction to damaged membranes, yet a judicious equilibrium between uptake efficiency and cytotoxicity remains imperative.

Particle morphology introduces an additional context‐dependent determinant of cellular interaction. Computational modeling has indicated that nanoparticle geometry can reshape membrane wrapping and clathrin‐mediated internalization, with spherical particles achieving more favorable wrapping than anisotropic counterparts of equivalent volume under modeled conditions [124]. Experimental evidence further reveals shape‐dependent differences in the uptake kinetics and subcellular localization of iron oxide nanorods across distinct cell types [125]. Optimal geometric configuration therefore demands tailoring to the specific target cell population and the prevailing injury microenvironment.

Collectively, size, charge, and geometry dictate the biological fate of nanoparticles. For neural applications, design strategies should prioritize sub‐100 nm diameters to facilitate BBB penetration, near‐neutral surface charges to balance cellular uptake and immunogenicity, and geometric profiles matched to the endocytic characteristics of target cells.

#### 3.2.2 Protein Corona Formation and Its Biological Implications

Upon exposure to biological fluids, nanoparticles rapidly acquire a dynamic biomolecular corona composed of adsorbed proteins, lipids, and other constituents [126]. Formation proceeds via the Vroman effect, a competitive adsorption cascade in which abundant, fast‐associating proteins initially occupy the surface before displacement by less abundant species of higher affinity and longer residence [127]. The corona matures from a loosely associated, rapidly exchanging soft corona into a tightly bound hard corona of extended duration [128], effectively superseding the synthetic identity of the nanoparticle and conferring a new biological identity that governs subsequent physiological interactions.

The protein corona profoundly shapes colloidal stability, pharmacokinetics, biodistribution, targeting fidelity, and cytotoxicity. It redefines surface properties, serves as an immunological recognition signature, and may occlude targeting ligands, thereby attenuating active targeting efficacy [129]. Conversely, deliberate corona engineering through preadsorption of selected proteins enhances organ‐specific delivery, a strategy termed corona‐mediated targeting [59]. The corona may incorporate apolipoproteins such as ApoE that facilitate brain tropism, opsonins like complement component 3 (C3) that promote immune recognition, or dysopsonins such as clusterin that confer stealth‐like behavior [130, 131]. Emerging AI and machine learning frameworks now enable prediction of corona composition and biological outcomes from nanoparticle physicochemical descriptors, informing a data‐driven paradigm for rational design [132].

Cerebrospinal fluid (CSF)‐derived coronas remain substantially underexplored, comprising less than one percent of reported corona investigations [133]. PLGA and PLGA‐PEG nanoparticles exposed to CSF have been shown to acquire coronas with distinct biophysical signatures and compositional profiles relative to serum‐derived counterparts [133]. Critically, CSF‐preconditioned nanoparticles exhibited augmented cellular association with brain‐derived cells compared with uncoated or serum‐preconditioned particles, underscoring the importance of physiologically relevant media in corona studies [133]. These findings carry translational implications for neuronanomedicine design: rather than suppressing corona formation, strategies should harness coronas for ligand‐free targeting, predefine corona composition through rational engineering, and employ machine learning to predict and optimize corona‐dependent outcomes.

#### 3.2.3 Degradation Kinetics and Long‐Term Safety

Long‐term safety remains a critical concern even after successful central nervous system delivery. Degradation kinetics dictate nanoparticle residence time, the chemical identity of breakdown products, and the routes of their elimination. Degradation products need not behave as biologically inert species. In amino acid‐based biomaterials, liberated peptide fragments and amino acids can engage cellular metabolism and signaling, rendering alignment between degradation kinetics and therapeutic timing both a functional design consideration and a safety requirement [134]. Degradable materials such as lipids, PLGA, and oligonucleotides are preferred because they metabolize into endogenous substrates, reducing bioaccumulation risk; their products clear via glymphatic and meningeal lymphatic pathways, although clearance efficiency varies with composition. Inorganic or ion‐releasing materials, by contrast, require stringent control over degradation kinetics, since rapid ionization may trigger intracellular ion overload and toxicity, while non‐degradable residues risk microglial sequestration, lysosomal stress, and impaired autophagic flux [135].

Microglia function as central mediators of nanomaterial clearance, displaying rapid phagocytic activation and more efficient nanoparticle uptake than other glial populations [136]. In a mouse study, both reconstituted high‐density lipoprotein nanoparticles and poly(ethylene glycol)‐b‐poly(lactic acid) nanoparticles displayed brain half‐lives below 5 h. Disruption of the paravascular pathway indicated that approximately 80% of nanoparticle clearance in this model depended on glymphatic transport, with microglia promoting nanoparticle movement toward this route [137]. According to the safety‐degradation window concept, materials should degrade or clear within a safe timeframe after completing their therapeutic function [138]. This requirement necessitates early integration of degradation kinetics into design, achievable through systematic modulation of molecular weight, copolymer composition, crosslinking density, and surface chemistry. Successful clinical translation further demands longitudinal in vivo biodistribution studies, mechanistic evaluation of degradation product effects on immune cells, and standardized neuroimmune toxicity protocols.

### 3.3 Injury‐Specific Material Selection Trade‐Off Matrix

Systematic prioritization of material platforms across injury types can be accomplished through a multidimensional scoring framework that evaluates biological barrier penetration, degradation product safety, functional compatibility, drug‐loading capacity, scalability, and immunogenicity. This framework provides a structured basis for aligning nanoparticle candidates with the distinct pathological imperatives of TBI, SCI, and PNI [139].

The disseminated nature of TBI, marked by widespread parenchymal injury without a demarcated cavity, elevates BBB penetration to paramount importance among selection criteria. Experimental strategies have encompassed ApoE‐modified nanoparticles engaging lipoprotein‐receptor‐associated uptake in brain endothelial models, size‐dependent brain entry for selected gold nanoparticle formulations, and manipulation of the cerebrospinal‐fluid protein corona [140, 141]. Such findings endorse formulation‐specific cerebral delivery while falling short of establishing a universal particle‐size threshold. For diffuse TBI patterns, systemically administered or injectable nanoformulations may prove more compatible with the spatially distributed pathology than focal implants requiring a defined implantation site [142]. Surface charge likewise exerts a substantial influence on systemic brain delivery; near‐neutral, zwitterionic, or mildly anionic surfaces can preserve circulation and favor relative accumulation in injured brain tissue, whereas strongly cationic surfaces may increase off‐target uptake and compromise pharmacokinetic performance [143].

SCI presents a divergent set of requirements, wherein BSCB penetration must be reconciled with the necessity for cystic cavity filling [144]. Injectable hydrogels and nanocomposite scaffolds enable in situ occupation of irregular lesion cavities while affording sustained drug release. Degradation product safety assumes particular criticality in this setting, given the heightened susceptibility of spinal cord parenchyma to inflammatory provocation [145]. Chronic SCI lesions harboring cystic cavities further mandate scaffold materials that simultaneously provide structural support and controlled release functionality.

PNI redirects the priority toward conduit mechanics and topographical guidance. Polymeric or carbon‐based nanoparticles can be processed into anisotropic conduits that emulate the aligned architecture of native nerve fascicles [146]. The comparatively permissive and injury‐disrupted blood‐nerve barrier allows greater material versatility than CNS applications, although long‐segment defects necessitate adequate mechanical robustness and degradation kinetics synchronized with the regenerative timeline [147]. Conductive nanoparticles, including single‐layer graphene and CNTs, have been integrated into nerve guidance conduits (NGCs) to supply electrical cues that potentiate Schwann cell proliferation and myelination [148].

This demand‐driven selection paradigm, progressing from pathological characterization through functional requirements to the nanoparticle properties best suited to fulfill those functions, embodies the material‐level instantiation of the tripartite integrative framework underpinning this review. Building upon this rationale, the ensuing chapter delineates six functional paradigms through which these nanoparticle properties are translated into therapeutic actions, and delineates their respective applicability across TBI, SCI, and PNI.

## 4 Six Functional Paradigms and Their Cross‐Injury Application Spectrum

This framework organizes six functional paradigms along the horizontal axis and three injury categories along the vertical axis, thereby translating nanomaterial properties into defined therapeutic actions. These paradigms progress along a continuum from passive structural support to active intracellular reprogramming. Scaffold construction furnishes provisional matrices, while topographical guidance delivers physical cues for axonal growth. Biomimetic camouflage facilitates barrier traversal and cell‐selective delivery, whereas endogenous responsiveness permits drug activation within pathological niches. External‐field intervention confers wireless spatiotemporal control, and intracellular modulation reprograms cell fate via delivery of ions, nucleic acids, or epigenetic regulators. Collectively, these paradigms drive key phenotypic transitions in critical cell subpopulations, encompassing inflammation regulation, neuroprotection and remyelination, scar inhibition, and vascularization promotion (Figure 3). This graduated progression mirrors escalating complexity and biological sophistication, advancing from material‐dominated passive support toward biologically interactive active intervention.

**FIGURE 3:**
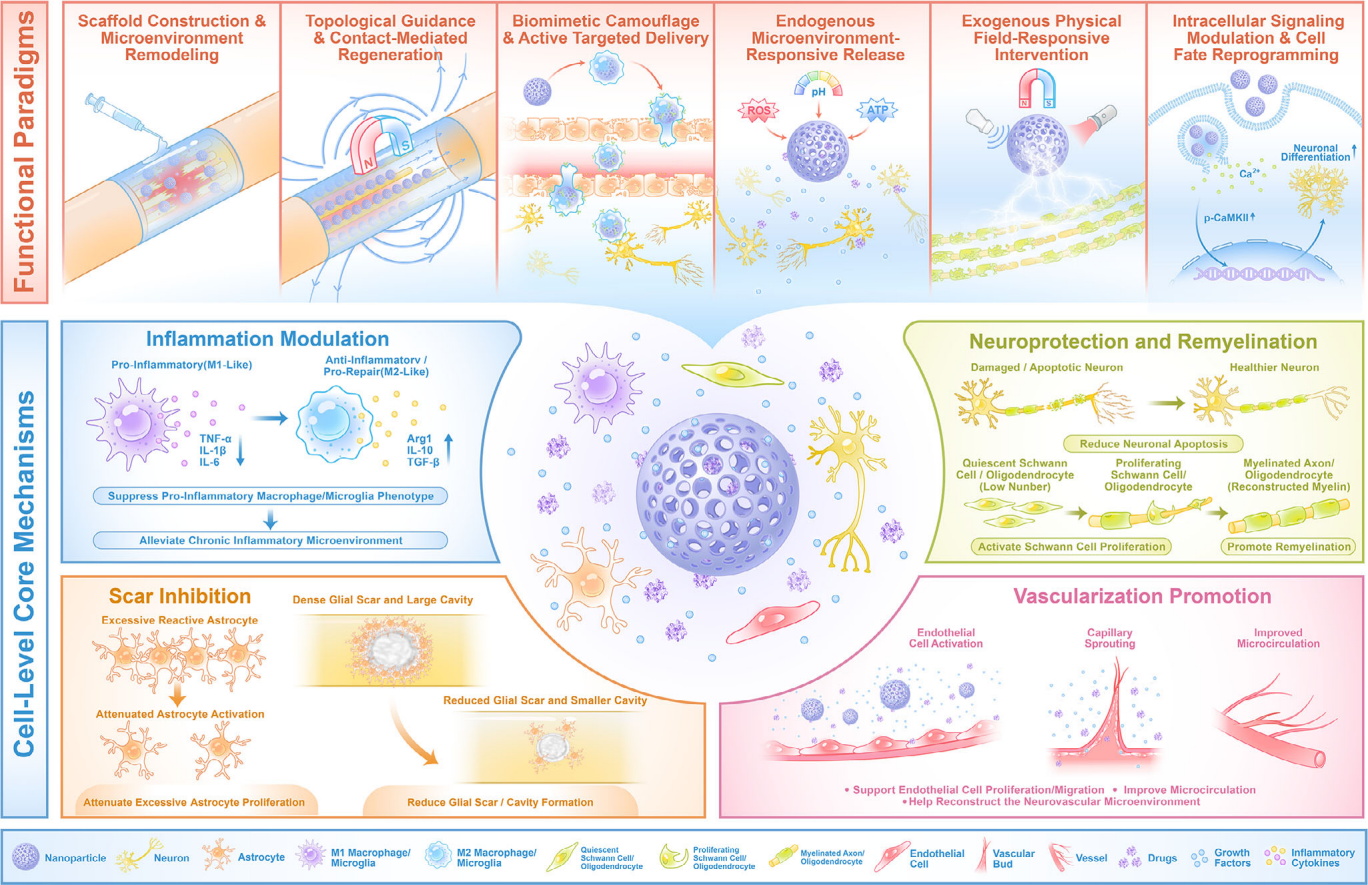
Schematic illustration of six functional paradigms and their regulation of phenotypic changes (inflammation regulation, neuroprotection and remyelination, scar inhibition, vascularization promotion) in key cell subpopulations to promote neural regeneration. The schematic illustration was created using Adobe Illustrator 2025.

### 4.1 Overview of the Six Functional Paradigms: From Material Properties to Therapeutic Functions

Mechanistic complexity and the depth of biological engagement jointly stratify the six paradigms along a continuum from passive structural support to active intracellular reprogramming. Paradigms I and II comprise passive strategies reliant solely on material‐intrinsic properties, addressing tissue defects and axonal guidance without engaging biological recognition systems [149]. Paradigms III through VI constitute active interventions that interface with biological systems at progressively deeper levels. Targeted delivery leverages biomimetic recognition to surmount barriers, whereas responsive release exploits pathological cues as endogenous triggers. External‐field modulation permits activation independently of local signals, while intracellular reprogramming directly reshapes signaling networks and cell fate [150, 151]. Functioning not as mutually exclusive categories, these paradigms form a modular toolkit deployable sequentially or simultaneously according to injury‐specific pathological features and temporal demands. TBI, characterized by diffuse parenchymal injury and BBB disruption, preferentially benefits from paradigms centered on barrier penetration and spatiotemporal modulation [152]. The cystic cavitation and hostile oxidative microenvironment of SCI necessitate scaffold‐mediated filling, topographical guidance, and immunomodulatory intervention [153]. PNI, distinguished by long‐segment defects and a permissive barrier environment, leverages physical guidance, sustained trophic support, and mechanically competent conduits [154]. This demand‐driven selection logic enables the rational integration of multiple paradigms to address the heterogeneous pathophysiology of each injury type, advancing beyond conventional anatomy‐based classification toward pathology‐driven therapeutic design.

Despite encouraging findings, evidence supporting these functional paradigms remains predominantly preclinical, with substantial heterogeneity across species, injury models, treatment protocols, follow‐up durations, and outcome measures. Molecular or histological alterations merit clear distinction from functional improvement verified by behavioral or electrophysiological assessment. The examples below therefore constitute model‐specific proof‐of‐concept evidence rather than demonstrations of comparative superiority or established clinical efficacy.

### 4.2 Paradigm I: Scaffold Construction and Microenvironment Remodeling

Among the six functional paradigms, the most elemental and clinically accessible therapeutic objective lies in defect filling and global microenvironment modulation. Nanocomposite scaffolds, encompassing injectable hydrogels, porous scaffolds, nerve conduits, and electrospun nanofibers, provide volumetric occupancy while enabling temporally programmed release of therapeutic agents. A shared principle across injury types involves supplying a provisional ECM that substitutes for lost tissue and redirects the local milieu away from pathological states. Even when developed within a distinct pathological context, biomaterial‐mediated construction of artificial extracellular matrices through biomineralization, fibrogenesis, or gelation exemplifies a broader design principle: engineered matrices can actively regulate local transport and cellular behavior rather than serve solely as inert fillers [155]. Chemically active scaffolds may additionally confer direct cytoprotection. In an in vitro oxygen‐glucose deprivation model, a thermosensitive poly(ethylene glycol)‐block‐poly(L‐methionine) hydrogel scavenged reactive oxygen species and attenuated mitochondrial oxidative damage and apoptosis [156]. In contrast to paradigms requiring active targeting or external energy input, scaffold construction relies predominantly on material‐intrinsic properties, constituting the most straightforward intervention.

Traumatic injury to the brain leaves a void that lacks intrinsic filling capacity, compelling the use of scaffolds as provisional matrix substitutes. More critically, the biphasic transition from acute inflammation to chronic restoration mandates temporally programmed therapeutic delivery. In a mouse controlled cortical impact model, intraoperative application of a nanohydrogel comprising hemoglobin nanoparticles, brain‐derived neurotrophic factor (BDNF), polydopamine, and dexamethasone achieved sequential release, mitigated acute neuroinflammation and blood‐brain barrier disruption, and improved longer‐term neurovascular and neurological outcomes [157]. In parallel, a GelMA/chitosan methacrylate hydrogel incorporating erythropoietin and interleukin‐4 (IL‐4)‐loaded mesoporous silica nanoparticles reduced neuronal loss and glial scarring, promoted reparative macrophage polarization, and enhanced vascularization, neurogenesis, and neurological performance in a mouse cryogenic brain injury model (Figure 4A) [158].

**FIGURE 4:**
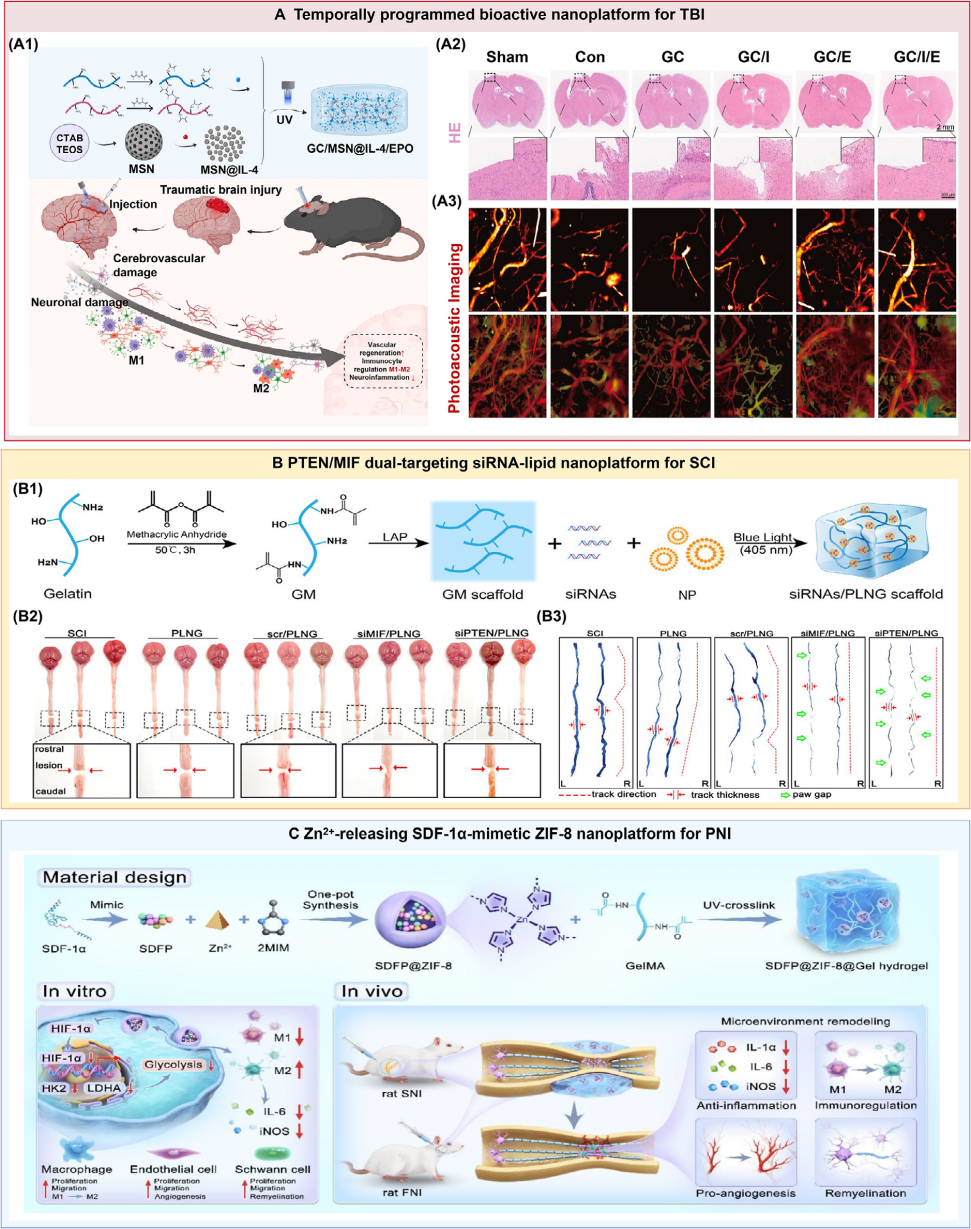
Paradigm I: Scaffold Construction and Microenvironment Remodeling. (A) Temporally programmed bioactive nanoplatform for TBI. Reproduced from [158] under the terms of the CC BY 4.0. Copyright 2024, The Authors. (A1) Schematic illustration of a dual‐drug delivery system pairing IL‐4‐loaded mesoporous silica nanoparticles with free erythropoietin to promote angiogenesis, regulate inflammation, and provide neuroprotection. (A2) H&E staining images showing better restoration of tissue integrity in the TBI model treated with GC/I/E hydrogel. (A3) Photoacoustic imaging demonstrating improved microvascular structure and higher hemoglobin concentration in the GC/I/E hydrogel‐treated group. (B) PTEN/MIF dual‐targeting siRNA‐lipid nanoplatform for SCI. Reproduced with permission [161]. Copyright 2024, American Chemical Society. (B1) Schematic of siRNA/PLNG scaffold synthesis. (B2) Photographs of spinal cords showing more intact spinal cord structure in the siMIF/PLNG and siPTEN/PLNG groups. (B3) Gait analysis revealing better locomotor stability in the siMIF/PLNG and siPTEN/PLNG groups. (C) Zn^2+^‐releasing SDF‐1α‐mimetic ZIF‐8 nanoplatform for PNI. Reproduced with permission [164]. Copyright 2025, Elsevier B.V. Schematic representation of the synthesis of SDFP@ZIF‐8@Gel hydrogel and its application in peripheral nerve repair through neural microenvironment remodeling.

SCI is typified by cystic cavitation, producing irregular voids that host tissue cannot resolve unaided, thereby necessitating injectable hydrogels capable of conformal filling while concurrently reversing the oxidative and inflammatory microenvironment. Photocrosslinkable hydrogels derived from human amniotic membrane have been engineered as bioactive scaffold matrices to support structural and functional restoration following SCI [159]. In rats subjected to thoracic compression injury, lesion‐site delivery of Lightgel/nerve growth factor (NGF)/CeMn nanoparticles attenuated local inflammation and glial activation, enhanced neuronal preservation and regeneration, and improved locomotor performance from day 14, with complementary footprint and histological assessments performed at day 28 [160]. An injectable photo‐crosslinkable lipid nanoparticle‐GelMA scaffold further enables controlled co‐delivery of siRNAs targeting phosphatase and tensin homolog (PTEN) and macrophage migration inhibitory factor (MIF), achieving sustained release over seven days. This system modulates neuronal, microglial, and macrophage activity, promoting axonal regeneration and remyelination; protein array analysis confirms increased growth factor expression and reduced inflammatory markers. These molecular and cellular effects culminate in improved Basso Mouse Scale scores and restoration of ankle joint movement and plantar stepping after only three doses (Figure 4B) [161].

PNI poses a distinct challenge: regenerating axons must bridge physical gaps that exceed the intrinsic regenerative capacity of the distal stump, necessitating nerve guidance conduits that furnish mechanical support and sustained trophic stimulation. Consistent with this multifunctional design principle, a highly biomimetic nerve guidance conduit combining aligned polycaprolactone architectures with a bone marrow‐derived mesenchymal stem cells (BMSCs)‐loaded hyaluronic acid methacrylate (HAMA) hydrogel provided physical guidance, electrical conductivity, and sustained trophic support, thereby enhancing peripheral nerve regeneration in vivo [162]. A bilayer conduit, comprising a high‐concentration GelMA outer layer and a softer lumen embedded with zeolitic imidazolate framework‐8 (ZIF‐8) nanoparticles, provided sustained local NGF delivery and promoted reparative macrophage polarization, axonal regeneration, remyelination, and muscle preservation in a rat sciatic nerve injury model [163]. A stromal cell‐derived factor 1*α* (SDF‐1*α*) peptide‐mimetic ZIF‐8 system further demonstrates that zinc ions liberated during degradation inhibit hypoxia‐inducible factor 1 (HIF‐1) signaling and glycolysis, driving M1‐to‐M2 reprogramming with anti‐inflammatory, pro‐angiogenic, and remyelination effects (Figure 4C) [164]. Controlled NO release from silica nanoparticles incorporated into hydrogels induces early revascularization via CD34+ neovascularization, creating permissive conditions for axonal regeneration and improving functional restoration after acute sciatic nerve crush injury in rats [165]. These findings align with an emerging vessel‐first paradigm in peripheral nerve restoration, wherein early reconstruction of a functional microvascular network is considered a prerequisite for restoring metabolic support, coordinating neurovascular crosstalk, and establishing a permissive environment for subsequent axonal regeneration [166]. Contemporary synthesis of peptide‐incorporated biomaterials for PNI has progressively organized their regenerative effects around axonal elongation, Schwann‐cell support, inflammatory modulation, and vascularization, reinforcing the value of coupling structural conduits with mechanism‐defined biochemical cues [167].

Collectively, although scaffold‐based strategies share the common objectives of defect filling and microenvironment modulation, they must be tailored to each injury type. TBI necessitates temporal programming for its biphasic pathology, SCI demands injectable scaffolds that combine conformal filling with immunomodulatory and antioxidant capacity, and PNI requires mechanically competent conduits with sustained trophic and angiogenic support. The fundamental dependence on material‐intrinsic properties renders this paradigm the most accessible and clinically viable entry point for nanomaterial‐based neural restoration.

### 4.3 Paradigm II: Topographical Guidance and Contact‐Mediated Regeneration

Oriented physical cues that guide axonal regeneration can be imparted through two principal strategies: magnetic nanoparticles that assemble into anisotropic architectures within hydrogels or conduits under external fields, and aligned electrospun nanofibers. This paradigm addresses the dual imperatives of directing axonal outgrowth and restoring physical continuity across lesion gaps, functioning as the second passive guidance modality. Unlike scaffold construction, which centers on defect filling and biochemical modulation, this approach operates exclusively through physical mechanisms, requiring neither biochemical signaling nor active targeting.

In SCI, cavitation, cystic degeneration, and glial scarring create physical discontinuities that obstruct axonal traversal, while the highly ordered white matter tracts require topographical cues for meaningful regeneration. Effective deployment of this paradigm therefore necessitates concurrent microenvironmental remodeling to overcome the inhibitory milieu. Under static magnetic fields, silica‐coated magnetic nanoparticles self‐assemble into nanoscale chains that form three‐dimensional anisotropic architectures capable of directing neurite extension. Upon transplantation into lesion cavities, these aligned structures promoted neural regeneration and improved motor outcomes in a rat SCI model (Figure 5A) [168]. Iron‐chelated silk fibroin microfibers (Fe^3+^‐mSF) offer a nanoparticle‐free, magnetically responsive alternative; following functionalization, these microfibers demonstrate robust stability under physiological conditions with minimal iron release over extended periods [169]. When incorporated into injectable in situ‐crosslinkable hydrogels, Fe^3+^‐mSF maintains injectability and gelation kinetics while exhibiting cytocompatibility with neuronal cells. Magnetic resonance imaging (MRI) analyses have confirmed magnetically induced alignment of Fe^3+^‐mSF‐loaded hydrogels without fiber pull‐out, establishing this system as a promising injectable scaffold capable of in situ topographical alignment for spinal cord restoration. Safety considerations differentiate the two approaches: silica‐coated nanoparticles display acceptable biocompatibility, whereas the iron‐chelated microfiber platform inherently circumvents the long‐term retention concerns associated with particulate magnetic materials.

**FIGURE 5:**
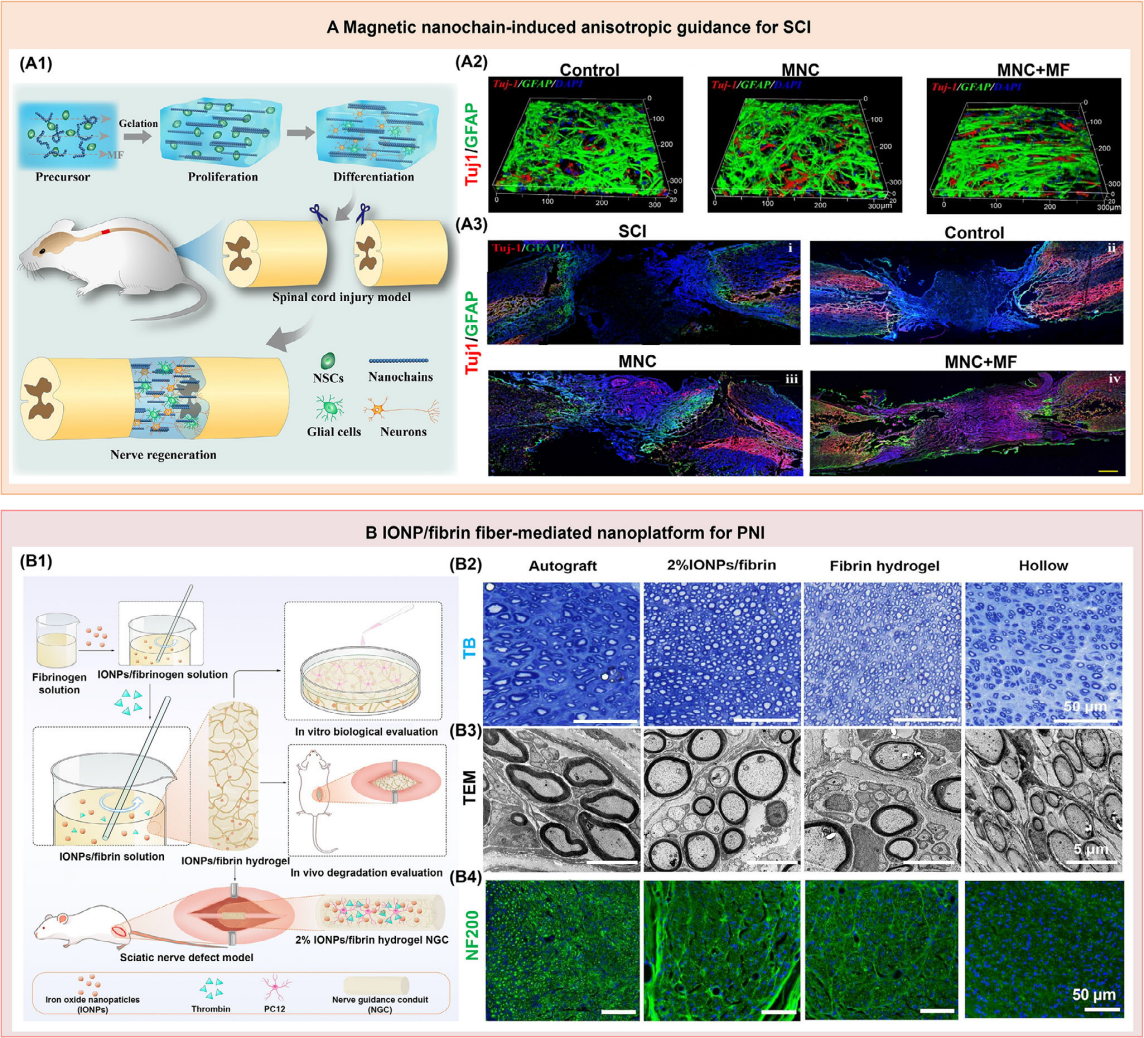
Paradigm II: Topographical Guidance and Contact‐Mediated Regeneration. (A) Magnetic nanochain‐induced anisotropic guidance for SCI. Reproduced with permission [168]. Copyright 2024, Elsevier B.V. (A1) Schematic diagram of magnetic hydrogel constructs for improving nerve reconnection in SCI rats. Anisotropic nerve assembly was achieved by magnetic nanochains and magnetic actuation, which could promote the regeneration and reconnection of nerve cells in SCI rats. (A2) IF staining (red: Tuj1; green: GFAP) showing more oriented alignment of NSCs on magnetically induced hydrogels. (A3) IF staining (red: Tuj1; green: GFAP) showing a higher neuronal area and a smaller lesional glial area in the magnetically induced hydrogel‐treated group, respectively. (B) IONP/fibrin fiber‐mediated nanoplatform for PNI. Reproduced from [173] under the terms of the CC BY 4.0. Copyright 2024, The Author(s). (B1) Schematic illustration of the IONPs/fibrin hydrogel fabrication procedure, in vitro biocompatibility evaluation, and in vivo degradation evaluation. The composite hydrogel was preloaded into a chitosan tube to bridge a 10‐mm gap in the rat sciatic nerve. (B2‐B3) TB staining and TEM showing better myelination in the IONPs/fibrin‐treated group, respectively. (B4) NF200 (green) IF showing more regenerated axons in the IONPs/fibrin‐treated group.

The PNS constitutes an intrinsically permissive environment for contact‐mediated guidance. In the absence of appropriate physical cues, regenerating axons traversing a nerve gap tend to form disorganized neuromas rather than functional connections [170]. Anisotropic conduits that recapitulate the aligned architecture of Büngner bands provide essential pathways from proximal to distal stumps [171, 172]. Magnetic iron oxide nanoparticles incorporated into cellulose nanofiber hydrogels generate ordered anisotropic topographies under external static fields, directing oriented Schwann cell growth. In a rat 10‐mm sciatic nerve gap model, these iron oxide nanoparticles (IONPs)/cellulose hydrogels improved motor recovery, with selected late‐stage outcomes approaching those observed in the autograft group at 12 weeks post‐operation (Figure 5B) [173]. Beyond passive topographical guidance, a Fe3O4‐integrated anisotropic chitosan scaffold combined with static magnetic stimulation likewise enhanced Schwann cell proliferation, migration, and orientation in vitro and promoted regeneration across a peripheral nerve defect in vivo [174]. These findings underscore that topographical cues are most efficacious when integrated with responsive material features that provide both physical direction and biological activation. The long‐term biocompatibility of iron oxide nanoparticles in peripheral nerve applications has been generally affirmed, although particle retention and degradation kinetics warrant careful consideration for clinical translation [175].

By contrast, TBI offers a restricted scope for topographical intervention. The diffuse, disorganized nature of parenchymal damage lacks the coherent axonal tracts that define SCI and PNI, rendering unidirectional physical cues largely inapplicable. For brain injuries, microenvironmental modulation and targeted delivery strategies assume clear precedence over physical guidance approaches. The utility of this paradigm is thus contingent upon the presence of ordered tissue architecture: indispensable for SCI and PNI, where structural continuity and directed axonal growth are paramount, yet of marginal relevance for the fundamentally disorganized pathology of TBI.

### 4.4 Paradigm III: Biomimetic Camouflage and Active Targeted Delivery

Engineered cell membrane coatings and ligand‐functionalized nanoparticles, incorporating antibodies, peptides, aptamers, and carbohydrates, enable active barrier traversal, lesional enrichment, and cell‐type‐selective delivery while prolonging circulatory residence. This paradigm transcends the passive reliance on transient and spatially heterogeneous barrier leakage, permitting BBB or BSCB crossing and precise anchoring to target cells. It thus constitutes the first active intervention modality, with greatest demand in TBI and SCI where barrier integrity poses a major obstacle, whereas PNI, despite its less restrictive barrier, still benefits from selective delivery to defined neural subsets.

Within TBI, the compromised BBB, together with oxidative stress and neuroinflammation, calls for platforms that simultaneously penetrate the barrier and accumulate at lesions. In a mouse TBI model, neutrophil‐like membrane‐functionalized nanoparticles exploit inflammatory chemotactic receptors, including C‐X‐C chemokine receptor type 4 (CXCR4) and integrin β1, to anchor to activated endothelial cells, while co‐delivering nimodipine for calcium influx inhibition and Mn_3_O_4_ for ROS scavenging, thereby reducing neuronal apoptosis and improving memory‐related performance [97]. M2 macrophage membrane‐coated nanoparticles leverage immune evasion and inflammatory tropism to deliver microRNA‐21 to injured neurons, attenuating apoptosis and promoting neurological outcomes after TBI [176]. Ligand‐based approaches offer precision without the complexity of membrane engineering: CAQK peptide‐modified nanoparticles recognize exposed ECM components, enabling lesion‐localized accumulation that interrupts the ROS‐neuroinflammation cycle while preserving barrier integrity [177]; whereas neutrophil‐targeting hesperetin nanoparticles rapidly accumulated and exhibited prolonged retention in injured brain tissue in mice, where sustained hesperetin release reduced pro‐inflammatory cytokine production and microglial and astrocytic activation, promoted a shift from M1‐like to M2‐like microglial phenotypes, enhanced regulatory T‐cell infiltration, and alleviated neuroinflammation and neurological deficits [178]. Beyond single‐stage targeting, hierarchical architectures achieve spatiotemporally ordered delivery through sequential barrier penetration and cell‐type‐specific recognition; one such system uses endothelial membrane‐camouflaged liposomes to cross the BBB, then hitchhikes on neutrophils via elastase binding, with matrix metalloproteinase (MMP) cleavage exposing a concealed Tet1 peptide for secondary neuron‐specific targeting and pyroptosis inhibition, reducing tissue injury and improving behavioral outcomes in mice with TBI [179]. Live‐cell hitchhiking platforms similarly exploit platelet migration: fucoidan‐modified liposomes bind P‐selectin on activated platelets, which transport the triiodothyronine across inflamed cerebrovascular endothelium to sites of injury (Figure 6A) [180]. Collectively, these platforms progress from single‐function systems to multilayered architectures that achieve selective enrichment and active barrier penetration, ultimately improving histological and functional outcomes.

**FIGURE 6:**
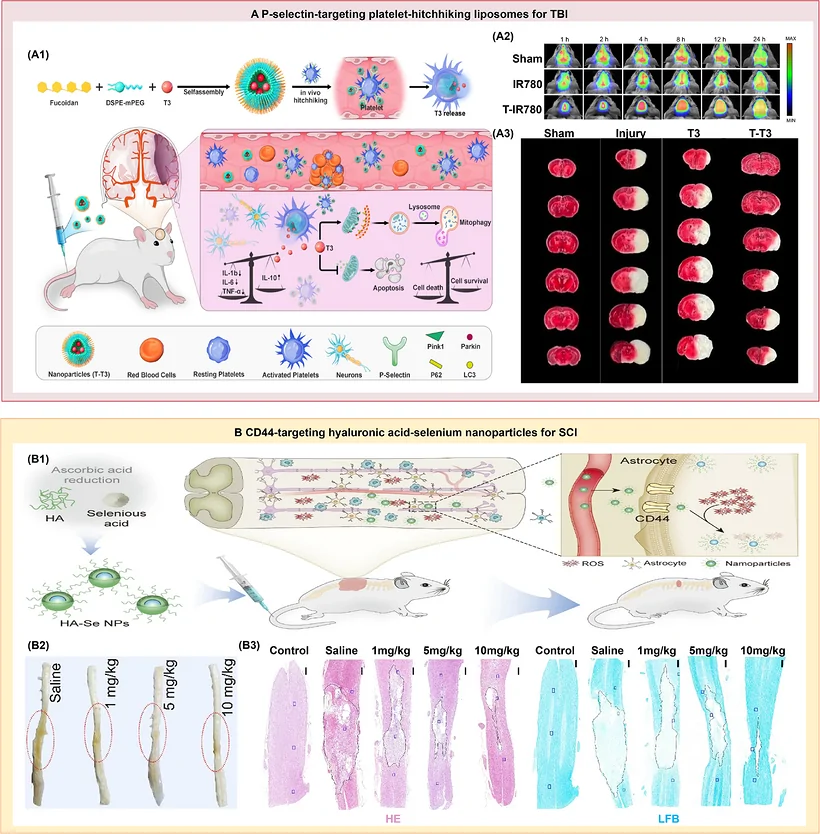
Paradigm III: Biomimetic Camouflage and Active Targeted Delivery. (A) P‐selectin‐targeting platelet‐hitchhiking liposomes for TBI. Reproduced with permission [180]. Copyright 2024, The Elsevier Inc. (A1) Schematic illustration showing the synthesis of triiodothyronine (T3) nanoparticles (T‐T3) and their hitchhiking on activated platelets in vivo. Upon reaching the injury site, T3 is released and provides neuroprotection by reducing neuroinflammation, inhibiting apoptosis, and inducing autophagy. (A2) Representative NIR‐II images demonstrating the favorable targeting capability of T‐T3 for precise delivery to the cerebral injury region. (A3) TTC staining showing a smaller infarct area in the T‐T3‐treated group. (B) CD44‐targeting hyaluronic acid‐selenium nanoparticles for SCI. Reproduced from [184] under the terms of the CC BY 4.0. Copyright 2024, The Authors. (B1) Schematic illustration of the preparation of CD44‐targeting HA‐Se NPs for promoting functional recovery after SCI. (B2) Photographs of spinal cords showing improved spinal cord integrity in the HA‐Se NPs‐treated group. (B3) H&E staining and LFB staining of spinal cord sections showing better axonal and myelin sheath structural recovery in the HA‐Se NPs‐treated group.

SCI presents the dual challenge of traversing the disrupted BSCB while navigating a hostile oxidative and inflammatory microenvironment. In a mouse spinal cord contusion model, macrophage membrane‐coated nanoparticles with inflammatory homing capability deliver curcumin to the lesion site, attenuating ferroptosis and central inflammation while significantly improving hindlimb locomotor performance [95]. Microglia membrane‐coated nanoparticles exploit the intrinsic tropism of microglia toward inflammatory foci to deliver zinc sulfide nanoparticles that release H_2_S and Zn^2+^, inhibiting microglial activation through nuclear factor kappa B (NF‐κB) modulation and promoting axonal growth and locomotor recovery [181]. A neutrophil membrane‐coated mesoporous Prussian blue platform delivered hederagenin to contused spinal cord tissue, coupling lesion recruitment with antioxidant and anti‐inflammatory activity in mice [182]. For cell‐type‐selective delivery without membrane coating, mannose‐modified lipid nanoparticles target mannose receptor CD206‐enriched inflammatory macrophages and microglia, sustaining local translation of erythropoietin, thereby reducing inflammation and neuronal loss, preserving serotonergic axons, and improving motor outcomes in mice with SCI [183]. CD44‐targeting hyaluronic acid‐selenium nanoparticles exploit CD44 overexpression on reactive astrocytes to promote selective uptake, scavenge free radicals, and suppress pro‐inflammatory cytokine secretion, thereby supporting axonal preservation and improving locomotor performance in rats with SCI (Figure 6B) [184]. These complementary targeting mechanisms collectively address the complexity of the SCI microenvironment, converging on neural tissue preservation and locomotor restoration.

PNI, characterized by relatively accessible nerve anatomy, offers a more permissive context for therapeutic delivery. Although the peripheral nerve barrier is less restrictive than that of the CNS, active targeting strategies remain relevant for cell‐selective delivery, particularly when precise spatial modulation of Schwann cells or macrophages is required. Targeting principles established for CNS applications, including membrane coating and ligand modification, are broadly transferable to peripheral nerve settings, where reduced barrier constraints may permit simpler ligand‐directed formulations to achieve sufficient precision without the complexity of multilayered membrane systems.

### 4.5 Paradigm IV: Endogenous Microenvironment‐Responsive Release

Nanosystems engineered to respond to lesion‐specific pathological cues, including reactive oxygen species, acidic pH, matrix metalloproteinase activity, iron overload, and elevated ATP, achieve spatially and temporally confined drug release exclusively within the injured microenvironment while remaining inert in healthy tissue. The secondary injury cascade following neural trauma unfolds as a dynamic, interconnected network of signals with distinct spatial and temporal architectures. These endogenous cues not only propagate tissue damage but also provide exploitable triggers for therapeutic activation. Conventional delivery platforms cannot discriminate between pathological and healthy tissue, resulting in heterogeneous drug distribution and suboptimal efficacy. Endogenous‐responsive strategies surmount this limitation through nanocarriers that recognize lesion‐specific biochemical signatures, preserving systemic inertness and activating solely upon encountering the pathological milieu. This paradigm thereby achieves localized therapeutic action while minimizing systemic exposure and off‐target effects, constituting the second active intervention modality.

The pathological landscape of TBI encompasses oxidative stress, tissue acidosis, and nucleic acid release from damaged cells, creating a multifaceted trigger environment that necessitates multi‐responsive delivery platforms. An ROS‐responsive nanoplatform incorporating ultrasmall ceria nanoparticles and interleukin‐1 receptor‐associated kinase 4 (IRAK‐4) siRNA within a degradable matrix achieves on‐demand release upon lesional accumulation (Figure 7A) [185]. Exposure to elevated ROS disintegrates the matrix, liberating ceria nanodots for ROS scavenging and oxygen generation while IRAK‐4 siRNA suppresses pro‐inflammatory signaling and reprograms microglia toward an anti‐inflammatory phenotype. This dual action concurrently attenuates oxidative stress, hypoxia, and neuroinflammation, preserving neuronal integrity and improving neurological outcomes. Neutrophil membrane coating further enhanced recruitment to injured tissue, and treatment in mice reduced oxidative stress, hypoxia, neuroinflammation, and neurological deficits after TBI. Beyond ROS, cell‐free DNA released from injured tissue acts as an additional endogenous trigger for neuroinflammation through Toll‐like receptor 9 (TLR9) activation. A pH‐sensitive cationic nanoparticle system has been devised to address this pathway [186]. These particles sequester free DNA at extracellular inflammatory foci and within acidic endosomal compartments, suppressing TLR9‐mediated cytokine production. In a murine TBI model, this dual‐mode strategy effectively reduces inflammatory cytokines without the toxicity associated with conventional cationic carriers.

**FIGURE 7:**
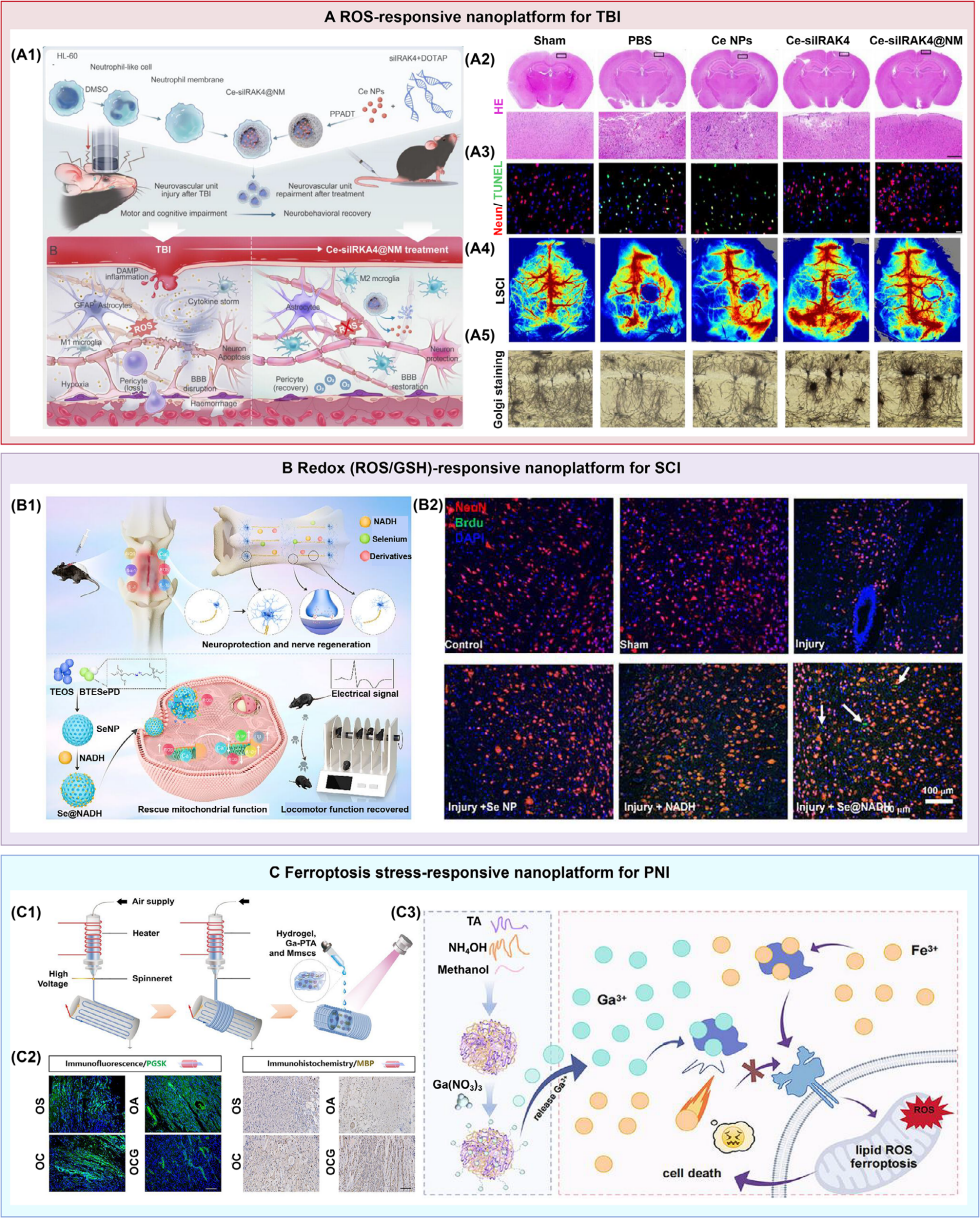
Paradigm IV: Endogenous Microenvironment‐Responsive Release. (A) ROS‐responsive nanoplatform for TBI. Reproduced with permission [185]. Copyright 2025, Wiley‐VCH GmbH. (A1) Schematic illustration of the development of a multimodal nanodrug Ce‐siIRAK4@NM NPs targeting multiple key pathological events in NVU disruption to mitigate secondary TBI damage. (A2) Representative H&E staining of brain sections showing a smaller lesion area in the Ce‐siIRAK4@NM NPs‐treated group. (A3) Representative IF staining of brain sections (NeuN in red / TUNEL in green) showing reduced apoptosis in the Ce‐siIRAK4@NM NPs‐treated group. (A4) LSCI of cerebral blood flow showing better perfusion improvement in the Ce‐siIRAK4@NM NPs‐treated group. (A5) Golgi staining of neuronal dendrites showing better recovery of dendritic spine density and dendritic structure in the Ce‐siIRAK4@NM NPs‐treated group. (B) Redox (ROS/GSH)‐responsive nanoplatform for SCI. Reproduced from [187] under the terms of the CC BY 4.0. Copyright 2025, The Authors. (B1) Schematic illustration of the synthesis of Se@NADH nanotherapeutics and their neuroprotective mechanisms against SCI. (B2) IF staining (NeuN in red / BrdU in green) showing a greater number of regenerated neurons in the Se@NADH‐treated group. (C) Ferroptosis stress‐responsive nanoplatform for PNI. Reproducedfrom [191] under the terms of the CC BY 4.0. Copyright 2025, The Authors. (C1) Schematic illustration of the fabrication of electrospun nerve conduits incorporating hydrogel, Ga‐PTA, and MMSCs. (C2) PGSK IF for ferrous ion and MBP IHC images showing reduced ferroptosis levels and better myelin recovery in the OCG‐treated group. (C3) Schematic illustration showing that Ga‐PTA nanoparticles competitively inhibit excessive ferroptosis and establish a regeneration‐promoting niche. (OS, aging sham group; OA, aging autologous nerve anastomosis group; OC, aging PNI group treated with MMSC‐loaded nerve conduits; OCG, aging PNI group treated with Ga‐PTA and MMSC‐loaded nerve conduits.).

SCI presents an equally hostile microenvironment, where oxidative stress, acidosis, and iron dysregulation converge, demanding responsive systems that address these interconnected signals while traversing the disrupted BSCB. To target mitochondrial dysfunction, a redox‐responsive strategy employing diselenide‐containing mesoporous silica nanoparticles (Se@NADH) releases reduced nicotinamide adenine dinucleotide (NADH) upon ROS or GSH exposure through cleavage of diselenide bonds (Figure 7B) [187]. The liberated NADH regenerates oxidized nicotinamide adenine dinucleotide (NAD^+^) by reacting with deleterious ROS, counteracting mitochondrial dysfunction, elevating ATP synthesis, promoting axonal regeneration, and inhibiting glial scar formation. Complementing this ROS‐responsive system, a metal‐phenolic nanoparticle system incorporated into aligned decellularized ECM fibers released magnesium ions and epigallocatechin gallate under acidic conditions, attenuating excitotoxicity and neuroinflammation while promoting directional axonal growth and motor recovery in a rodent SCI model [188]. To further target iron dysregulation, an ROS‐responsive nanoparticle platform delivers a ferroptosis inhibitor (Feborastatin‐1) in conjunction with human umbilical cord MSCs, significantly suppressing ferroptosis and inflammatory responses, while improving BBB scores, CatWalk parameters, and motor‐evoked potentials over six weeks [189]. These responsive platforms collectively illustrate that distinct pathological signals, namely ROS, pH, and iron dysregulation, can be harnessed as intrinsic triggers for precision therapy within the SCI microenvironment.

PNI, particularly in age‐related nerve damage, involves a ferroptosis stress‐enriched microenvironment that suppresses Schwann cell function and exacerbates senescence‐associated secretory phenotype expression, constituting a distinctive pathological signature for responsive intervention [190]. In aged rats with sciatic nerve injury, melt‐electrowritten composite nerve conduits incorporating Ga‐PTA nanoparticles respond to ferroptotic stress by releasing Ga^3+^, which competitively inhibits excessive ferroptosis and rescues the impaired dedifferentiation of senescent Schwann cells, restoring their regenerative capacity and improving functional outcomes (Figure 7C) [191]. A bioresponsive NGC based on MXene‐MnO_2_ nanocomposite hydrogels utilizes MnO_2_ nanoparticles as catalytic antioxidant modules to continuously neutralize ROS, alleviate chronic inflammation, and promote timely reparative macrophage polarization in a PNI model [89]. Gold nanoparticles embedded within chitosan/poly(vinyl alcohol) (PVA) hydrogel networks achieve dual functions of ROS scavenging and paracrine stimulation, protecting MSCs by reducing intracellular ROS levels while enhancing cell survival and promoting growth factor secretion [192].

Endogenous‐responsive delivery systems share a unifying design philosophy across all three injury types: exploiting the unique pathological signatures of each injury to trigger therapeutic release with high spatial and temporal precision. Although the specific pathological signals differ, the underlying principle remains consistent. The injury microenvironment itself serves as an intrinsic control mechanism for precision therapy, transforming the pathological milieu from a barrier to treatment into an ally for targeted intervention.

### 4.6 Paradigm V: Exogenous Physical Field‐Responsive Intervention

Exogenous physical stimuli, including magnetic fields, ultrasound, and light, afford spatiotemporally precise therapeutic intervention with exacting control over temporal onset, intensity, and spatial confinement [193]. These capabilities synchronize therapeutic activation with optimal treatment windows and enable repeated intervention independent of unpredictable endogenous signaling. The deep tissue penetrance of magnetic fields and ultrasound further permits activation even where the BBB remains intact or endogenous signal gradients prove inadequate. This paradigm thus addresses clinical scenarios that demand external energy input for precise spatiotemporal regulation, constituting the third active intervention modality.

Magnetic platforms represent the most extensively investigated external stimulus strategy for TBI. When integrated into bioactive hydrogels, magnetic nanoparticles respond to external fields by imposing mechanical strain on the gel network, generating dynamic fluctuations in matrix stiffness. In a rat TBI model, a magnetic on‐off hydrogel transduced pulsed magnetic input into matrix vibration and transient receptor potential vanilloid 4 (TRPV4)‐dependent calcium influx, thereby promoting BMSC elongation and neural differentiation while improving neurological and tissue‐repair outcomes [194]. Magneto‐electric nanomaterials furnish a complementary strategy by coupling magnetic responsiveness with electrical stimulation. Heterojunction‐enhanced magnetoelectric nanoparticles generate continuous electron flow under magnetic fields; when embedded within hydrogel matrices that recapitulate neural tissue properties, these platforms enable wireless magnetoelectric stimulation of neural stem cells, promoting neuronal differentiation, accompanied by improved cognition‐ and memory‐related performance in a preclinical TBI model (Figure 8A) [195].

**FIGURE 8:**
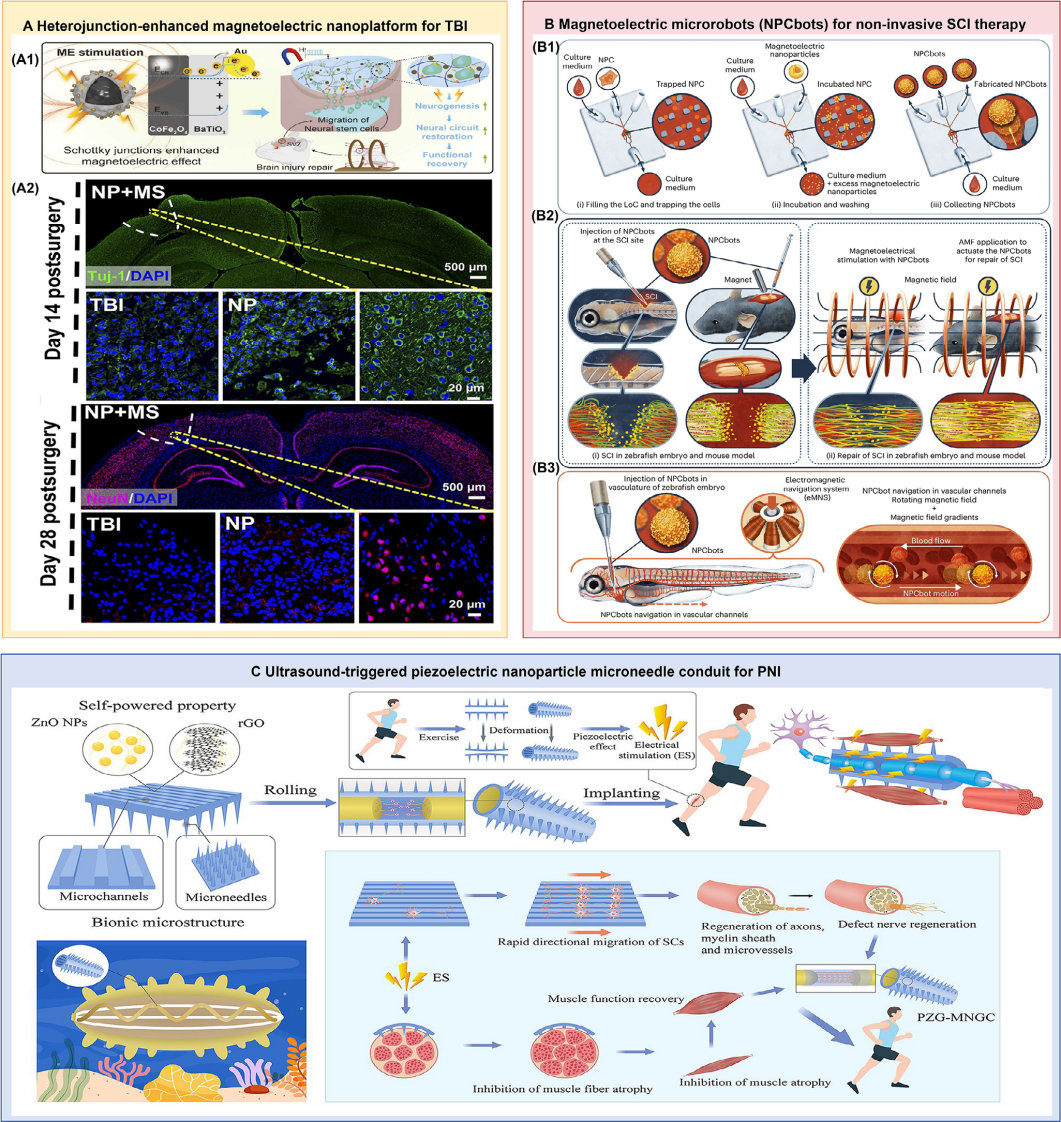
Paradigm V: Exogenous physical field‐responsive intervention. (A) Heterojunction‐enhanced magnetoelectric nanoplatform for TBI. Reproduced with permission [195]. Copyright 2025, Elsevier Ltd. (A1) Schematic illustration of the magnetoelectric nanoplatform and its mechanism for neural stem cell migration, neurogenesis, and brain injury repair under ME stimulation. (A2) IF staining (Tuj1 in green and NeuN in red) showing enhanced neurogenesis in the magnetoelectric nanoplatform‐treated group. (B) Magnetoelectric microrobots (NPCbots) for non‐invasive SCI therapy. Reproduced from [196] under the terms of the CC BY 4.0. Copyright 2025, The Author(s). (B1) Schematic illustration of NPCbot fabrication, including NPC culture, cell trapping, incubation with magnetoelectric nanoparticles, and collection. (B2) Schematic illustration of NPCbot injection at the SCI site and AMF‐activated repair in zebrafish embryo and mouse models. (B3) Schematic illustration of NPCbot navigation in vascular channels under an electromagnetic navigation system. (C) Ultrasound‐triggered piezoelectric nanoparticle microneedle conduit for PNI. Reproduced with permission [202]. Copyright 2024, American Chemical Society. Schematic illustration showing a sea cucumber‐inspired microneedle nerve guidance conduit loaded with piezoelectric zinc oxide nanoparticles to synergistically inhibit muscle atrophy and promote nerve regeneration.

Magnetic approaches prove equally well suited to SCI, capitalizing on deep tissue penetrance for non‐invasive therapeutic activation. Magnetic navigation directed biohybrid microrobots integrating human induced pluripotent stem cell (iPSC)‐derived neural precursors with magnetoelectric nanoparticles were delivered to injured tissue in zebrafish and mouse SCI models. Alternating‐field stimulation promoted neuronal differentiation and graft integration, and mice with complete spinal cord transection displayed improved motor performance within four weeks (Figure 8B) [196]. Ultrasound‐driven piezoelectric nanoparticles offer spatially focused stimulation as a complementary modality: lithium‐modified zinc oxide nanoparticles internalized by neural stem cells and activated by ultrasound enhanced extracellular signal‐regulated kinase (ERK)‐dependent neuronal differentiation and, when combined with a collagen–genipin hydrogel, promoted neural repair after SCI [197]. Optogenetic strategies employing near‐infrared‐triggered upconversion nanoparticles further extend the repertoire. Glutamate‐conjugated upconversion nanoparticles, selectively internalized by postsynaptic interneurons innervated by corticospinal tract axons, function as internal light converters and nanobeacons to guide axonal extension [198]. This in situ optogenetic modulation successfully supported the formation of functional connections between corticospinal axons and spinal interneurons, as confirmed by live‐cell recording, in vivo calcium imaging, and behavioral assessments. Integrated platforms combining these nanomaterials with hydrogel scaffolds achieve both structural continuity and active electrophysiological modulation, addressing the dual challenges of physical disconnection and electrophysiological silence in the injured spinal cord.

PNI benefits from analogous external stimulus paradigms adapted to peripheral nerve anatomy. Ultrasound‐responsive piezoelectric nanofiber conduits doped with barium titanate generate wireless electrical stimulation under ultrasound to induce oriented neurite extension, while thermoresponsive hydrogel contraction enables controlled NGF release. In a rat sciatic nerve defect model, repeated ultrasound treatment over 12 weeks enhanced axonal regeneration, remyelination, muscle preservation, electrophysiological recovery, and behavioral performance relative to non‐piezoelectric and non‐stimulated controls [199]. A self‐powered poly(vinylidene fluoride‐co‐trifluoroethylene) [P(VDF‐TrFE)]/reduced graphene oxide/PCL scaffold modulated neutrophil extracellular trap formation and inflammatory cytokine profiles while promoting revascularization in a peripheral nerve injury model [200]. In a further platform, umbilical cord MSCs encapsulated within a molybdenum nanoparticle‐doped GelMA hydrogel were coupled to a wireless electrical stimulator, and electrical input promoted Schwann‐like differentiation and neurotrophin secretion, thereby enhancing peripheral nerve regeneration in vivo [201]. Biomimetic microneedle conduits incorporating piezoelectric nanoparticles simultaneously stimulate denervated muscles while guiding Schwann cell migration, ultimately inhibiting muscle atrophy and restoring nerve function eight weeks after implantation in a rat nerve defect model (Figure 8C) [202].

Externally responsive systems possess a decisive advantage over endogenous strategies: precise temporal control independent of pathological signal evolution. TBI profits from magnetic and magnetoelectric platforms that combine mechanical and electrical stimulation; SCI leverages magnetic navigation, ultrasound‐driven effects, and optogenetic approaches; PNI employs ultrasound and electrical stimulation tailored to peripheral regeneration. The unifying principle resides in harnessing external energy to actively drive therapeutic function, thereby achieving spatiotemporal precision unattainable by passive or endogenously triggered systems.

### 4.7 Paradigm VI: Intracellular Signaling Modulation and Cell Fate Reprogramming

Endocytosed nanosystems directly engage intracellular signaling networks, releasing ions, delivering nucleic acids, or introducing epigenetic modulators to reprogram cell fate. Unlike paradigms that provide exogenous support, this approach fundamentally reconfigures cellular behavior, spanning proliferation, migration, differentiation, and phenotypic polarization, thereby constituting the deepest level of active intervention among the six functional paradigms.

The constrained regenerative competence of the adult brain, compounded by persistent neuroinflammation after TBI, necessitates direct modulation of intracellular pathways governing neural stem cell fate and microglial function. Hierarchical miRNA‐CaCO3@PLGA nanoparticles, after endocytosis by neural stem cells, released Ca^2+^ and microRNA‐124 and thereby coordinated calcium‐ and miRNA‐dependent stimulation of neuronal differentiation. Transplantation of nanoparticle‐treated cells into TBI mice augmented neuronal differentiation and improved memory‐ and cognition‐related performance (Figure 9A) [203]. In a complementary metabolic approach examined in rat hypoxic‐ischemic brain injury, nicotinamide‐loaded peptoid nanotubes localized to microglia, supported ATP regeneration, diminished inflammatory cytokine production, and restricted tissue injury [204].

**FIGURE 9:**
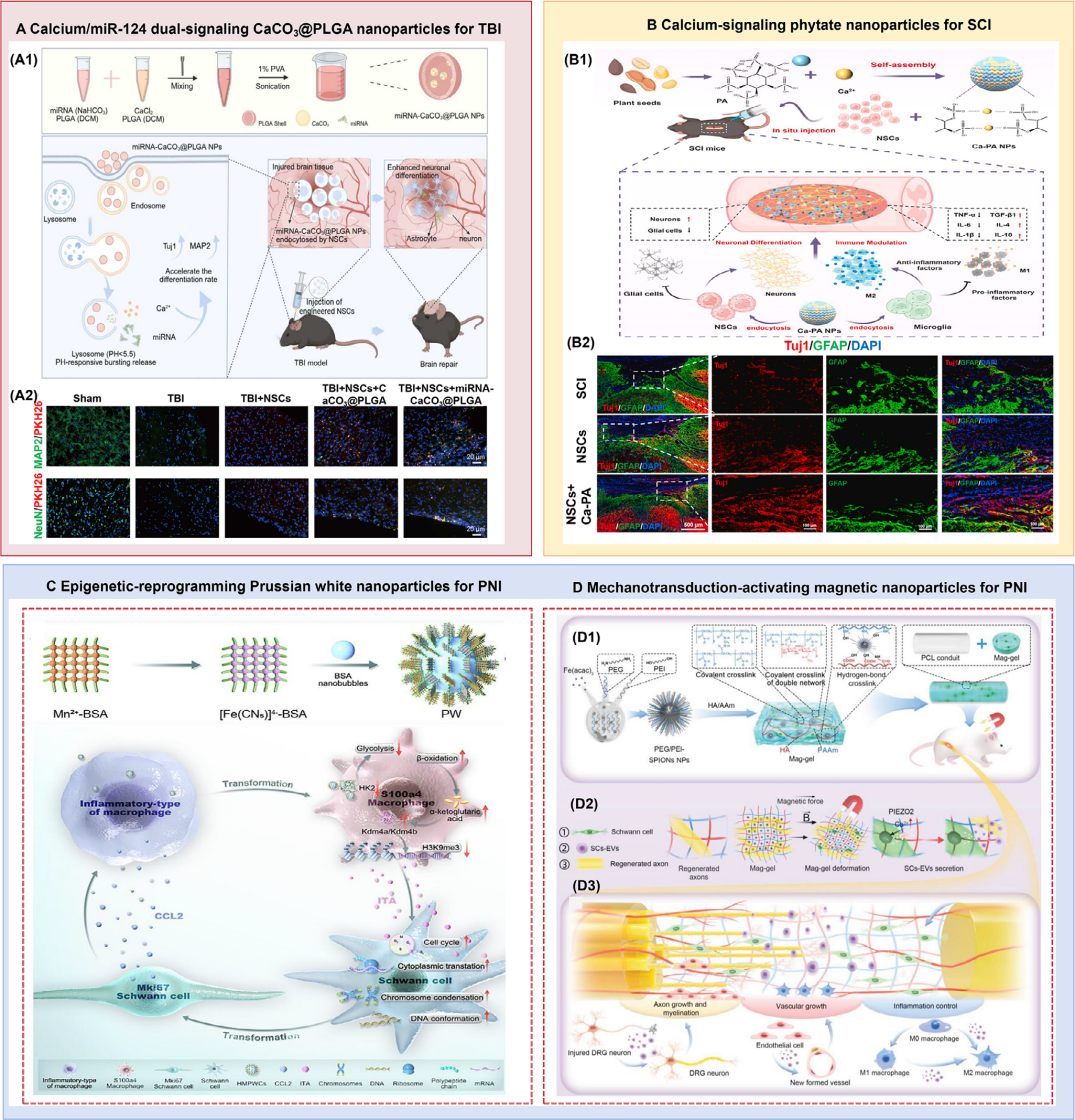
Paradigm VI: Intracellular signaling modulation and cell fate reprogramming. (A) Calcium/miR‐124 dual‐signaling CaCO_3_@PLGA nanoparticles for TBI. Reproduced with permission [203]. Copyright 2026, Elsevier Ltd. (A1) Schematic illustration of hierarchical miRNA‐CaCO_3_@PLGA nanoparticle assembly. Upon NSC uptake, pH‐dependent lysosomal CaCO_3_ degradation enables co‐release of Ca^2+^ and miR‐124 for dual neurogenic effects. Transplanted NSCs containing these nanoparticles promoted neuronal differentiation and cognitive recovery in TBI mice. (A2) IF imaging of NeuN and MAP2 showing a greater number of functional neurons in the miRNA‐CaCO_3_@PLGA nanoparticle‐treated group. (B) Calcium‐signaling phytate nanoparticles for SCI. Reproduced with permission [206]. Copyright 2025, Elsevier Ltd. (B1) Schematic illustration of the self‐assembly of Ca‐PA nanoparticles and their mechanism of action at the injury site. (B2) IF imaging of Tuj1 (red) / GFAP (green) showing enhanced neuronal regeneration and reduced astroglial scarring in the NSCs + Ca‐PA nanoparticle‐treated group. (C) Epigenetic‐reprogramming Prussian white nanoparticles for PNI. Reproduced from [208] under the terms of the CC BY 4.0. Copyright 2026, The Author(s). Schematic representation of the proposed mechanism: PW nanoparticles facilitate sustained local retention and drive coordinated immunometabolic‐epigenetic reprogramming, enhancing Schwann cell‐macrophage crosstalk to promote nerve repair. (D) Mechanotransduction‐activating magnetic nanoparticles for PNI. Reproduced from [210] under the terms of the CC BY 4.0. Copyright 2023, The Authors. (D1) Schematic showing the fabrication of Mag‐gel and construction of PCL nerve conduits for in vivo evaluation in PNI. (D2) Schematic illustration demonstrating magnetically triggered hydrogel deformation and mechanotransduction‐related cellular events. (D3) Schematic showing RMF‐inspired SCs‐EVs‐enhanced magnetic composite conduits for improved nerve repair through promoting axonal growth, neuroplasticity, and inflammation control.

SCI encompasses interconnected pathological processes, including neuroinflammation and impaired differentiation, which converge on intracellular signaling hubs. Zein nanoparticles co‐encapsulating celastrol, metformin, and everolimus promoted autophagy‐related signaling in microglia and neuron‐like cells, reduced inflammatory cytokine production, and improved histological and functional outcomes after SCI [205]. Beyond combinatorial drug delivery, calcium phytate nanoparticles undergo lysosomal decomposition, generating intracellular ion fluxes that promote neuronal differentiation of neural stem cells while driving reparative immune polarization through Ca^2+^‐dependent signaling cascades, significantly enhancing functional recovery (Figure 9B) [206]. An alternative mechanism leverages iron oxide nanoparticles, which elevate intracellular Fe^3+^ concentrations upon lysosomal degradation, promoting directed motor neuron differentiation via iron‐responsive transcriptional programs. This approach achieves 29.38% differentiation five days earlier than spontaneous differentiation and significantly improves motor and sensory recovery in SCI mice [207].

Peripheral nerve restoration depends critically on Schwann cell proliferation, migration, and remyelination, processes governed by intracellular cascades that can be exploited through nanoparticle‐mediated reprogramming of both Schwann cells and macrophages. Biomimetic Prussian white nanoparticles induce intracellular α‐ketoglutarate accumulation in macrophages, promoting histone H3 lysine 9 trimethylation (H3K9me3) demethylation at the S100a4 locus and activating a novel S100a4^+^ macrophage substate that secretes itaconate to support Schwann cell proliferation under inflammatory stress (Figure 9C) [208]. This immunometabolic–epigenetic mechanism enhanced structural and functional regeneration in rodent and canine sciatic nerve injury models. For direct Schwann cell modulation, chiral Fe_3_O_4_ nanoparticles entered Schwann cells and activated autophagy‐related phosphorylated c‐Jun N‐terminal kinase (p‐JNK)/ephrin type‐A receptor 5 (EPHA5) signaling, promoting proliferation, migration, and remyelinating differentiation and improving peripheral nerve repair [209]. Superparamagnetic nanocomposite scaffolds, as a complementary physical modality, generate micron‐ and nanoscale forces on embedded Schwann cells under rotating magnetic fields, activating mechanotransduction pathways, increasing the production and pro‐regenerative cargo of extracellular vesicles, and enhancing nerve regeneration in vivo (Figure 9D) [210].

Intracellular signaling modulation confers a fundamental advantage across all injury types: direct molecular intervention that alters cell fate rather than merely providing permissive conditions. TBI benefits from dual calcium‐ion and miRNA‐mediated neurogenesis; SCI leverages combinatorial drug delivery, ion flux‐mediated polarization, and iron‐driven differentiation; PNI exploits macrophage reprogramming, chiral nanoparticle‐mediated activation, and mechanotransductive exosome production. The common principle resides in harnessing the endocytic pathway to deliver functional payloads directly to intracellular targets, achieving therapeutic effects unattainable by extracellular drug delivery alone.

### 4.8 Cross‐Injury Commonalities and Modular Design Philosophy

Despite distinct anatomical and pathological profiles across TBI, SCI, and PNI, the six functional paradigms constitute a unified therapeutic toolbox amenable to all three injury categories through context‐dependent prioritization and combinatorial deployment. TBI preferentially engages paradigms that address diffuse parenchymal damage and barrier traversal, emphasizing biomimetic targeting and endogenous‐responsive release for spatiotemporal modulation of biphasic pathology. SCI mandates integration of scaffold‐based filling of cystic cavities, topographical guidance for white matter tract reconstruction, and intracellular reprogramming for immunomodulation and neuronal differentiation. PNI centers on conduit‐mediated mechanical guidance, sustained trophic support, and Schwann cell reprogramming for remyelination. The underlying logic and functional definitions of each paradigm remain invariant across injury types, enabling knowledge transfer and cross‐injury translation (Table 6).

**TABLE 6:** Functional design paradigms of nanomedicine across traumatic neural injuries.

Functional paradigm	Core therapeutic function	Key material/design requirements	TBI	SCI	PNI	Representative refs.
**I. Scaffold construction and microenvironment remodeling**	Fill tissue defects and provide a provisional ECM while locally modulating inflammation, oxidative stress, trophic signaling and vascularization	Biocompatibility; tissue‐matched mechanics; injectability or conformal filling; controlled degradation; sustained/sequential cargo release	**Moderate–high**: particularly valuable for focal lesions and intraoperative local delivery; temporal programming is important	**High**: cavity filling combined with antioxidant, immunomodulatory and regenerative support	**High**: conduit‐based bridging with sustained trophic and angiogenic support	TBI [157, 158]; SCI [159, 160, 161]; PNI [162, 163, 164, 165, 166]
**II. Topographical guidance and contact‐mediated regeneration**	Provide anisotropic physical cues to orient cell migration and axonal extension and re‐establish directional continuity	Stable alignment; anisotropic micro/nanostructure; appropriate stiffness; contact guidance; compatibility with hydrogels/conduits and, where applicable, magnetic alignment	**Context‐dependent**: useful in localized cortical defects but less applicable to diffuse TBI	**High**: ordered white‐matter architecture and lesion gaps create a strong need for directional guidance	**High**: aligned conduits can mimic Büngner‐band‐like longitudinal guidance	TBI [149]; SCI [168, 169]; PNI [170, 171, 172, 173, 174]
**III. Biomimetic camouflage and active targeted delivery**	Enhance circulation, immune evasion, BBB/BSCB traversal, lesion homing and cell‐selective delivery	Membrane coating or targeting ligands; preserved ligand accessibility; colloidal stability; controlled surface chemistry; reproducible biointerface	**High**: addresses heterogeneous BBB permeability and enables lesion/cell‐specific targeting	**High**: facilitates inflammatory homing, BSCB traversal and selective targeting of microglia/macrophages/astrocytes	**Moderate**: barrier‐crossing demand is lower; cell‐selective delivery remains attractive, but direct evidence is less developed in the current manuscript	TBI [97, 176, 177, 178, 179, 180]; SCI [95, 181, 182, 183, 184]
**IV. Endogenous microenvironment‐responsive release**	Convert pathological cues into triggers for localized, on‐demand therapeutic activation	Responsiveness to ROS, acidic pH, MMPs, ferroptotic/iron stress or other lesion signals; low basal leakage; sufficient trigger sensitivity and selectivity	**High**: ROS, acidosis and inflammatory signals provide strong triggers for adaptive release	**High**: ROS, pH and iron/ferroptotic dysregulation enable multimodal responsive intervention	**High**: particularly suitable for oxidative/ferroptotic stress and age‐related Schwann‐cell dysfunction	TBI [185] [186]; SCI [187, 188, 189, 211]; PNI [190, 191, 192]
**V. Exogenous physical‐field‐responsive intervention**	Convert externally applied magnetic, ultrasonic or optical energy into localized mechanical/electrical/photonic therapeutic signals	High energy‐conversion efficiency; controllable stimulation intensity; deep‐tissue activation; repeatability; integration with responsive/conductive materials	**Moderate–high**: magnetic/mechanotransductive and magnetoelectric stimulation can promote neurogenesis	**High**: magnetic navigation, ultrasound/piezoelectric stimulation and UCNP‐assisted optogenetic rewiring enable structural and electrophysiological intervention	**High**: ultrasound‐responsive and bioelectronic conduits enable wireless stimulation and controlled regeneration	TBI [194, 195]; SCI [196, 197, 198]; PNI [199, 200, 201, 202]
**VI. Intracellular signaling modulation and cell‐fate reprogramming**	Directly alter intracellular signaling, metabolism, epigenetic state and cell fate after nanoparticle internalization	Efficient cellular uptake; intracellular/lysosomal release; ion, nucleic‐acid or small‐molecule delivery; pathway specificity; controlled intracellular dose	**High**: promotes neural stem‐cell differentiation and metabolic/immune modulation	**High**: enables microglial reprogramming, neuronal differentiation and coordinated pathway modulation	**High**: reprograms macrophages and Schwann cells to promote proliferation, migration and remyelination	TBI [203, 204]; SCI [205, 206, 207]; PNI [208, 209, 210]

Therapeutic deployment rarely relies on a solitary paradigm; effective neural restoration generally requires sequential or simultaneous integration of multiple paradigms calibrated to evolving pathological demands. A hierarchical liposomal platform for TBI combines active BBB penetration through neutrophil hitchhiking and endothelial camouflage (biomimetic targeting) with MMP‐responsive release (microenvironment‐responsive release) and secondary neuron‐specific targeting via Tet1 peptide exposure (active targeting) [179]. For SCI, a neurotargeted ROS‐responsive nanodrug (Qu@NRNPs) couples active BSCB penetration through peptide‐mediated targeting (biomimetic targeting) with ROS‐triggered release (microenvironment‐responsive release), delivering quercetin to scavenge ROS and inhibit ferroptosis, thereby promoting functional restoration [211]. For PNI, composite nerve conduits incorporating Ga‐PTA nanoparticles and MSCs achieve competitive inhibition of ferroptosis through microenvironment‐responsive Ga^3+^ release (microenvironment‐responsive release), while stem cell differentiation into Schwann‐like cells bypasses the redifferentiation impairment of senescent Schwann cells (intracellular signaling modulation) [191]. These exemplars demonstrate that integration of complementary paradigms may address multiple injury‐specific barriers within a single platform.

Temporal progression of injury pathology further imposes phase‐dependent paradigm selection. During the hyperacute phase (0 to 6 h), biomimetic targeting delivers emergency anti‐inflammatory agents while microenvironment‐responsive nanozymes provide immediate ROS neutralization. Throughout the acute phase (6 to 72 h), responsive release sustains anti‐inflammatory and anti‐cell‐death actions, whereas intracellular reprogramming steers microglial or macrophage polarization toward reparative phenotypes. The subacute phase (72 h to 2 weeks) prioritizes scaffold‐mediated filling of tissue defects and topographical guidance that provide physical cues for axonal traversal. In the chronic phase (beyond 2 weeks), intracellular reprogramming directs neuronal or Schwann cell fate modulation, supplemented by external field stimulation for functional maturation. This phase‐dependent matrix serves as an operational guide for paradigm selection and combination aligned with evolving pathophysiology, advancing beyond anatomy‐based classification toward a modular, pathology‐driven design philosophy.

A delivery‐system perspective further organizes these functional paradigms, particularly for SCI, where therapeutic efficacy depends on both the biological target and the capacity of the material platform to accommodate the structural and microenvironmental constraints of the injured spinal cord. Lipid‐based and polymeric nanoparticles afford considerable flexibility for drug delivery, surface functionalization, and controlled release, whereas inorganic and conductive nanomaterials contribute additional catalytic, magnetic, optical, or electrical functionalities [63, 67, 73, 77]. Biomimetic nanoparticles and extracellular vesicles engage endogenous biological interfaces to enhance lesion‐directed delivery and neuroimmune modulation [181, 182, 183], while nanoparticle/hydrogel or scaffold hybrids integrate local retention and sustained release with structural support for tissue reconstruction [111, 112, 153]. These complementary properties carry distinct limitations in biodistribution, biodegradation, long‐term biosafety, manufacturing reproducibility, and clinical scalability. Table 7 summarizes the major advantages, limitations, and suitable therapeutic applications of these delivery systems in SCI.

**TABLE 7:** Comparative characteristics and therapeutic applicability of major nanomaterial‐based delivery systems for SCI.

Delivery system	Major advantages in SCI	Key limitations and translational considerations	Suitable SCI applications	Representative studies
**Lipid‐based nanoparticles and liposomes**	High biocompatibility; versatile drug loading; flexible surface functionalization; systemic administration and lesion‐directed delivery	Physical instability and cargo leakage; relatively limited drug loading; lesion specificity often requires additional targeting; repeated PEG exposure may induce immune responses	Acute neuroprotection; anti‐inflammatory therapy; targeted drug delivery across the disrupted BSCB	bFGF‐loaded liposomes [63]; inflammation‐targeted lipid nanoparticles [183]
**Polymeric nanoparticles**	Tunable degradation and release kinetics; sustained delivery; flexible physicochemical and surface engineering	Acidic degradation products for PLGA; residual solvents; batch variability; limited loading of some hydrophilic biomacromolecules	Sustained anti‐inflammatory/neuroprotective delivery; programmed release; intracellular drug delivery	Galantamine‐loaded PLGA NPs [73]; zein‐based triple‐drug NPs [205]
**Inorganic nanoparticles and nanozymes**	Intrinsic catalytic, magnetic, optical, or ion‐regulating functions; high physicochemical stability; potential integration of therapy with imaging or physical stimulation	Slow or incomplete biodegradation for some compositions; long‐term accumulation and clearance concerns; composition‐dependent toxicity	ROS regulation; magnetic guidance; externally responsive treatment; intracellular ion‐mediated modulation	Au@polyphenol nanospheres [77]; HA–Se NPs [184]; Ca‐phytate NPs [206]; iron oxide NPs [207]
**Conductive/carbon‐based nanomaterials**	Electrical conductivity; mechanical reinforcement; coupling with endogenous or externally applied physical stimulation	Long‐term biodegradation and biosafety remain incompletely characterized; aggregation and inflammatory concerns; conductivity requires careful optimization	Electrical stimulation; axonal guidance; electroactive neural interfaces	Wireless electrical‐stimulation/Zn^2^^+^‐release system [151]; magnetic nanochain‐based anisotropic repair system [168]
**Biomimetic nanoparticles and extracellular vesicles**	Endogenous biointerfaces support immune evasion, inflammatory‐site homing, and cell‐specific recognition; EVs provide intrinsic regenerative and immunomodulatory cargo	Heterogeneous composition; limited yield; difficult standardization and potency assessment; scale‐up and storage challenges	Lesion‐targeted delivery; neuroimmune modulation; regenerative signaling	Neuron‐membrane‐coated nanoceria [94]; membrane‐coated ZnS NPs [181]; neutrophil‐membrane nanoplatform [182]
**Nanoparticle–hydrogel/scaffold hybrid systems**	Local retention; sustained or sequential release; lesion‐cavity filling; structural bridging; integration of biochemical and physical cues	Requires local implantation/injection; increased formulation and manufacturing complexity; sterilization and reproducibility challenges	Lesion‐cavity repair; local multimodal therapy; microenvironment remodeling; axonal regeneration	Adaptive NP–hydrogel [111]; nanoparticle‐assembled hydrogel [112]; Ce–Mn NP/NGF hydrogel [160]

## 5 Generational Evolution, Biomarker‐Guided Development, and Translation of Nanomedicine Strategies

Cross‐injury analysis of nanomaterial interventions across TBI, SCI, and PNI, viewed through pathological modules, material properties, and functional paradigms, reveals a critical disconnect: escalating functional complexity does not proportionally enhance clinical translatability. Neural nanomedicine has instead followed a discernible technological trajectory, from early systems prioritizing drug stability and lesion exposure, through platforms incorporating active targeting, microenvironment responsiveness, and externally controlled physical stimulation [150, 193], to emerging approaches that integrate multi‐omics and AI for precision design [212]. Each additional functional module, however, compounds the challenges of manufacturing scale‐up, batch reproducibility, in vivo behavior prediction, biosafety evaluation, and regulatory classification [213]. The next stage of neural nanomedicine must therefore move beyond maximizing functional modules and instead define an optimal degree of functional complexity that balances pathological necessity, therapeutic benefit, and translational burden.

Nanomedicine has progressively broadened the scope of therapeutic control over delivery, advancing from passive modulation of drug disposition to targeted and stimulus‐responsive regulation, and more recently to precision design informed by disease biology and patient heterogeneity. Early nanocarriers chiefly improved the stability, pharmacokinetics, and sustained release of therapeutic agents, with lesion accumulation relying largely on altered vascular or barrier permeability. Subsequent platforms incorporated molecular recognition, biomimetic interfaces, and endogenous or externally triggered responses, thereby achieving greater spatial and temporal control over therapeutic delivery. In parallel, single‐cell and multi‐omics profiling, together with computational and artificial intelligence‐assisted design, increasingly enables nanoparticle properties and therapeutic combinations to be tailored to heterogeneous biological barriers, molecular targets, and patient‐specific disease characteristics. Within this evolving design landscape, the following sections organize nanomedicine strategies according to their predominant mode of therapeutic control and design sophistication, tracing the transition from passive delivery to actively regulated and increasingly precision‐oriented systems.

### 5.1 Generational Evolution of Nanomedicine Strategies

#### 5.1.1 First Generation: Passive Encapsulation and Sustained Release

The foundational principle of first‐generation nanotherapeutics lies in physical drug encapsulation, whereby therapeutic molecules are sequestered within biodegradable polymeric matrices or liposomal carriers to prolong systemic circulation and achieve sustained release (Figure 10A). Representative carriers encompass PLGA microspheres, PEGylated liposomes, and albumin nanoparticles, with payloads ranging from neurotrophic factors and anti‐inflammatory small molecules to hormonal agents for neural restoration. Targeting relies predominantly on injury‐induced pathological hyperpermeability of the BBB or BSCB, permitting drug accumulation at lesional sites through an enhanced permeability and retention (EPR)‐like phenomenon [214]. This constitutes a quintessential passive targeting modality whose design philosophy extends the classical sustained‐release formulation paradigm, effectively refining nanocarriers as more sophisticated drug depot systems.

**FIGURE 10:**
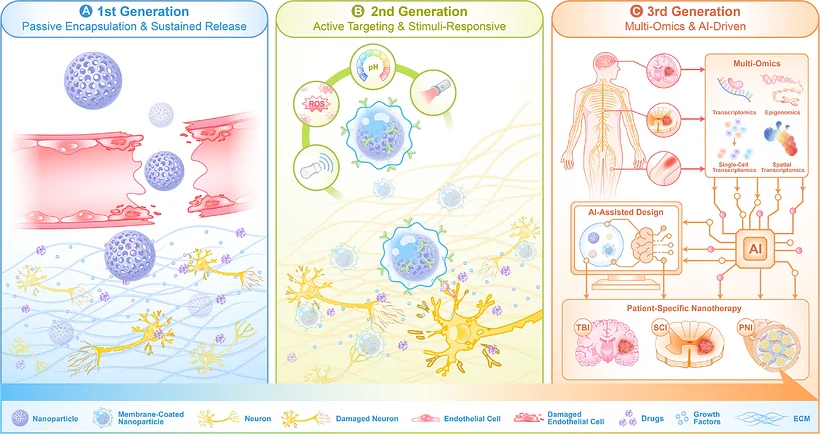
Generational evolution of nanomedicine strategies. Schematic illustration depicting the generational progression from First Generation: Passive Encapsulation and Sustained Release, to Second Generation: Active Targeting and Stimuli‐Responsive Systems, and to Third Generation: Multi‐Omics Integration, AI‐Assisted Design, and Personalized Customization. The schematic illustration was created using Adobe Illustrator 2025.

Nevertheless, first‐generation strategies are constrained by inherent limitations that resist facile resolution. The absence of active targeting produces predominant systemic distribution, with only marginal augmentation of local drug concentration at injury sites. Drug release typically conforms to a fixed biphasic profile of initial burst followed by sustained elution, which cannot accommodate the temporal demands of secondary neural injury spanning the acute inflammatory, subacute restoration, and chronic remodeling phases. Passive nanoparticle accumulation is further attenuated in mild injuries characterized by limited barrier leakage and becomes progressively unreliable as barrier permeability normalizes. More fundamentally, this strategy reduces complex neural injury to a static pharmacokinetic challenge, disregarding the dynamic pathological features of multi‐pathway activation and multi‐cellular involvement. These conceptual inadequacies of the first‐generation framework directly catalyzed the development of second‐generation nanoplatforms incorporating active targeting and programmable drug release.

#### 5.1.2 Second Generation: Active Targeting and Stimuli‐Responsive Systems

The seminal advance of second‐generation nanosystems lies in the integration of active targeting navigation, controlled release, and increasingly sophisticated stimulus‐responsive regulation (Figure 10B). Surface functionalization with targeting ligands confers precise recognition of the injury microenvironment: Arg‐Gly‐Asp (RGD) peptides engage injury‐associated integrins; CAQK peptides preferentially bind extracellular matrix components enriched in damaged brain tissue [215]; mannose modification enables selective targeting of CD206‐expressing macrophages or microglia within inflammatory lesions [183]. Cell membrane biomimetic camouflage offers an additional active‐targeting strategy, whereby nanoparticles coated with macrophage, neutrophil, or stem cell‐derived membranes inherit selected surface properties of their parent cells, enhancing lesion accumulation while reducing nonspecific clearance and off‐target distribution [97, 216].

Controlled release has been achieved through core‐shell architectures, compositional modulation, blending of polymers with disparate degradation kinetics, and self‐assembly of amphiphilic block copolymers. These engineering strategies permit sequential release profiles that align distinct therapeutic agents with specific pathological stages, coordinating early neuroprotective or anti‐inflammatory intervention with subsequent regenerative support [217]. Nevertheless, predetermined release programs cannot adapt to real‐time fluctuations in reactive oxygen species concentration, local pH, or matrix metalloproteinase activity within the injury microenvironment. This limitation has driven the development of stimulus‐responsive nanosystems, extending second‐generation platforms from programmed control toward adaptive regulation.

Within the endogenous response module, pathological microenvironment‐sensitive chemical bonds or linkers have been incorporated into nanocarriers. Thioketal or boronate ester structures mediate ROS‐responsive degradation, while hydrazone or acetal bonds provide pH‐sensitive release; protease‐cleavable peptide linkers, particularly those susceptible to matrix metalloproteinases, further enable drug release or structural transformation in response to injury‐associated enzymatic activity [218]. These responsive architectures remain relatively stable under physiological conditions yet undergo chemical or structural alterations within pathological environments, triggering nanoparticle disassembly, ligand exposure, or on‐demand drug release. In parallel, exogenous field‐responsive systems achieve therapeutic activation through externally applied physical stimuli. Magnetoelectric nanoparticles convert magnetic stimulation into local electrical signals and have been explored for promoting neural differentiation and restoration [219]. Ultrasound‐responsive piezoelectric materials provide an additional wireless electrical‐stimulation strategy, while upconversion nanoparticles convert near‐infrared irradiation into locally effective optical signals for remotely controlled therapeutic activation [81, 220].

Despite these advances, important limitations persist. Existing endogenous‐responsive systems predominantly address reactive oxygen species, pH, and protease‐associated signals, whereas pathological cues such as hypoxia, ATP depletion, and broader metabolic disturbances remain comparatively underexplored. Multi‐signal responsive architectures also increase design complexity and may introduce interference among functional modules. For exogenous systems, magnetoelectric conversion efficiency, piezoelectric or electrical output, and effective energy delivery to deep neural tissues remain significant engineering constraints [195]. More fundamentally, target selection in these systems still often relies on previously characterized therapeutic pathways rather than systematic data‐driven discovery, thereby motivating the transition toward multi‐omics‐guided and personalized nanoplatforms.

#### 5.1.3 Third Generation: Multi‐Omics Integration, AI‐Assisted Design, and Personalized Customization

A shared limitation of the preceding three generations is their conception as generic platforms rather than patient‐stratified therapeutic systems. Single‐cell transcriptomic and spatial analyses across TBI, SCI, and PNI have nonetheless exposed pronounced cellular, spatial, temporal, sex‐dependent, and model‐dependent heterogeneity, encompassing divergent immune activation states, glial responses, neuronal injury programs, and vascular or stromal alterations [221, 222, 223].

Third‐generation nanoplatforms distinguish themselves through a transition from empirical design to multi‐omics‐informed precision engineering (Figure 10C). At the target‐discovery level, single‐cell and multi‐omics technologies assemble comprehensive maps of dynamic transcriptional and molecular landscapes within the injured neural microenvironment, revealing stage‐ and cell‐state‐specific signaling networks that furnish a biological blueprint for multi‐target and network‐level nanotherapy design [221, 222, 223]. This strategy has already received preliminary validation. In PNI, integrated multi‐omics profiling identified a previously unrecognized S100a4+ macrophage substate, guiding the development of Prussian White nanoparticles that promote a pro‐regenerative macrophage program via α‐ketoglutarate accumulation and Kdm4a/b‐mediated epigenetic remodeling [208]. In SCI, P2Y12R has been exploited as a mechanistically informed microglial target to construct a dual‐targeting nanosystem that enhances microglia‐neuron interactions and neuronal mitophagy, demonstrating the feasibility of mechanism‐guided nanomedicine innovation [224].

Material development has likewise benefited from machine learning algorithms that capture high‐dimensional correlations among material composition, fabrication parameters, physicochemical properties, and biological performance, enabling efficient optimization of nanoparticle formulations and high‐throughput exploration of formulation space [225]. At the clinical application stage, biomarker‐based patient stratification could match appropriate nanocarriers, drug combinations, and response modalities to distinct patient subgroups. Multi‐omics and machine‐learning frameworks have already demonstrated the feasibility of data‐driven patient stratification in other highly heterogeneous diseases [226]. Quantitative imaging parameters such as K^trans^ derived from dynamic contrast‐enhanced MRI enable characterization of individual BBB permeability dynamics and may eventually support personalized optimization of administration timing and dosage [227].

Personalized fourth‐generation nanoplatforms currently remain largely in proof‐of‐concept and early‐stage development, with large‐scale clinical implementation yet to be realized. Nevertheless, this technological framework addresses critical limitations of generic therapies and represents an emerging direction in neurotrauma nanotherapy, progressing from standardized intervention toward precision and personalized targeted treatment.

### 5.2 Critical Bottlenecks in Clinical Translation

Despite pronounced therapeutic promise in preclinical models, the clinical translation of nanomedicine for traumatic neural injury remains constrained by multiple barriers. Regulatory approvals now exceed 50 nanomedicines across disease areas, with over 100 candidates under clinical evaluation, yet neurological applications remain comparatively limited [20, 228]. This gap is especially consequential for TBI, SCI, and PNI, where nanotherapeutics must satisfy general requirements of manufacturing reproducibility, pharmacokinetic control, and long‐term safety while also contending with dynamic BBB/BSCB permeability, intricate neuroimmune microenvironments, and marked heterogeneity of therapeutic windows across injury phases.

Translational readiness, however, remains uneven across traumatic neural injuries and reflects both injury context and platform class. Within PNI, biomaterial‐based repair has advanced comparatively far toward clinical implementation, with several NGCs and nerve wraps already cleared or approved for clinical use and supported by human studies [229]. More sophisticated nanoparticle‐integrated NGCs, including polymeric, magnetic, silica‐based, and lipid‐based systems for controlled neurotrophic factor delivery, nevertheless remain predominantly preclinical [230]. SCI exhibits a comparable profile for nanoparticle‐based therapeutics, which remain largely preclinical despite extensive experimental investigation of nanocarriers and regenerative nanomaterials [231]. The closest material‐based clinical precedent is the bioresorbable Neuro‐Spinal Scaffold (NSS), which has undergone human evaluation in the INSPIRE study (NCT02138110) and the subsequent randomized INSPIRE 2.0 trial (NCT03762655) [232, 233]. Notably, INSPIRE 2.0 reported ASIA Impairment Scale (AIS) grade improvement at 6 months in 2 of 10 NSS recipients and 3 of 10 controls, and the prespecified primary efficacy criterion was not met. TBI presents a distinct translational profile: although numerous conventional pharmacological interventions have undergone clinical evaluation, nanoparticle‐based therapeutic strategies remain predominantly preclinical, with BBB penetration, insufficient lesion‐specific drug exposure, and spatiotemporal heterogeneity of secondary injury continuing to complicate translation [234]. Across these distinct landscapes, several barriers recur, including scalable and reproducible manufacturing, quantitative characterization of biodistribution and clearance, biocompatibility and long‐term safety, reproducibility of preclinical efficacy in clinically relevant models, and regulatory complexity [20]. These shared challenges are discussed below.

#### 5.2.1 Scalable Manufacturing and Quality Control

The primary obstacle to clinical translation lies in converting complex laboratory‐scale nanoparticle fabrication into a scalable, reproducible, and pharmaceutically acceptable manufacturing process. Laboratory investigations customarily operate at milligram‐to‐gram scales, whereas clinical development demands substantial increases in production volume while preserving stable product attributes. For nanomedicines, particle size and distribution, morphology, surface charge, drug loading and encapsulation efficiency, ligand density, aggregation state, degradation kinetics, and storage stability collectively govern circulation, biodistribution, cellular uptake, and therapeutic efficacy. Inter‐batch variability therefore translates directly into discrepancies in safety and efficacy. These formulation‐ and biology‐associated properties constitute critical quality attributes (CQAs) that warrant identification and control within a quality by design (QbD) framework from early development [23, 235].

This challenge escalates as systems advance toward greater functional complexity. Second‐ and third‐generation platforms may simultaneously integrate targeting ligands, pathology‐responsive linkers, cell‐membrane coatings, nucleic‐acid payloads, or magnetoelectric and piezoelectric components, each imposing further demands on raw‐material control, assembly sequencing, stability, and reproducibility. Biomimetic carriers and extracellular vesicles present additional difficulties stemming from source‐cell variability, isolation, purification, cargo loading, and storage. Manufacturability therefore warrants consideration during candidate design rather than only after in vivo efficacy has been demonstrated. Escalating complexity does not inherently augment translational value; clinically viable systems must reconcile therapeutic functionality with manufacturability [20].

Universal acceptance thresholds for polydispersity index, drug loading, or ligand density should not be imposed uniformly across nanomedicines. Product‐specific CQA ranges should instead be delineated according to their demonstrated associations with mechanism of action and in vivo performance, and verified through fit‐for‐purpose orthogonal analytical methods. The central translational question resides in identifying which material attributes govern efficacy and safety, and whether these attributes can be reproducibly controlled during scale‐up.

#### 5.2.2 Translational Gap between Preclinical Efficacy and Safety

A further translational barrier arises from the substantial cross‐species disparities between preclinical models and human pathophysiology. Nanotherapeutic studies in TBI, SCI, and PNI have been predominantly restricted to murine systems, which, despite offering cost efficiency, genetic tractability, and experimental reproducibility, differ fundamentally from humans in neuroanatomical complexity, immune biology, metabolic rate, regenerative capacity, and dosing scale. For nanomedicines, such differences can further perturb hemodynamics, mononuclear phagocyte system clearance, protein corona formation, tissue penetration, and dose scaling, thereby compounding the inherent challenges of cross‐species translation [20].

Preclinical evidence synthesized in this review varies substantially in study design and evidentiary strength. Many investigations rest on a single rodent species, one injury severity, modest experimental groups, and abbreviated observation windows. Reporting remains inconsistent for randomization, blinded outcome assessment, sex as a biological variable, pharmacokinetics, and long‐term systemic safety. Biochemical or histological gains, moreover, do not necessarily translate into restored neural conduction, coordinated behavior, or clinically meaningful function. Even when behavioral improvements emerge, heterogeneity in injury models, scoring systems, treatment schedules, and comparators precludes direct quantitative comparison across nanoplatforms. Addressing these limitations will require preregistered, adequately powered studies, standardized multidomain outcome measures, independent replication, and validation in clinically relevant large‐animal models.

Large‐animal models represent an indispensable intermediate tier in the translational hierarchy. The porcine brain more closely approximates the human brain in size, gyrification, and white‐matter architecture, and rotational acceleration swine models faithfully recapitulate the biomechanical deformation and diffuse axonal pathology characteristic of clinical TBI [236]. Porcine SCI models have similarly been developed to assess motor, sensory, and autonomic outcomes in an anatomical setting that better mirrors the human spinal cord. The deficit in large‐animal validation is especially evident in PNI: a systematic analysis of 944 peripheral‐nerve transection studies found that approximately 91% employed rodents, while only 9% used large‐animal models [237]. Encouragingly, a 2026 study of Prussian White nanoparticles extended therapeutic validation from rodents to canine sciatic‐nerve injury, providing a recent example of a rodent‐to‐large‐animal translational bridge [238].

Beyond efficacy, species‐dependent nanoparticle‐biological interfaces and long‐term safety profiles raise additional translational concerns. Although PEGylation prolongs nanocarrier circulation, pre‐existing or treatment‐induced anti‐PEG antibodies can bind PEGylated liposomes and lipid nanoparticles, trigger complement activation, accelerate blood clearance, compromise carrier integrity, and cause premature cargo release [239]. These mechanisms have been experimentally confirmed across multiple PEGylated lipid systems using human plasma. Protein corona formation further dynamically reshapes nanoparticle biological identity; one study revealed that the corona acquired by magnetic nanoparticles during actual blood circulation differed markedly from coronas formed during conventional in vitro serum or plasma incubation, indicating that in vitro targeting results may incompletely predict in vivo behavior [208]. Preclinical development must therefore extend beyond short‐term efficacy to include organ retention, degradation products, immunogenicity, complement activation, repeated‐dose effects, and clearance pathways.

Human organoids and organ‐on‐chip platforms may serve as complementary bridges between conventional in vitro systems and animal models. A fully human iPSC‐derived BBB chip, incorporating brain microvascular endothelial‐like cells, astrocytes, and neurons, has recapitulated physiologically relevant barrier properties and predicted the blood‐to‐brain permeability of pharmacological agents [240]. At present, however, such platforms should be regarded as complementary screening and mechanistic tools rather than complete substitutes for systemic in vivo safety evaluation.

#### 5.2.3 Regulatory Pathway Refinement and Policy Challenges

A third bottleneck resides in the incomplete alignment between the intrinsic complexity of nanomedicines and conventional drug‐evaluation paradigms. Therapeutic behavior is governed not solely by the active payload but by a constellation of carrier‐specific variables, including composition, particle size, morphology, surface properties, aggregation state, protein corona, degradation behavior, and manufacturing processes. Two nanosystems bearing identical therapeutic agents may consequently exhibit divergent pharmacokinetics, biodistribution, and safety profiles. Regulatory‐science literature therefore underscores the necessity of a risk‐based, product‐specific framework that integrates physicochemical characterization, manufacturing control, in vivo behavior, and toxicological evaluation [241].

Nanomaterial characterization should accordingly pivot from maximizing the number of analytical assays toward CQA‐driven, fit‐for‐purpose evaluation. Particle size and distribution, morphology, surface properties, aggregation state, free‐to‐encapsulated drug ratio, release behavior, degradation, and biological identity may all prove relevant, yet the analytical panel should be tailored to product category and mechanism of action. More critically, developers must ascertain whether changes in these attributes materially alter clinically relevant performance. Risk‐based development provides a mechanism for concentrating resources on CQAs that truly govern product behavior [235, 241].

Hybrid nanoparticle‐biomaterial platforms employed in traumatic neural injury introduce further classification challenges. A single construct may integrate a pharmaceutical agent, nucleic acid, biologically derived membrane, implantable hydrogel or nerve conduit, and external magnetic or ultrasonic device, thereby straddling the domains of drugs, biological products, and medical devices. Development of such combination products requires early clarification of the principal mode of action, dominant safety risks, and manufacturing‐control boundaries. Jurisdictional divergence in nanomedicine definitions, characterization expectations, and development pathways additionally complicates international translation and commercialization [17].

Translational success for traumatic neural nanomedicines therefore hinges on establishing an early and continuous mapping across the target product profile, mechanism of action, CQAs, manufacturing controls, nonclinical safety package, and clinical endpoints. For BBB‐crossing systems, material attributes should be linked to brain exposure and target‐cell engagement, whereas implantable SCI or PNI nanoscaffolds must additionally address degradation, local tissue responses, neural regeneration, and device‐associated risks. Material innovation acquires clinical significance only when its complexity can be translated into measurable, controllable, and verifiable product attributes.

### 5.3 A Stepwise Translational Roadmap From Bench to Bedside

Given these translational hurdles, the clinical translation of nanomedicine for traumatic neural injury demands a staged validation framework with clearly defined phase‐specific objectives and go/no‐go decision points. Aligned with the overarching translational strategy, this pathway comprises six sequential phases: target discovery and material design, in vitro screening and optimization, proof‐of‐concept evaluation in small‐animal models, large‐animal validation, regulatory submission and clinical investigation, and post‐marketing surveillance. Each phase should generate quantitative evidence sufficient to justify progression; should critical criteria remain unmet, development ought to revert to material or therapeutic refinement rather than accumulating additional experimental complexity without clear purpose.

#### 5.3.1 Phase I: Target Discovery and Material Design

The principal objective of Phase I is to resolve two pivotal questions: which pathological process warrants therapeutic targeting, and which material function is indispensable for effective intervention. Single‐cell and spatial multi‐omics platforms, including single‐cell RNA sequencing (scRNA‐seq), single‐cell assay for transposase‐accessible chromatin sequencing (scATAC‐seq), spatial transcriptomics, proteomics, and metabolomics, enable the delineation of stage‐specific cell states, surface molecular signatures, metabolic bottlenecks, and intercellular signaling networks. In SCI, the integration of Ce‐doped upconversion nanozymes with snRNA‐seq and snATAC‐seq has demonstrated the capacity of multi‐omics to resolve treatment‐associated cellular and regulatory alterations [242]. Complementary transcriptomic bioinformatics analyses of SCI datasets have further nominated MYC as a ferroptosis‐related hub gene and potential therapeutic target, thereby illustrating how data‐driven screening can identify molecular targets prior to material selection [243]. In PNI, the integration of Prussian White nanoparticles with single‐cell, metabolic, and epigenetic analyses has showcased the capacity of multi‐omics to identify reparative macrophage states and therapeutic mechanisms [238].

Following target validation, material design must first ascertain whether the requisite function involves active delivery, ROS responsiveness, structural support, physical stimulation, or intracellular reprogramming, and only then select an appropriate lipid, polymeric, inorganic, biomimetic, or hybrid platform. AI and machine learning can assist by interrogating high‐dimensional formulation, property, and function relationships, yet they should complement rather than replace biological target validation. An exemplary application employed extreme gradient boosting (XGBoost) to identify manufacturing variables governing messenger RNA‐lipid nanoparticle (mRNA‐LNP) particle size and encapsulation, and subsequently leveraged Bayesian optimization to derive processing conditions for defined particle sizes [244]. Essential outputs include a validated pathological target or cell state, a preliminary target product profile, a candidate material architecture, a proposed mechanism of action, and predefined performance criteria for progression.

#### 5.3.2 Phase II: In Vitro Screening and Optimization

Phase II aims to establish quantitative relationships among material properties, cellular interactions, and therapeutic outcomes, thereby enabling the selection of a lead formulation from multiple candidates. Physicochemical characterization should cover particle size and distribution, morphology, surface properties, cargo loading efficiency, release kinetics, stability, and degradation profiles. Biological evaluation must simultaneously assess cellular uptake, intracellular trafficking and endosomal escape, target‐cell selectivity, inflammatory responses, neurotoxicity, and the intended mechanism of action. Microfluidic manufacturing, together with design of experiments or QbD‐driven optimization, should proceed in parallel so that production reproducibility is addressed before resource‐intensive animal studies. Investigations of PLGA and lipid nanoparticle manufacturing have shown that flow conditions, mixing technologies, and solvent parameters can markedly influence nanoparticle quality attributes [245].

Conventional two‐dimensional culture provides value for high‐throughput screening but fails to recapitulate the neurovascular unit, multicellular interactions, or physiological flow dynamics. Platforms designed for BBB or BSCB penetration therefore require assessment in human iPSC‐derived BBB‐Chips or analogous systems to probe barrier traversal and neurotoxicity [240]. For PNI and implantable SCI platforms, three‐dimensional neural tissues, Schwann cell‐axon cocultures, organoids, or nerve‐on‐chip configurations may offer enhanced physiological fidelity.

Protein corona formation must be incorporated into formulation evaluation. Because conventional in vitro serum or plasma incubation incompletely reproduces the in vivo corona, profiles generated under distinct human plasma, serum, or CSF conditions should be regarded as comparative screening parameters rather than definitive predictions of in vivo identity, which requires re‐evaluation at subsequent stages [208]. Essential outputs comprise a lead formulation, a robust manufacturing parameter window, an in vitro pharmacodynamic and safety profile, preliminary protein corona characterization, and predefined dosing and go/no‐go criteria for animal studies.

#### 5.3.3 Phase III: Proof‐of‐Concept in Small Animal Models

Phase III validation should establish whether the lead formulation engages its intended therapeutic cascade in vivo. Paradigm selection is dictated by the clinical question under investigation. TBI models encompass controlled cortical impact, fluid percussion, weight drop, and closed head injury; SCI is typically reproduced by contusion, compression, or transection; PNI can be modeled via crush, segmental defects, or chronic compression [246, 247].

Functional gains in motor scores, without corroborating evidence of delivery to the target tissue, subcellular compartment, or molecular effector, do not alone justify further translation [248]. A quantitative framework that integrates biodistribution, systemic and tissue pharmacokinetics, targeting efficiency, histopathology, pharmacodynamics, immune responses, and preliminary toxicology is therefore required, permitting exposure response or pharmacokinetic/pharmacodynamic (PK/PD) relationships to be resolved where possible [143].

Robustness should likewise be interrogated across sex, injury severity, and independently generated experimental cohorts. Efficacy confined to a narrow therapeutic window or to highly specific experimental conditions raises concerns regarding clinical feasibility that must be resolved before advancement. In vivo proof of concept, preliminary PK/PD relationships, target engagement evidence, safety signals, and a formal go/no‐go determination constitute the pivotal deliverables.

#### 5.3.4 Phase IV: Validation in Large Animal Models

Phase IV constitutes the pivotal translational boundary where preclinical efficacy must be reconciled with clinical viability. Large‐animal validation transcends confirmatory replication of rodent findings, interrogating variables imposed by human‐scale neuroanatomy, biomechanics, tissue dimensions, circulatory dynamics, and administration routes. Rotational acceleration models of TBI in swine recapitulate the biomechanical hallmarks of diffuse axonal injury within a gyrencephalic cortex [236], while porcine SCI models enable concurrent motor, sensory, and autonomic assessment at spinal cord dimensions approaching those of humans [249]. Canine and other large‐animal PNI models, which more faithfully reproduce extended axonal regeneration and clinically pertinent nerve gap reconstruction, have supported translational bridging from rodent to canine sciatic nerve injury [238].

Systematic re‐evaluation across these models must encompass pharmacokinetics, whole‐organ biodistribution, target tissue exposure, immunogenicity, therapeutic efficacy, and long‐term safety. Dosing regimens require calibration to resolve clinically meaningful exposure‐response relationships, and follow‐up intervals should be governed by material residence time and anticipated toxicity. Inorganic or carbon‐based constructs with slow degradation kinetics demand rigorous assessment of prolonged organ retention and neurotoxic potential, whereas rapidly degrading lipid or polymeric platforms necessitate detailed characterization of degradation byproducts and repeated‐dose immune responses.

Clinical advancement thus depends on feasible administration, predictable pharmacokinetic and biodistribution profiles, reproducible efficacy, and an acceptable therapeutic index. Pivotal deliverables include large‐animal PK/PD profiles, whole‐organ biodistribution, immunological and long‐term safety data, efficacy validation, and the comprehensive nonclinical dossier required for regulatory submission.

#### 5.3.5 Phase V: Regulatory Submission and Clinical Trials

Following successful completion of critical nonclinical validation, the candidate nanosystem advances to regulatory submission and initial clinical evaluation. The regulatory dossier must present a coherent continuum linking chemistry, manufacturing, and controls (CMC), nonclinical pharmacology and toxicology, and the clinical trial protocol. This continuum includes manufacturing processes, raw‐material controls, critical quality attributes (CQAs) and release specifications, analytical methods, stability data, batch consistency, pharmacokinetics, biodistribution, safety pharmacology, and toxicology. Nanomedicines that undergo process modifications or scale‐up during preclinical development also require comparability assessments to ensure that manufacturing changes have not altered in vivo performance [20].

Clinical development should be structured to reduce uncertainty incrementally. Early‐phase trials prioritize safety, tolerability, pharmacokinetics, and mechanism‐linked target‐engagement biomarkers; subsequent studies examine dose‐response relationships and preliminary efficacy before progression to adequately powered confirmatory or pivotal trials. Traumatic neural injury imposes additional demands for rigorous patient stratification, as TBI phenotype, SCI neurological level and completeness, and PNI nerve type, defect length, and timing of intervention can profoundly shape clinical outcomes. Imaging, blood and CSF biomarkers, and standardized neurological assessments should therefore be integrated into trial design.

Clinical experience with nanomedicines in traumatic neural injury remains limited, although other neurological nanomedicine trials provide methodological precedents. In the Phase II REPAIR‐MS and REPAIR‐PD studies of the gold nanocrystal suspension CNM‐Au8, 7‐Tesla phosphorus‐31 magnetic resonance spectroscopy (^31^P‐MRS) was employed to quantify brain NAD^+^/NADH, ATP‐related metabolites, and other energetic parameters as evidence of CNS target engagement [32]. These studies involved multiple sclerosis and Parkinson's disease and must not be interpreted as direct evidence of efficacy in TBI, SCI, or PNI. Their relevance lies in demonstrating how quantitative imaging and mechanistic biomarkers can establish whether a nanotherapeutic engages its intended biological pathway in the human CNS. Key deliverables include a regulatory‐ready CMC and nonclinical package, early human safety and PK/PD data, target‐engagement evidence, a rationale for dose selection, and clinical proof‐of‐concept supporting subsequent confirmatory development.

#### 5.3.6 Phase VI: Post‐Marketing Surveillance and Lifecycle Learning

Post‐marketing safety evaluation represents an enduring commitment rather than a terminal milestone following regulatory authorization. Clinical trials, constrained by limited sample sizes, selective enrollment criteria, and finite follow‐up durations, frequently fail to detect rare or delayed adverse events. Long‐term tissue retention, repeated‐dose immune responses, and chronic neurological sequelae of nanomaterials therefore necessitate dedicated real‐world surveillance. Registries, observational cohorts, pharmacovigilance frameworks, and real‐world evidence should collectively underpin continuous reassessment of the benefit‐risk balance.

Post‐marketing surveillance for traumatic neural nanomedicines warrants attention to prolonged hepatic, splenic, or neural retention of slowly degradable materials, alongside chronic neurotoxicity, neuroinflammation, and repeated‐dose immunogenicity linked to PEGylated or biomimetic systems. Surveillance frameworks should additionally capture degradation or mechanical failure of implantable nanoscaffolds, together with delayed consequences for neural plasticity, cognition, autonomic regulation, and sensory processing. For combination products that incorporate neural conduits, electrical‐stimulation devices, or other hardware, parallel tracking of device‐related and drug‐related adverse events becomes essential [250].

Post‐marketing surveillance should function not as a terminal step but as the initiation of a learning cycle. Real‐world clinical data can be re‐integrated with multi‐omics and AI frameworks to stratify responders and non‐responders, identify novel safety signals or therapeutic targets, and guide the redesign of material composition, payload combinations, and administration strategies.

### 5.4 Biomarker‐Guided Development and Evaluation of Nanotherapeutics

Biomarker integration should span the entire nanotherapeutic development continuum, extending beyond exploratory analyses confined to clinical trials. Along the translational trajectory, biomarkers can delineate injury phenotype and therapeutic window, verify delivery and biological target engagement, track pharmacodynamic response, and connect molecular alterations to clinically meaningful neurological outcomes. Prospective biomarker selection, aligned with the proposed mechanism of action, should therefore inform stage‐specific go/no‐go criteria.

TBI affords the most mature translational framework for this approach. Serial blood quantification of glial fibrillary acidic protein (GFAP) and ubiquitin C‐terminal hydrolase‐L1 (UCH‐L1) yields complementary information on acute astroglial and neuronal injury, respectively [251]. Neurofilament light chain (NfL) reflects axonal injury and remains informative during subacute and chronic phases, whereas total tau and phosphorylated tau provide complementary information on post‐traumatic cytoskeletal disruption and evolving tau‐related pathology [252, 253]. Beyond these established markers, neuroproteomic profiling may identify molecularly distinct injury phenotypes and support patient stratification for personalized neurotrauma evaluation [254]. Because these biomarkers and molecular signatures exhibit distinct temporal profiles, longitudinal trajectories generally prove more informative than isolated measurements.

Blood biomarker profiles merit joint interpretation with computed tomography or magnetic resonance imaging, including diffusion‐ or permeability‐sensitive measurements where appropriate [253]. These profiles should further be aligned with standardized neurological and functional outcomes, such as the Glasgow Coma Scale, Glasgow Outcome Scale‐Extended, and domain‐specific cognitive or motor assessments [255]. Such an integrated strategy may refine patient stratification and identify subgroups most likely to benefit from a given nanotherapeutic intervention.

For nanotherapeutics, evidence of delivery warrants clear distinction from evidence of biological activity. Preclinical investigations can employ labeled‐carrier tracking, tissue‐specific and cell‐specific biodistribution analysis, and quantification of released cargo to establish whether a formulation reaches its intended compartment [256]. Early clinical studies may integrate plasma or cerebrospinal fluid pharmacokinetics with quantitative imaging or formulation‐specific tracers where technically feasible [257]. Target engagement should then be evaluated through mechanism‐aligned readouts of oxidative stress, inflammation, regulated cell death, axonal injury, or other processes pertinent to the therapeutic payload.

Serial pharmacodynamic profiling delineates the onset, magnitude, and duration of biological response and informs exposure‐response or pharmacokinetic/pharmacodynamic modeling [258]. Such molecular changes merit integration with imaging, electrophysiological, behavioral, and patient‐centered functional outcomes, rather than serving as standalone evidence of neurological recovery. Analogous injury‐specific panels, coupled with appropriate imaging, electrophysiological, motor, sensory, and autonomic assessments, will be necessary for SCI and PNI [259].

Assay platforms, sampling schedules, preanalytical procedures, biological confounders, and decision thresholds merit prespecification and independent validation. A biomarker, until formally qualified as a surrogate endpoint, should complement clinically meaningful functional evaluation rather than displace it.

## 6 Conclusion and Future Perspectives

### 6.1 Core Summary

Traumatic injuries to the brain, spinal cord, and peripheral nerves impose a substantial and persistent burden on patients and healthcare systems worldwide. Despite decades of investigation, no broadly effective intervention has yet emerged that reliably attenuates the secondary‐injury cascade and restores lost neurological function across patient populations. A central principle emerges from the pathobiological synthesis presented in this review: although TBI, SCI, and PNI differ anatomically, they share a common repertoire of core pathological modules, each operating with distinct temporal dynamics, cellular effectors, and spatial constraints that create injury‐specific therapeutic bottlenecks requiring tailored intervention strategies.

This principle underpins the nanoparticle‐based framework advanced here. Nanoparticles provide a versatile means to address such multifaceted pathology through the concurrent integration of structural, signaling, and delivery functions. Six functional paradigms have been established to translate nanoparticle properties into therapeutic action, progressing from passive support to active intervention: scaffold construction, topographical guidance, biomimetic targeting, endogenous microenvironment‐responsive release, exogenous physical field modulation, and intracellular reprogramming. These strategies have evolved from single‐agent encapsulation to intelligent responsive systems and are now advancing into data‐driven personalized platforms, traversing two generations and entering a third. The three‐tier architecture of pathological modules, functional paradigms, and particle platforms furnishes a generalizable framework for integrated investigation and reproducible design across TBI, SCI, and PNI, thereby shifting the conceptual foundation from anatomy‐based classification toward pathology‐driven therapeutic design.

### 6.2 Future Priority Directions

Fundamental investigations must now achieve deeper integration of multi‐omics platforms, including single‐cell transcriptomics, epigenomics, spatial transcriptomics, and proteomics, with nanoparticle design [260]. Such integrative analyses should systematically identify cell‐type‐selective and pathway‐specific therapeutic targets that remain relevant across the evolving phases of neural injury, from oxidative stress and excitotoxicity to emerging regulated cell death modalities such as ferroptosis, necroptosis, and pyroptosis. The molecular insights derived from these efforts should subsequently inform the rational engineering of targeted nanotherapeutics capable of engaging these pathways with high specificity. In parallel, the molecular determinants of nano‐bio interactions warrant equally rigorous mechanistic interrogation, as therapeutic efficacy ultimately hinges on the behavior of nanoparticles at biological interfaces. Future research must therefore elucidate how protein corona composition evolves over time and dictates the selection of endocytic routes, how lysosomal escape efficiency can be systematically enhanced, and how mitochondrial targeting specificity can be achieved through deliberate nanocarrier architecture for subcellular therapeutic precision.

At the translational interface, systematic validation in large‐animal models, encompassing canine, porcine, and non‐human primate systems, should be prioritized to establish predictive cross‐species efficacy frameworks [261]. Patient‐derived organoid‐on‐a‐chip platforms that incorporate vascularization and immune cell co‐culture can bridge the persistent gap between in vitro screening and in vivo evaluation [262]. AI and data‐driven strategies increasingly propel nanoparticle development beyond empirical formulation screening toward computationally guided optimization. TuNa‐AI integrates automated experimentation with a hybrid kernel machine to learn relationships among drug molecules, excipients, formulation ratios, and nanoparticle formation, demonstrating how machine learning can guide compositional optimization of tunable drug‐delivery nanoparticles [16]. Machine‐learning strategies have likewise been deployed at the manufacturing level. A separate investigation modeled relationships between lipid nanoparticle (LNP) manufacturing parameters and critical quality attributes, including particle size and mRNA encapsulation efficiency, illustrating the potential of data‐driven methods to support process understanding and optimization [242]. At the biological level, computationally guided material development can be complemented by high‐dimensional multi‐omics profiling. In SCI, a further study combined single‐nucleus RNA sequencing (snRNA‐seq) and single‐nucleus ATAC sequencing (snATAC‐seq) to characterize cell‐type‐specific transcriptional and chromatin‐accessibility changes following treatment with Ce‐doped upconversion antioxidant nanoenzymes. That analysis revealed increased representation of myelinating oligodendrocytes and regulatory programs associated with remyelination [17]. Collectively, these investigations outline a prospective closed‐loop workflow linking multi‐omics‐guided target identification with machine‐learning‐assisted optimization of material composition and manufacturing parameters. Coupling with high‐throughput microfluidic screening could further accelerate this iterative design, synthesis, characterization, testing, and learning cycle [263].

Regulatory and industrial advancement necessitates adaptive reform of nanomedicine evaluation frameworks, including dedicated review pathways and standardized characterization systems. Sustained institutional dialogue among academic, industrial, and regulatory stakeholders should be maintained to formulate consensus guidelines governing the clinical translation of nanotherapeutics for neural restoration.

### 6.3 The Way Forward

The ultimate objective extends beyond symptomatic palliation toward the establishment of stratified, patient‐specific precision intervention. Integrating each patient's injury phenotype, pathological stage, genetic background, and individual biomarker signature would empower clinicians to rationally assemble the optimal combination of functional paradigms and nanoparticle platforms. Such a precision‐driven strategy promises meaningful functional restoration for the millions who sustain traumatic neural injury annually, thereby transforming neural restoration from an empirically managed practice into a rational, patient‐centered therapeutic discipline.

## Author Contributions

**Mouyuan Sun**: Writing – review & editing, Validation, Project administration, Investigation, Formal analysis, Conceptualization. **Shuangyang Li**: Writing – original draft, Resources, Methodology, Data curation. **Zhixu He**: Writing – original draft, Resources, Methodology, Investigation. **Jingyi Xie**: Data curation, Validation. **Yaxian Luo**: Methodology, Investigation. **Xuankai Fan**: Investigation. **Jingyu Zhang**: Formal analysis. **Luying Qin**: Methodology. **Lianjie Peng**: Software. **Tao Qiu**: Software. **Tian Zhang**: Conceptualization. **Huiming Wang**: Supervision, Conceptualization. **Mengfei Yu**: Writing – review & editing, Methodology, Investigation, Funding acquisition.

## Ethical Statement

No additional institutional ethical approval or approval number was required for the present work.

## Conflicts of Interest

The authors declare no conflicts of interest.

## Supporting information




**Supporting File**: advs78308‐sup‐0001‐SuppMat.docx.

## Data Availability

The data that support the findings of this study are available from the corresponding author upon reasonable request.
