# Supplementary material for: Precision Nanomedicine for Traumatic Neural Injury: Functional Paradigms and Translational Frameworks
Source: Adv Sci (Weinh). 2026 Oct 22:e78308. Online ahead of print. doi: 10.1002/advs.78308 (PMC13690977; doi:10.1002/advs.78308)
Supplement: Supplementary file 1 [file ADVS-9999-e78308-s001.docx]

**Supplementary File of**

**Precision Nanomedicine for Traumatic Neural Injury: Functional Paradigms and Translational Frameworks**

**Mouyuan Sun^1#*^, Shuangyang Li^1#^, Zhixu He^1#^, Jingyi Xie^1^, Yaxian Luo^1^, Xuankai Fan^1^, Jingyu Zhang^1^, Lianjie Peng^1^, Luying Qin^1^, Tao Qiu^1^, Tian Zhang^1^, Huiming Wang^1^, Mengfei Yu^1*^**

1. Stomatology Hospital, School of Stomatology, Zhejiang University School of Medicine, Zhejiang Provincial Clinical Research Center for Oral Diseases, Zhejiang Key Laboratory of Oral Biomedical, China, Hangzhou, 310000

#. These authors contributed equally.

*. Corresponding authors.

*E-mail: sunmouyuan777@zju.edu.cn

*E-mail: yumengfei@zju.edu.cn

**Supplementary Methods**

**Supplementary Method 1 GBD 2021 Data Retrieval and Descriptive Analysis**

Population-level estimates were obtained from the Global Burden of Disease Study 2021 results made available by the Institute for Health Metrics and Evaluation and were downloaded in August 2026. For TBI and SCI, the extracted variables comprised measure, year, age, sex, location, and the corresponding point estimates and uncertainty bounds. The measures used in the present analysis included prevalence, years lived with disability (YLDs), and disability-adjusted life years (DALYs), with the analysis restricted to 2021. Estimates for both sexes and sex-specific estimates were retained. Global data were used for the overall neurological-condition ranking and the age- and sex-stratified analyses, whereas estimates from the 21 GBD regions were used for the regional comparison. PNI was not represented as a separate condition within the corresponding GBD neurological classification and was therefore not included in the GBD-based panels.

In the analytical files used for Figure 1A-D, the mean, lower, and upper rate estimates were stored as population proportions and were converted to rates per 100,000 population by multiplying each value by 100,000. All rates presented in Figure 1A-D are therefore expressed per 100,000 population, and no conversion to rates per 1,000 population was performed. For Figure 1A, neurological conditions were ranked according to their global age-standardized DALY rates, with TBI and SCI highlighted within the resulting distribution. For Figure 1B, both-sex age-specific DALY rates for TBI and SCI were summarized across five prespecified age groups: younger than 5 years, 5-19 years, 20-59 years, 60-79 years, and 80 years or older. Figure 1C presents age-standardized prevalence, YLD, and DALY rates for both sexes, males, and females. For Figure 1D, both-sex age-standardized DALY rates were compared across the 21 GBD regions and independently ordered from highest to lowest for TBI and SCI. For visualization, regional estimates above the 75th percentile were categorized as high burden, those between the 25th and 75th percentiles as medium burden, and those at or below the 25th percentile as low burden; these categories were used only for graphical color coding and were not treated as inferential thresholds. Point estimates were rounded to one decimal place for graphical labeling, whereas ordering and calculations were performed using the unrounded values. The corresponding 95% uncertainty intervals were retained and displayed for the age-specific and regional estimates. The analysis was descriptive, and no hypothesis testing, temporal trend modeling, or causal inference was performed.

**Supplementary Method 2 Targeted Quantitative Synthesis of Clinical Outcomes for Existing Treatments in TBI, SCI, and PNI**

**Literature Search Strategy**

A focused systematic search was conducted in PubMed, Web of Science Core Collection, and Embase from database inception to August 15, 2026. Separate strategies were developed for TBI, SCI, and PNI because the clinically relevant interventions and outcomes differ across these injury categories. The search was designed specifically to support the targeted quantitative synthesis in Figure 1E-G and was not used to define the broader narrative nanomedicine literature included elsewhere in the review. The complete database-specific queries are reproduced below.

**TBI**

**Web of Science Core Collection**

TS=(("traumatic brain injur*" OR "head injur*") AND (pharmacotherap* OR neuroprotect* OR progesterone OR nimodipine OR erythropoietin OR epoetin OR citicoline OR selfotel OR methylprednisolone) AND (random* OR placebo* OR trial*)) AND PY=(1960-2026)

**PubMed**

( "Brain Injuries, Traumatic"[Mesh] OR "traumatic brain injury"[Title/Abstract] OR "traumatic brain injuries"[Title/Abstract] OR "head injury"[Title/Abstract] OR "head injuries"[Title/Abstract] OR TBI[Title/Abstract] ) AND ( "Drug Therapy"[Mesh] OR pharmacotherap*[Title/Abstract] OR neuroprotect*[Title/Abstract] OR progesterone[Title/Abstract] OR nimodipine[Title/Abstract] OR erythropoietin[Title/Abstract] OR epoetin[Title/Abstract] OR EPO[Title/Abstract] OR citicoline[Title/Abstract] OR selfotel[Title/Abstract] OR "CGS 19755"[Title/Abstract] OR methylprednisolone[Title/Abstract] OR corticosteroid*[Title/Abstract] ) AND ( randomized controlled trial[Publication Type] OR controlled clinical trial[Publication Type] OR random*[Title/Abstract] OR placebo[Title/Abstract] OR trial[Title/Abstract] ) AND ( "1960/01/01"[Date - Publication] : "2026/08/15"[Date - Publication] )

**Embase**

('traumatic brain injury'/exp OR 'traumatic brain injury':ti,ab,kw OR 'traumatic brain injuries':ti,ab,kw OR 'head injury':ti,ab,kw OR 'head injuries':ti,ab,kw OR tbi:ti,ab,kw) AND ('drug therapy'/exp OR pharmacotherap*:ti,ab,kw OR neuroprotect*:ti,ab,kw OR progesterone:ti,ab,kw OR nimodipine:ti,ab,kw OR erythropoietin:ti,ab,kw OR epoetin:ti,ab,kw OR epo:ti,ab,kw OR citicoline:ti,ab,kw OR selfotel:ti,ab,kw OR 'cgs 19755':ti,ab,kw OR methylprednisolone:ti,ab,kw OR corticosteroid*:ti,ab,kw) AND ('randomized controlled trial'/exp OR 'controlled clinical trial'/de OR random*:ti,ab,kw OR placebo*:ti,ab,kw OR trial*:ti,ab,kw) AND [01-01-1960 to 15-08-2026]/pd

**SCI**

**Web of Science Core Collection**

TS=( ("traumatic spinal cord injur*" OR "spinal cord trauma") AND (pharmacotherap* OR neuroprotect* OR GM1 OR ganglioside* OR riluzole OR "granulocyte colony stimulating factor" OR G-CSF OR "hepatocyte growth factor" OR HGF OR KP-100 OR VX-210 OR Cethrin) AND (random* OR placebo* OR trial*)) AND PY=(1960-2026)

**PubMed**

("Spinal Cord Injuries"[Mesh] OR "traumatic spinal cord injury"[Title/Abstract] OR "traumatic spinal cord injuries"[Title/Abstract] OR "spinal cord trauma"[Title/Abstract] OR SCI[Title/Abstract]) AND ( "Drug Therapy"[Mesh] OR pharmacotherap*[Title/Abstract] OR neuroprotect*[Title/Abstract] OR GM1[Title/Abstract] OR ganglioside*[Title/Abstract] OR riluzole[Title/Abstract] OR "granulocyte colony-stimulating factor"[Title/Abstract] OR "granulocyte-colony stimulating factor"[Title/Abstract] OR G-CSF[Title/Abstract] OR "hepatocyte growth factor"[Title/Abstract] OR HGF[Title/Abstract] OR "KP-100"[Title/Abstract] OR "VX-210"[Title/Abstract] OR Cethrin[Title/Abstract]) AND ( randomized controlled trial[Publication Type] OR controlled clinical trial[Publication Type] OR random*[Title/Abstract] OR placebo[Title/Abstract] OR trial[Title/Abstract]) AND ("1900/01/01"[Date - Publication] : "2026/08/15"[Date - Publication])

**Embase**

('spinal cord injury'/exp OR 'traumatic spinal cord injury':ti,ab,kw OR 'spinal cord trauma':ti,ab,kw) AND ('drug therapy'/exp OR pharmacotherap*:ti,ab,kw OR neuroprotect*:ti,ab,kw OR gm1:ti,ab,kw OR ganglioside*:ti,ab,kw OR riluzole:ti,ab,kw OR 'granulocyte colony stimulating factor':ti,ab,kw OR 'g-csf':ti,ab,kw OR 'hepatocyte growth factor':ti,ab,kw OR 'kp-100':ti,ab,kw OR 'vx-210':ti,ab,kw) AND ('randomized controlled trial'/exp OR 'controlled clinical trial'/exp OR random*:ti,ab,kw OR placebo*:ti,ab,kw OR trial*:ti,ab,kw) AND [01-01-1960 to 15-08-2026]/pd

**PNI**

**Web of Science Core Collection**

TS=(("peripheral nerve injur*" OR "digital nerve injur*" OR "nerve gap*" OR "nerve defect*") AND ("nerve repair" OR "nerve reconstruction" OR conduit* OR autograft* OR allograft* OR "nerve graft*" OR "processed nerve allograft" OR "vein graft*" OR neurorrhaphy) AND (compar* OR random* OR trial* OR cohort*)) AND PY=(1960-2026)

**PubMed**

( "Peripheral Nerve Injuries"[Mesh] OR "peripheral nerve injury"[Title/Abstract] OR "peripheral nerve injuries"[Title/Abstract] OR "digital nerve injury"[Title/Abstract] OR "digital nerve injuries"[Title/Abstract] OR "digital nerve defect"[Title/Abstract] OR "digital nerve defects"[Title/Abstract] OR "nerve gap"[Title/Abstract] OR "nerve gaps"[Title/Abstract] OR "nerve defect"[Title/Abstract] OR "nerve defects"[Title/Abstract] ) AND ( "nerve repair"[Title/Abstract] OR "nerve reconstruction"[Title/Abstract] OR "nerve regeneration"[Title/Abstract] OR conduit*[Title/Abstract] OR "nerve guidance conduit"[Title/Abstract] OR autograft*[Title/Abstract] OR "autologous nerve graft"[Title/Abstract] OR allograft*[Title/Abstract] OR "processed nerve allograft"[Title/Abstract] OR "decellularized nerve allograft"[Title/Abstract] OR "vein graft"[Title/Abstract] OR "vein conduit"[Title/Abstract] OR "direct repair"[Title/Abstract] OR neurorrhaphy[Title/Abstract] OR "connector-assisted repair"[Title/Abstract] ) AND ( compar*[Title/Abstract] OR random*[Title/Abstract] OR trial[Title/Abstract] OR cohort[Title/Abstract] ) AND ( "1900/01/01"[Date - Publication] : "2026/08/15"[Date - Publication] )

**Embase**

( 'peripheral nerve injury'/exp OR 'peripheral nerve injury':ti,ab,kw OR 'peripheral nerve injuries':ti,ab,kw OR 'digital nerve injury':ti,ab,kw OR 'digital nerve injuries':ti,ab,kw OR 'digital nerve defect':ti,ab,kw OR 'digital nerve defects':ti,ab,kw OR 'peripheral nerve defect':ti,ab,kw OR 'peripheral nerve defects':ti,ab,kw OR 'nerve gap':ti,ab,kw OR 'nerve gaps':ti,ab,kw OR 'nerve defect':ti,ab,kw OR 'nerve defects':ti,ab,kw ) AND ( 'nerve repair'/exp OR 'nerve repair':ti,ab,kw OR 'nerve reconstruction':ti,ab,kw OR 'nerve regeneration':ti,ab,kw OR conduit*:ti,ab,kw OR 'nerve guidance conduit':ti,ab,kw OR 'nerve guidance conduits':ti,ab,kw OR autograft*:ti,ab,kw OR 'autologous nerve graft':ti,ab,kw OR 'autologous nerve grafts':ti,ab,kw OR allograft*:ti,ab,kw OR 'processed nerve allograft':ti,ab,kw OR 'processed nerve allografts':ti,ab,kw OR 'decellularized nerve allograft':ti,ab,kw OR 'decellularized nerve allografts':ti,ab,kw OR 'vein graft':ti,ab,kw OR 'vein grafts':ti,ab,kw OR 'vein conduit':ti,ab,kw OR 'vein conduits':ti,ab,kw OR 'direct repair':ti,ab,kw OR neurorrhaphy:ti,ab,kw OR 'connector-assisted repair':ti,ab,kw ) AND ( 'comparative study'/exp OR 'clinical trial'/exp OR 'randomized controlled trial'/exp OR compar*:ti,ab,kw OR random*:ti,ab,kw OR trial*:ti,ab,kw OR cohort*:ti,ab,kw OR prospectiv*:ti,ab,kw OR retrospectiv*:ti,ab,kw ) AND [01-01-1900 to 15-08-2026]/pd

**Inclusion/Exclusion Criteria, Study Selection Process, and Data Extraction**

For TBI and SCI, eligible studies evaluated a pharmacological, neuroprotective, or biological intervention in patients with traumatic injury, included an appropriate concurrent comparator, and reported an extractable neurological or functional outcome. Randomized controlled trials and controlled clinical trials were eligible. The outcome used for the TBI synthesis was a favorable Glasgow Outcome Scale or Glasgow Outcome Scale-Extended result at the selected follow-up, whereas the SCI synthesis used neurological improvement by at least one grade according to the scale reported in the original study. Studies were required to provide extractable event counts and sample sizes or a corresponding effect estimate suitable for inclusion in the quantitative synthesis.

For PNI, randomized trials and comparative prospective or retrospective clinical studies evaluating nerve grafting, nerve conduits, processed nerve allografts, direct repair, connector-assisted repair, vein-based reconstruction, or other clinically relevant reconstructive approaches were eligible. Sensory recovery to Medical Research Council grade S3+ or above was the preferred outcome for the quantitative comparison. When a study used a closely related study-defined threshold, the original definition was retained and reported in Table 3 rather than being assumed to be strictly equivalent to S3+. Studies reporting motor recovery were retained in the evidence table to characterize the clinical literature but were not quantitatively combined with sensory-recovery outcomes.

Only full-text articles published in English were considered. Reviews, systematic reviews, meta-analyses, editorials, letters, conference abstracts, case reports, studies involving non-traumatic neurological conditions, uncontrolled studies, reports without extractable efficacy outcomes, duplicate publications, and studies involving overlapping patient cohorts were excluded from the primary-study synthesis. One published secondary synthesis comparing connector-assisted repair with direct repair was retained solely as the source of an already pooled descriptive estimate because a corresponding non-overlapping primary dataset was not available in the assembled evidence base. This secondary estimate was identified as such, was not treated as an independent primary trial, and was not combined again with its constituent studies.

Records retrieved from the three databases were combined and deduplicated before screening. Titles and abstracts were initially assessed against the predefined eligibility criteria, followed by full-text evaluation of potentially relevant reports. Uncertain eligibility decisions were resolved through discussion and consensus among the investigators. The identification, screening, full-text assessment, and final inclusion of studies for TBI, SCI, and PNI are summarized in Figure S1.

Data extraction was performed using a standardized framework. The extracted variables comprised publication characteristics, study design, injury definition and severity, intervention and comparator, sample size, event counts, outcome definition, and follow-up duration. For TBI and SCI, additional information on the pharmacological or biological intervention, administration regimen, and neurological or functional endpoint was recorded. For PNI, the reconstruction method, gap or defect characteristics where available, and the corresponding sensory or motor recovery outcome were additionally extracted. When multiple follow-up assessments were reported, the longest clinically interpretable follow-up that remained compatible with the selected outcome was used. Study-level characteristics, intervention and comparator definitions, event counts, endpoint definitions, and follow-up periods are presented in Tables 1-3 of the main manuscript.

A structured, design-specific methodological appraisal was performed to support interpretation of the included evidence. For randomized and controlled trials, the appraisal considered randomization procedures, allocation methods, blinding where feasible, appropriateness of the comparator, completeness of outcome reporting, and adequacy of follow-up. For non-randomized PNI studies, additional consideration was given to participant selection, baseline comparability, potential confounding, and outcome ascertainment. No composite numerical quality score was assigned. The appraisal was used to inform interpretation of the precision, generalizability, and methodological limitations of the intervention-specific estimates. Because the synthesis was intended to provide clinical context for the therapeutic gap rather than establish a definitive comparative-effectiveness hierarchy, studies with different interventions, comparators, endpoints, or follow-up periods were not assumed to be directly comparable.

**Statistical Analysis**

Odds ratios (ORs) and corresponding 95% confidence intervals (CIs) were calculated from the extracted 2 × 2 event data. When an intervention was represented by more than one eligible study, study-specific log ORs were pooled using an inverse-variance-weighted random-effects model, with between-study variance estimated using the DerSimonian–Laird method. When one arm contained zero events, a continuity correction of 0.5 was applied to all cells of the corresponding 2 × 2 table. For interventions represented by a single study, the individual study estimate and its 95% CI were displayed without pooling. Because the interventions were evaluated against different comparators and, particularly for PNI, were derived from distinct pairwise contrasts, no indirect comparison, network meta-analysis, or statistical treatment ranking was performed. Publication-bias testing was not undertaken because the number of studies available for each intervention was insufficient for meaningful assessment.

**Supplementary Method 3 Single-Cell RNA Sequencing Data Processing and Comparative Analysis**

Publicly available mouse scRNA-seq datasets were obtained from the Gene Expression Omnibus: TBI (GSE302863), SCI (GSE162610), and PNI (GSE198582). The analytical subsets were restricted to uninjured or sham tissue and the first week after injury to provide a common temporal window across the three conditions. For TBI, sham, day 1, day 3, and day 7 injured cortical samples were retained; the day 14 library available in the source series was not included in the cross-injury comparison. For SCI, uninjured spinal cord and days 1, 3, and 7 after moderate T8 contusion were included. For PNI, naive sciatic nerve and days 1, 3, and 7 after sciatic nerve crush were included. Each injury dataset was processed independently; cells were not integrated across TBI, SCI, and PNI because the source studies differed in tissue dissociation, library construction, sequencing platform, and biological sampling structure. Dataset characteristics and interpretive boundaries are summarized in Table S1.

**Quality Control, Normalization, and Clustering**

The TBI, SCI, and PNI datasets were processed separately in R using Seurat. For the TBI and SCI datasets, count matrices were converted into Seurat objects by retaining genes detected in at least three cells and cells containing at least 200 detected genes. The PNI analysis was initiated from an assembled Seurat object, whose RNA assay was converted into a Seurat v4-compatible format before reprocessing. Quality-control metrics included the number of detected genes per cell (nFeature_RNA), total unique molecular identifier counts (nCount_RNA), and the proportion of mitochondrial transcripts (percent.mt). Cells were retained when they contained more than 500 but fewer than 4,500 detected genes and had a mitochondrial transcript proportion below 15%. The distributions of nCount_RNA were examined separately for each dataset, but no universal count threshold was imposed because sequencing depth and data structure differed among the source datasets. Because the assembled PNI object had been generated from the original sample-level data after prior quality control, all 88,734 cells satisfied the specified thresholds when they were reapplied in the present workflow, and no additional cells were excluded at this step.

A uniform de novo doublet-detection procedure was not applied across all three reanalyses because the starting inputs differed in preprocessing level and availability of unfiltered droplet matrices. The source SCI publication reported the application of Scrublet independently to each sample, whereas the TBI GEO metadata indicate that multiplets were excluded during source processing without specifying the computational algorithm. The PNI analysis began from a previously assembled Seurat object, and its upstream object-level doublet classifications could not be reconstructed uniformly from the available analysis files. In the present workflow, an upper threshold of 4,500 detected genes per cell was applied, and clusters displaying incompatible lineage-marker combinations were interpreted conservatively. This feature threshold and marker-based review are not equivalent to dedicated doublet detection, and residual multiplets cannot be excluded, particularly among rare or mixed cell populations. Accordingly, no cross-injury conclusions were based solely on rare or mixed populations.

Following the initial quality-control filtering, counts were log-normalized using Seurat NormalizeData with a scale factor of 10,000. The 2,000 most variable genes in each dataset were identified using the variance-stabilizing transformation method. Cell-cycle scores were calculated for quality-control visualization but were not regressed from the expression matrix. For the PNI dataset, ambient RNA contamination was additionally estimated from the raw count matrix using decontX after initial normalization and variable-feature selection. Cells with an estimated contamination fraction of 0.30 or greater were excluded before data scaling, principal component analysis, and clustering. The retained normalized data were scaled while regressing out percent.mt, followed by principal component analysis based on the selected variable genes.

Dimensionality reduction and clustering were performed separately for each injury dataset. Harmony was applied within each dataset using the sample variable encoded in the TBI and SCI analysis objects and the group variable encoded in the PNI object. In the present analysis objects, these variables corresponded substantially or completely to injury condition or post-injury time point rather than to independent technical batches or biological replicates. Harmony-derived embeddings were therefore used only for within-dataset visualization and major-cell-class clustering and were not considered to eliminate the biological and technical differences among injury models. Potential attenuation of condition-associated variation cannot be excluded. Harmony also cannot account for differences in tissue source, injury paradigm and severity, dissociation or enrichment procedure, sequencing chemistry, cell recovery, or biological-replicate structure.

The first 20 Harmony dimensions were used to construct the shared-nearest-neighbor graphs. Cluster stability was evaluated across resolutions ranging from 0.1 to 1.0 using Clustree. Final clustering resolutions of 0.3, 0.2, and 0.2 were selected for the TBI, SCI, and PNI datasets, respectively. Uniform manifold approximation and projection and t-distributed stochastic neighbor embedding were generated from the Harmony embeddings for visualization. Quality-control, dimensionality-reduction, and cluster-resolution diagnostics for the TBI, SCI, and PNI datasets are presented in Figures S2–S4, respectively.

**Cell-Type Annotation and Cell-Composition Summaries**

Cell identities were assigned manually at the major-cell-class level according to the coordinated expression of curated canonical lineage-marker panels visualized across clusters. Annotation was based on combinations of lineage-consistent markers rather than the expression of a single marker. The TBI dataset was resolved into neuronal, oligodendrocyte precursor, oligodendrocyte, astrocyte, ependymal, fibroblast, endothelial, macrophage, microglial, neutrophil, and natural killer/T-cell populations. The SCI dataset additionally included pericyte, monocyte, and dividing-myeloid populations. The PNI dataset comprised Schwann cell, fibroblast, smooth-muscle, endothelial, macrophage, natural killer/T-cell, granulocyte, mast-cell, and dendritic-cell populations. Myeloid populations were separated only when the marker patterns supported distinction among resident microglia, infiltrating monocyte/macrophage populations, neutrophils, or other granulocytes.

For each injury dataset, descriptive cell-type abundance was calculated as the number of retained cells assigned to a given cell type divided by the total number of retained cells in the corresponding condition or post-injury time point. These proportions were used to summarize temporal and injury-associated differences in cellular composition. Because the available library structure did not provide consistent biological replication across all conditions and datasets, individual cells were not treated as independent biological replicates. No cell-level hypothesis testing or formal cross-dataset differential-abundance analysis was performed. The cell-composition patterns presented in Figure 2A–C should therefore be interpreted as descriptive and hypothesis-generating rather than as inferential estimates of population-level changes.

**Supplementary Method 4 Construction of Pathology-Related Gene Sets and Module-Score Analysis**

Six pathology-related transcriptional signatures were constructed to represent oxidative stress, neuroinflammation, excitotoxicity, necroptosis, pyroptosis, and ferroptosis. For oxidative stress, neuroinflammation, excitotoxicity, necroptosis, and pyroptosis, GeneCards was queried in August 2026 using the corresponding process name, and the 50 genes with the highest GeneCards relevance scores were retained for each signature. The ferroptosis signature was derived from the curated ferroptosis driver-gene list in FerrDB. Gene symbols were harmonized to the mouse annotation used in each expression matrix; genes absent from a dataset were omitted from scoring for that dataset.

Module scores were calculated from log-normalized expression values with Seurat AddModuleScore, which estimates the average expression of a signature relative to matched control genes selected from expression bins. Scores were calculated separately within each injury dataset and summarized by annotated cell type. For visualization, cell-type mean scores for each pathological module were standardized as Z-scores within the corresponding injury dataset. The scores therefore describe the relative enrichment of a transcriptional signature across cell populations within a dataset; they do not establish biochemical pathway activation, occurrence of a specific cell-death mechanism, or quantitative comparability of absolute activity across independently generated datasets. No cell-level hypothesis tests were used to infer sample-level differences.

**Supplementary Figures**

**Figure S1.** PRISMA flow diagrams of literature identification and study selection for the clinical intervention quantitative synthesis.

**Figure S2.**  Single-cell quality control of mouse TBI (GSE302863). **(A)** Violin plot of core cell indicators (nFeature_RNA, nCount_RNA, percent.mt) in each time point before quality control (QC). **(B)** Violin plot of core cell indicators in each time point after QC (nFeature_RNA 500~4500, percent.mt<15). **(C)** Visualization of highly variable genes. **(D)** Scatter plot after Harmony batch correction. **(E)** Elbow plot showing the variance explanation ratio of the first 50 PCs (dims = 1:20 was chosen). (F) Clustree plot for selecting the optimal clustering resolution (RNA_snn_res = 0.3 was chosen). **(G)** t-SNE plots showing cell cluster distribution.

**Figure S3.** Single-cell quality control of mouse SCI (GSE162610). **(A)** Violin plot of core cell indicators (nFeature_RNA, nCount_RNA, percent.mt) in each time point before quality control (QC). **(B)** Violin plot of core cell indicators in each time point after QC (nFeature_RNA 500~4500, percent.mt<15). **(C)** Visualization of highly variable genes. **(D)** Scatter plot after Harmony batch correction. **(E)** Elbow plot showing the variance explanation ratio of the first 50 PCs (dims = 1:20 was chosen). **(F)** Clustree plot for selecting the optimal clustering resolution (RNA_snn_res = 0.2 was chosen). (G) t-SNE plots showing cell cluster distribution.

**Figure S4.** Single-cell quality control of mouse PNI (GSE198582). **(A)** Violin plot of core cell indicators (nFeature_RNA, nCount_RNA, percent.mt) in each time point before quality control (QC). **(B)** Violin plot of core cell indicators in each time point after QC (nFeature_RNA 500~4500, percent.mt<15). **(C)** Visualization of highly variable genes. **(D)** Scatter plot after Harmony batch correction. **(E)** Elbow plot showing the variance explanation ratio of the first 50 PCs (dims = 1:20 was chosen). **(F)** Clustree plot for selecting the optimal clustering resolution (RNA_snn_res = 0.2 was chosen). **(G)** t-SNE plots showing cell cluster distribution.

**Supplementary Tables**

**Table S1.** Public single-cell datasets and analytical subsets used in the cross-injury reanalysis.

| **Injury and dataset** | **Animals and biological-replicate structure** | **Injury model and sampled tissue** | **Included time points** | **Cells before and after explicit QC** |
| --- | --- | --- | --- | --- |
| TBI GSE302863 | C57BL/6J mice. One GEO library was available for each included condition. The number of animals contributing to each library was not reported. | Controlled cortical impact; cortical tissue collected from the injury site | Sham and 1, 3, and 7 days after injury | 48,295 before QC 37,019 after QC 76.7% retained |
| SCI GSE162610 | Female C57BL/6J mice, 8–10 weeks old. Uninjured: 3 biological replicates from 5 mice; day 1: 3 replicates from 5 mice; day 3: 2 replicates from 3 mice; day 7: 2 replicates from 3 mice. | 65-kilodyne T8 contusion; 8-mm lesion-centered spinal-cord tissue | Uninjured and 1, 3, and 7 days after injury | 66,428 before QC 63,376 after QC 95.4% retained |
| PNI GSE198582 | Male and female mice on a C57BL/6 background, 8–16 weeks old. Uninjured: 2 biological replicates; day 1: 5 replicates; day 3: 6 replicates; day 7: 8 replicates. | Sciatic-nerve crush; sciatic-nerve tissue encompassing the injury site and distal nerve | Uninjured and 1, 3, and 7 days after injury | 88,734 before QC 88,734 after QC 100% retained. The merged object had undergone prior QC, and reapplication of the thresholds removed no additional cells. |

**Table S2.** Gene sets used for pathology-related transcriptional module scoring in the single-cell RNA-sequencing analyses.

| **excitotoxicity** | **ROS** | **necroptosis** | **pyroptosis** | **ferroptosis** | **neuroinflammation** |
| --- | --- | --- | --- | --- | --- |
| Ptgs2 | Hmox1 | Mlkl | Gsdmd | Rpl8 | Stat2 |
| Slc1a2 | Casp3 | Ripk1 | Gsdme | Ireb2 | Ptgs2 |
| Mapk8ip1 | Tnf | Ripk3 | Casp1 | Atp5mc3 | Adam17 |
| Srrm2 | Ager | Zbp1 | Nlrp3 | Cs | Nrros |
| Galcl | Nlrp3 | Casp8 | Gsdma | Emc2 | P2rx4 |
| Prkcg | Il6 | Cyld | Gsdmb | Acsf2 | Sphk1 |
| Dapk1 | Casp1 | Pgam5 | Nlrp1 | Nox1 | P2rx7 |
| Folh1 | Foxo3 | Ninj1 | Casp4 | Cybb | Klk6 |
| Grin2b | Nqo1 | Aim2 | Casp3 | Nox4 | Hla-f |
| Rps6ka5 | Sod1 | Pglyrp1 | Gzmb | Duox1 | Tlr4 |
| Gria1 | Becn1 | Casp1 | Aim2 | Duox2 | Dagla |
| Nfkb1 | Nfe2l2 | Nfkb1 | Casp8 | G6pd | Il1b |
| Tnf | Sqstm1 | Agfg1 | Gsdmc | Pgd | Il6 |
| Gria2 | Nos2 | Tnfsf10 | Ninj1 | Vdac2 | Tspo |
| Srr | Mapk7 | Ipmk | Cd274 | Pik3ca | Nfkb1 |
| Jun | Il1b | Fadd | Nlrc4 | Flt3 | Trem2 |
| Ccl2 | Gpx1 | Casp6 | Zbp1 | Scp2 | Bdnf |
| Cnr1 | Il18 | Spata2 | Il1b | Trp53 | Tnf |
| Tnfrsf1a | Map1lc3b | Peli1 | Il18 | Acsl4 | Fem1a |
| Grin2d | Nfkb1 | Grb2 | Card8 | Lpcat3 | Mtor |
| Cabp1 | Nox4 | Trp53 | Pycard | Nras | Ccl2 |
| Prkn | Cybb | Tlr4 | Casp5 | Kras | Cx3cl1 |
| Chat | Parp1 | Bcl2 | Gzma | Hras | Crp |
| Sod1 | Mtor | Nlrp3 | Casp7 | Tf | Nr1d1 |
| Inpp4a | Rela | Gsk3b | P2rx7 | Tfrc | Chi3l1 |
| Rnf146 | Casp9 | Map3k7 | Hmgb1 | Tfr2 | Ace2 |
| Prr7 | Alb | Gsdmd | Casp6 | Slc38a1 | Stat3 |
| Slc1a3 | Casp8 | Gpx4 | Naip | Slc1a5 | Nos2 |
| Slc24a3 | Ctnnb1 | Ace2 | Dpp9 | Gls2 | Grn |
| Parp1 | Stim1 | Mcl1 | Dpp8 | Got1 | Tlr3 |
| Mapk3 | Trp53 | Hmgb1 | Zdhhc9 | Cars1 | Glul |
| Mapk1 | Ros1 | Tlr3 | Zdhhc5 | Alox5 | Akt1 |
| Glul | Tlr2 | Itpk1 | Nfkb1 | Keap1 | Il18 |
| Casp3 | Trp53bp1 | Tnfrsf1a | Sting1 | Hmox1 | Kcnk13 |
| Nfe2l2 | Mapk1 | Parp1 | Mlkl | Atg5 | Tlr6 |
| Gdnf | Mapk3 | Traf2 | Nlrp9 | Atg7 | Trem1 |
| Psen1 | Mapk8 | Dnm1l | Cgas | Ncoa4 | Tnfrsf1a |
| Tardbp | Prkcb | Usp22 | Map3k20 | Alox12 | Tnfrsf1b |
| Calb2 | Bcl2 | Mtor | Usp48 | Alox15 | Jun |
| Fus | Ncf1 | Becn1 | Tnfrsf21 | Aloxe3 | Snca |
| Trpm2 | Bax | Tradd | Elane | Phkg2 | Nlrp3 |
| Nos1 | Trpm2 | Hmox1 | Mettl3 | Aco1 | Nfe2l2 |
| Ndp | Akr1a1 | Ninj2 | Nfe2l2 | Ulk1 | Chrna7 |
| Fos | Akt1 | Kras | Nek7 | Atg3 | Pparg |
| Il6 | Apoe | Sarm1 | Ripk3 | Becn1 | Ptgs1 |
| Grm7 | Atm | Rigi | Mefv | Map1lc3a | App |
| Ido1 | Cftr | Sox14 | Tlr4 | Gabarapl2 | Hmox1 |
| Grin1 | Ctsk | Cytl1 | Txnip | Gabarapl1 | Aqp4 |
| S100b | Drd2 | Mefv | Trp53 | Atg16l1 | Sirt1 |
| Dctn1 | Jak2 | Axl | Nlrp6 | Wipi1 | Gsk3b |
|  |  |  |  | Wipi2 |  |
|  |  |  |  | Snx4 |  |
|  |  |  |  | Atg13 |  |
|  |  |  |  | Ulk2 |  |
|  |  |  |  | Sat1 |  |
|  |  |  |  | Egfr |  |
|  |  |  |  | Mapk3 |  |
|  |  |  |  | Mapk1 |  |
|  |  |  |  | Bid |  |
|  |  |  |  | Zeb1 |  |
|  |  |  |  | Dpp4 |  |
|  |  |  |  | Cdkn2a |  |
|  |  |  |  | Pebp1 |  |
|  |  |  |  | Socs1 |  |
|  |  |  |  | Cdo1 |  |
|  |  |  |  | Myb |  |
|  |  |  |  | Mapk8 |  |
|  |  |  |  | Chac1 |  |
|  |  |  |  | Mapk14 |  |
|  |  |  |  | Prkaa2 |  |
|  |  |  |  | Prkaa1 |  |
|  |  |  |  | Elavl1 |  |
|  |  |  |  | Bap1 |  |
|  |  |  |  | Abcc1 |  |
|  |  |  |  | Acvr1b |  |
|  |  |  |  | Tgfbr1 |  |
|  |  |  |  | Epas1 |  |
|  |  |  |  | Hilpda |  |
|  |  |  |  | Hif1a |  |
|  |  |  |  | Ifng |  |
|  |  |  |  | Ano6 |  |
|  |  |  |  | Lpin1 |  |
|  |  |  |  | Hmgb1 |  |
|  |  |  |  | Tnfaip3 |  |
|  |  |  |  | Tlr4 |  |
|  |  |  |  | Atf3 |  |
|  |  |  |  | Atm |  |
|  |  |  |  | Egln2 |  |
|  |  |  |  | Miox |  |
|  |  |  |  | Tafazzin |  |
|  |  |  |  | Mtdh |  |
|  |  |  |  | Idh1 |  |
|  |  |  |  | Sirt1 |  |
|  |  |  |  | Fbxw7 |  |
|  |  |  |  | Panx1 |  |
|  |  |  |  | Dnajb6 |  |
|  |  |  |  | Bach1 |  |
|  |  |  |  | Lonp1 |  |
|  |  |  |  | Cd82 |  |
|  |  |  |  | Il1b |  |
|  |  |  |  | Ctsb |  |
|  |  |  |  | Por |  |
|  |  |  |  | Cyb5r1 |  |
|  |  |  |  | Elovl5 |  |
|  |  |  |  | Fads1 |  |
|  |  |  |  | Pten |  |
|  |  |  |  | Nr1d1 |  |
|  |  |  |  | Nr1d2 |  |
|  |  |  |  | Tbk1 |  |
|  |  |  |  | Il6 |  |
|  |  |  |  | Usp7 |  |
|  |  |  |  | Atf4 |  |
|  |  |  |  | Aqp3 |  |
|  |  |  |  | Aqp5 |  |
|  |  |  |  | Aqp8 |  |
|  |  |  |  | Pex10 |  |
|  |  |  |  | Agpat3 |  |
|  |  |  |  | Pex12 |  |
|  |  |  |  | Chp1 |  |
|  |  |  |  | Gpat4 |  |
|  |  |  |  | Brpf1 |  |
|  |  |  |  | Osbpl9 |  |
|  |  |  |  | Ints2 |  |
|  |  |  |  | Mmd |  |
|  |  |  |  | Mllt1 |  |
|  |  |  |  | Ttpa |  |
|  |  |  |  | Gria3 |  |
|  |  |  |  | Ept1 |  |
|  |  |  |  | Lig3 |  |
|  |  |  |  | Aebp2 |  |
|  |  |  |  | Agps |  |
|  |  |  |  | Cdca3 |  |
|  |  |  |  | Pex2 |  |
|  |  |  |  | Pex6 |  |
|  |  |  |  | Timm9 |  |
|  |  |  |  | Dcaf7 |  |
|  |  |  |  | Far1 |  |
|  |  |  |  | Phf21a |  |
|  |  |  |  | Smad7 |  |
|  |  |  |  | Lyrm1 |  |
|  |  |  |  | Amn |  |
|  |  |  |  | Pex3 |  |
|  |  |  |  | Mtch1 |  |
|  |  |  |  | Acadsb |  |
|  |  |  |  | Slc39a14 |  |
|  |  |  |  | Map3k11 |  |
|  |  |  |  | Gsk3b |  |
|  |  |  |  | Brd7 |  |
|  |  |  |  | Slc25a28 |  |
|  |  |  |  | Mfn2 |  |
|  |  |  |  | Slc11a2 |  |
|  |  |  |  | Tsc1 |  |
|  |  |  |  | Tgfb1 |  |
|  |  |  |  | Snca |  |
|  |  |  |  | Sirt3 |  |
|  |  |  |  | Cgas |  |
|  |  |  |  | Sting1 |  |
|  |  |  |  | Hddc3 |  |
|  |  |  |  | Mdm2 |  |
|  |  |  |  | Mdm4 |  |
|  |  |  |  | Dld |  |
|  |  |  |  | Wwtr1 |  |
|  |  |  |  | Prkca |  |
|  |  |  |  | Lgmn |  |
|  |  |  |  | Smpd1 |  |
|  |  |  |  | Mycn |  |
|  |  |  |  | Smg9 |  |
|  |  |  |  | Pparg |  |
|  |  |  |  | Snx5 |  |
|  |  |  |  | Paqr3 |  |
|  |  |  |  | Micu1 |  |
|  |  |  |  | Tor2a |  |
|  |  |  |  | Map3k14 |  |
|  |  |  |  | Mib2 |  |
|  |  |  |  | Cltrn |  |
|  |  |  |  | Klf2 |  |
|  |  |  |  | Foxo4 |  |
|  |  |  |  | Ythdc2 |  |
|  |  |  |  | Ddr2 |  |
|  |  |  |  | Slc39a7 |  |
|  |  |  |  | Trim46 |  |
|  |  |  |  | Acsl1 |  |
|  |  |  |  | Kdm5a |  |
|  |  |  |  | Trim21 |  |
|  |  |  |  | Dpep1 |  |
|  |  |  |  | Cygb |  |
|  |  |  |  | Ido1 |  |
|  |  |  |  | Gstz1 |  |
|  |  |  |  | Gja1 |  |
|  |  |  |  | Slc7a11 |  |
|  |  |  |  | Pgrmc1 |  |
|  |  |  |  | Cirbp |  |
|  |  |  |  | Usp11 |  |
|  |  |  |  | Yap1 |  |
|  |  |  |  | Trim26 |  |
|  |  |  |  | Ndrg1 |  |
|  |  |  |  | Fads2 |  |
|  |  |  |  | Piezo1 |  |
|  |  |  |  | Lifr |  |
|  |  |  |  | Egr1 |  |
|  |  |  |  | Adam23 |  |
|  |  |  |  | Cpeb1 |  |
|  |  |  |  | Cox4i2 |  |
|  |  |  |  | Timp1 |  |
|  |  |  |  | Kdm6b |  |
|  |  |  |  | Mettl14 |  |
|  |  |  |  | Mib1 |  |
|  |  |  |  | Kdm5c |  |
|  |  |  |  | Ccdc6 |  |
|  |  |  |  | Cfl1 |  |
|  |  |  |  | Kmt2d |  |
